# Global, regional, and national larynx cancer burden and health inequality analysis from 1990 to 2021 with a prediction from 2022 to 2040

**DOI:** 10.3389/fonc.2025.1617613

**Published:** 2025-07-18

**Authors:** Junjie Jiang, Zhongfang Xia, Wei Yao

**Affiliations:** Department of Otolaryngology, Wuhan Children’s Hospital, Tongji Medical College, Huazhong University of Science and Technology, Wuhan, China

**Keywords:** Global Burden of Disease (GBD), Socio-demographic Index (SDI), Bayesian Age-Period-Cohort (BAPC), risk factors, laryngeal cancer

## Abstract

**Background:**

To assess the global burden and health inequalities of laryngeal cancer from 1990 to 2021 and to project future trends up to 2040.

**Methods:**

Using the 2021 Global Burden of Disease (GBD) data, this study analyzed trends in prevalence (ASPR), incidence (ASIR), mortality (ASDR), and disability-adjusted life years (DALYs) of laryngeal cancer from 2009 to 2021, with annual percentage change (EAPC) estimates. Inequalities were assessed using the Slope Index of Inequality (SII) and Concentration Index (CI), while future projections to 2040 were made using the Bayesian Age-Period-Cohort (BAPC) model.

**Results:**

In 2021, there were approximately 1.1 million global laryngeal cancer cases (an age-standardized prevalence rate (ASPR) of 12.56 per 100,000, 95% UI: 11.76–13.49). The age-standardized incidence rate (ASIR), the age-standardized mortality rate (ASDR), and disability-adjusted life years (DALYs) rates were 2.29, 1.35, and 35.8 per 100,000, respectively. Significant disparities were observed across regions and SDI levels: high SDI regions had the highest incidence rates, while middle-low SDI regions experienced the greatest mortality and DALY burden. Central Europe had the highest ASPR, ASIR, and DALY rates, while the Caribbean reported the highest ASDR. From 1990 to 2021, the DALYs Slope Index of Inequality (SII) dropped from 37.75 (95% CI: 26.92–48.58) to -11.57 (95% CI: -22.14 to -1.00), indicating improved equity in health outcomes between high- and low-income countries.

**Conclusions:**

While the global burden of laryngeal cancer is projected to decline by 2040, significant health disparities persist. Addressing these inequalities will require sustained efforts, including equitable resource allocation, improved access to healthcare, and targeted public health interventions.

## Background

Laryngeal cancer, a malignancy originating in the larynx, is the second most prevalent cancer of the upper respiratory tract after lung cancer (1). It not only affects the respiratory tract but also interferes with important functions like voice production, protection, and swallowing. Most laryngeal cancers arise in the mucosal layer and are predominantly well-differentiated squamous cell carcinomas, while rarer types such as chondrosarcoma, leiomyosarcoma, and melanoma account for only 2% to 5% of all cases (2).

In 2022, laryngeal cancer caused over 100,000 deaths globally, with more than 200,000 new cases diagnosed. The age-standardized incidence and mortality rates were 3.9 and 2.1 per 100,000, respectively (3). According to data from the 2017 Global Burden of Disease (GBD) study, the age-standardized incidence, mortality, and disability-adjusted life year (DALY) rates of laryngeal cancer significantly declined between 1990 and 2017. However, population growth and aging led to increases of 58.67% in new cases, 33.84% in deaths, and 25% in DALYs (4). The 2019 GBD report further emphasized a continuous rise in laryngeal cancer mortality and DALYs over the past three decades, particularly in low-income countries and among middle-aged and elderly men (5).

Significant regional disparities exist in the global burden of laryngeal cancer. Developed regions exhibit higher incidence and mortality rates, primarily attributed to smoking, alcohol consumption, and occupational exposures (6). Europe shows the highest incidence rate at 5.45 per 100,000 population, with a mortality rate of 2.57 per 100,000, followed by the Americas with an incidence rate of 3.37 per 100,000 (7). In 2023, the United States reported 12,380 new cases of laryngeal cancer and 3,820 deaths (8). In contrast, less developed regions such as Africa report considerably lower incidence and mortality rates, at 0.68 and 0.61 per 100,000 population (7), respectively, likely reflecting limited healthcare resources, inadequate health education, and differing lifestyle factors.

In light of this, global strategies for the prevention and control of laryngeal cancer should prioritize the improvement of healthcare systems in low-income countries and strengthen public health policies targeting tobacco and alcohol control. Despite advances in diagnosis and treatment, the five-year survival rate for laryngeal cancer has not shown significant improvement (9), and the increasing burden poses a serious challenge to global health and economies.

Therefore, updating the global burden analysis of laryngeal cancer, with a focus on regional disparities, health inequalities, and associated risk factors, is crucial for developing precise and effective prevention and intervention strategies, optimizing health policies, and ultimately improving patient survival and quality of life.

## Methods

### Data acquisition

Data on the burden of laryngeal cancer were obtained using the Global Health Data Exchange GBD Results Tool (http://ghdx.healthdata.org/GBD-results-tool) (10, 11). The Social Development Index (SDI), created by the GBD team, measures the social and financial conditions related to health across regions (12, 13). The Socio-Demographic Index (SDI) ranges from 0 to 1, with 0 indicating the lowest level of health development and 1 representing the highest level. In this study, countries and regions were classified into five SDI tiers: low, lower-middle, middle, upper-middle, and high, to explore the relationship between the burden of laryngeal cancer and socio-economic progress. By analyzing the SDI rankings and scores of different countries, we can clearly observe the differences and development trends in socioeconomic status, education levels, and fertility rates across countries. Using the SDI classification method, this study can more accurately identify the development levels of countries and potential areas for health improvement. Based on the GBD 2021 study, we focused our analysis on four major risk factors for laryngeal cancer: smoking, alcohol consumption, and occupational exposures, which include exposure to asbestos and strong acids. By evaluating the impact of these risk factors on the DALYs and mortality rates of laryngeal cancer, our analysis revealed the direct burden of the disease and explored potential intervention strategies.

### Measurement health inequalities

To analyze inequalities, the total disability-adjusted life years (DALYs) and age-standardized DALY rates were extracted from the data. Two analytical tools, the Slope Index of Inequality (SII) and the Concentration Index (CI), were used to assess income-related disparities among countries in both absolute and relative terms (14). The SII, derived from a regression model, correlates the age-standardized DALY rate (ASDR) for laryngeal cancer with each country’s weighted ranking. To improve the accuracy of comparisons, the SII is combined with the global ASDR ratio to calculate the Relative Inequality Index (RII). The CI, which ranges from -1 to 1, measures the variation in laryngeal cancer burden across countries by fitting the Lorenz curve based on cumulative DALYs and population data. A negative CI indicates that countries with lower SDI bear a higher concentration of the burden.

### Statistical analyses

By applying the Estimated Annual Percentage Change (EAPC), this study analyzed the global trends in age-standardized incidence rates, mortality rates,disability-adjusted life years (DALYs), and prevalence of laryngeal cancer. The age-standardized rate (ASR) per 100,000 population was calculated using the following mathematical formula:


$$\text{ASR}=\frac{\sum_{\text{i}=1}^{\text{A}}{\text{a}}_{\text{i}}{\text{w}}_{\text{i}}}{\sum_{\text{i}=1}^{\text{A}}{\text{w}}_{\text{i}}}\times 100,000$$

(a_i_: The ASR (age-standardized rate) for the ith age group; w: The population size of the ith age group in the standard population; A: The total number of age groups).

The EAPC is estimated using a regression model that evaluates trends in age-standardized rates (ASR) over time (15).The regression formula is expressed as: $\text{Y}=\text{α}+\bar{\beta }\text{X}+\text{e}$ , where Y is the natural logarithm of the ASR, X represents the year, α is the intercept, β denotes the trend (slope), and e is the error term. The EAPC is calculated using the equation: EAPC = 100 × [exp(β) - 1]. This value represents the annual percentage change in ASR. To ensure its reliability, the 95% confidence interval (CI) is also computed. If both the EAPC and the lower bound of the CI are positive, it suggests an increasing trend in ASR. Conversely, if both the EAPC and the upper bound of the CI are negative, it indicates a decreasing trend. When neither condition is met, the ASR trend is considered stable. This approach provides a reliable assessment of ASR trends and their statistical validity.

This study conducted Spearman’s correlation analysis to explore the relationship between SDI and age-standardized incidence rates of laryngeal cancer. It also utilized the Bayesian Age-Period-Cohort (BAPC) model to forecast the disease’s future progression trends (16–19). The analysis was carried out using the R software package BAPC, adhering to protocols established in prior research. Data for this study was obtained from publicly accessible databases, obviating the need for clinical ethical review. Data processing and analysis were performed using R (version 4.4.0), along with Zstats v1.0 (www.zstats.net).

## Results

### Global level

**Figure 1:**
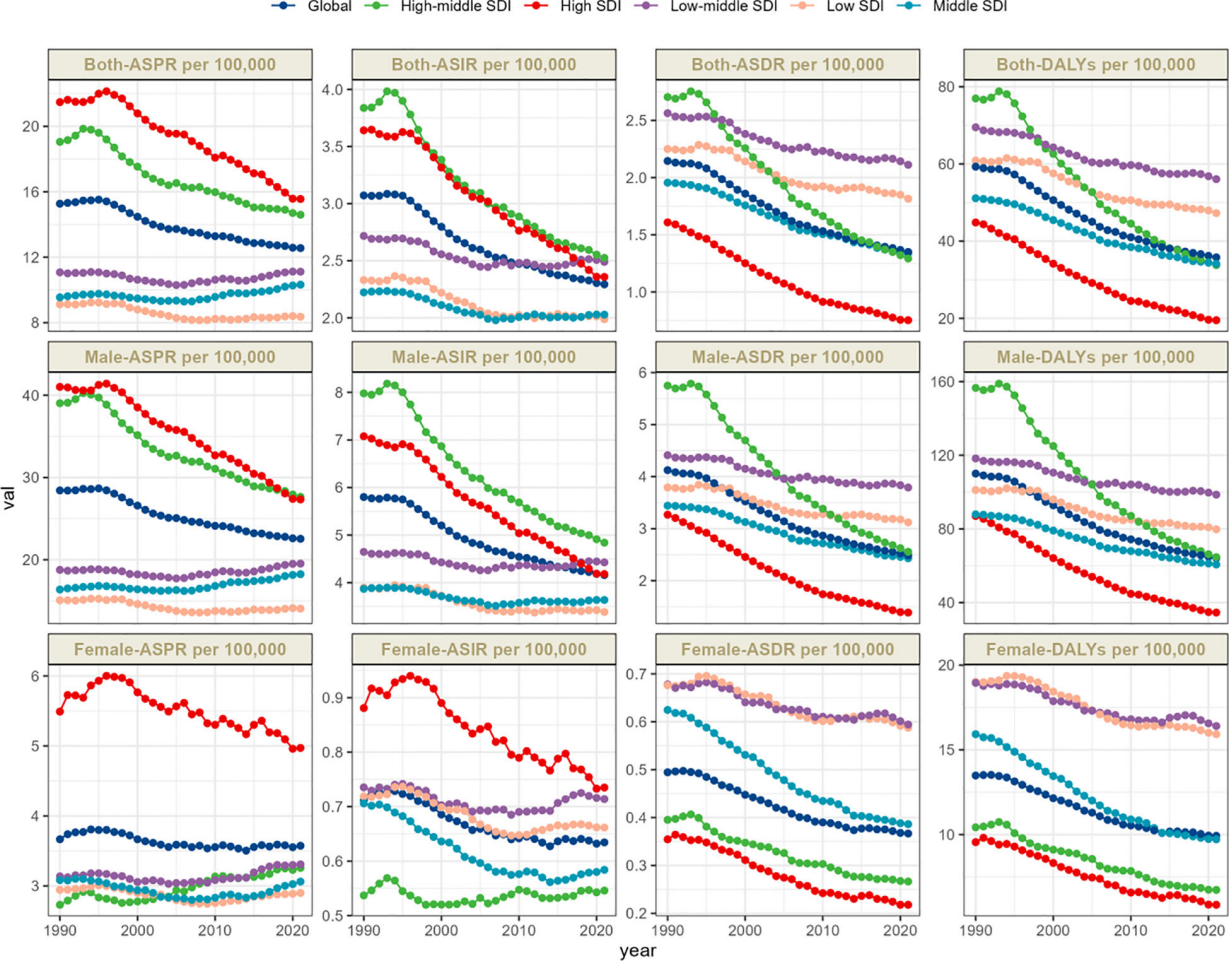
Trends in larynx cancer prevalence, incidence, deathes and disability-adjusted life-years from 1990 to 2021.

The global total number of laryngeal cancer cases in 2021 was 1,103,683.56 (95% UI: 1,033,145.34–1,186,559.53), reflecting a 75.5% increase compared to 1990. Despite the notable increase in the overall case count, the ASPR showed a downward trend, decreasing from 15.27 (95% UI: 14.54–16.06) in 1990 to 12.56 (95% UI: 11.76–13.49) in 2021. The EAPC was -0.76 (95% CI: -0.82 to -0.7) (**Table 1**, **Figure 1**). In 2021, the global incidence of laryngeal cancer reached 200,883.04 cases (95% UI: 186,941.11–216,097.56), a 60.4% increase from 1990. The ASIR decreased from 3.07 (95% UI: 2.92–3.23) in 1990 to 2.29 (95% UI: 2.13–2.47) in 2021. Throughout the analysis period, the EAPC of ASIR was -1.09 (95% CI: -1.16 to -1.01), reflecting a continuous decrease in laryngeal cancer incidence (**Table 1**, **Figure 1**). The estimated global mortality from laryngeal cancer was 117,251.6 deaths (95% UI: 109,354.57–125,952.42), with an ASDR of 1.35 (95% UI: 1.26–1.45). The EAPC for ASDR was -1.66 (95% CI: -1.74 to -1.58) (**Table 1**, **Figure 1**). The global DALYs due to laryngeal cancer totaled 3,143,308.77 (95% UI: 2,922,791.99–3,383,513.94), with an age-standardized DALY rate of 35.8 (95% UI: 33.29–38.54). The EAPC for DALYs was -1.82 (95% CI: -1.9 to -1.73) (**Table 1**, **Figure 1**).

**Table 1:** Global and regional trends in larynx cancer: Prevalence, incidence, mortality, and disability-adjusted life years (1990–2021).

Location	Number 1990	ASR 1990	Number 2021	ASR 2021	EAPC_95%CI
Prevalence					
Global	628532.28 (598813.91-660378.63)	15.27 (14.54-16.06)	1103683.56 (1033145.34-1186559.53)	12.56 (11.76-13.49)	-0.76 (-0.82 to -0.7)
High SDI	230129 (222288.89-238201.94)	21.48 (20.76-22.22)	303852.89 (288027.95-315960.18)	15.56 (14.82-16.16)	-1.15 (-1.25 to -1.06)
High-middle SDI	197763.55 (189190.36-206833.93)	19.05 (18.22-19.93)	293159.19 (265742.75-322232.29)	14.6 (13.23-16.03)	-1.03 (-1.12 to -0.94)
Middle SDI	104961.13 (95877.69-113249.57)	9.55 (8.77-10.29)	290989.56 (259494.82-326301.48)	10.32 (9.23-11.56)	0.16 (0.07 to 0.26)
Low-middle SDI	72489.02 (62965.19-84070.54)	11.08 (9.65-12.8)	168875.84 (152179.74-188225.63)	11.12 (10.03-12.38)	-0.04 (-0.14 to 0.06)
Low SDI	22279.62 (17803.15-27084.76)	9.12 (7.35-11.09)	45388.47 (39027.36-52288.09)	8.36 (7.24-9.61)	-0.42 (-0.51 to -0.32)
High-income Asia Pacific	28015.36 (25531.97-30381.05)	13.52 (12.32-14.66)	40369.73 (35690.3-44562.55)	9.33 (8.21-10.38)	-1.41 (-1.63 to -1.19)
High-income North America	83683.69 (80693.4-86227.52)	25.28 (24.42-26.03)	116920.54 (111344.36-121638.07)	18.32 (17.51-19)	-1.34 (-1.47 to -1.2)
Western Europe	154671.38 (147775.82-161269.1)	28.94 (27.7-30.16)	165571.43 (155671.88-175176.52)	20.17 (19.04-21.36)	-1.17 (-1.26 to -1.07)
Australasia	2643.58 (2436.51-2892.44)	11.48 (10.57-12.55)	3697.88 (3303.59-4060.47)	7.4 (6.58-8.18)	-1.39 (-1.48 to -1.29)
Andean Latin America	1026.64 (895.93-1170.48)	4.96 (4.33-5.66)	2217.76 (1739.9-2822.87)	3.73 (2.93-4.74)	-1.16 (-1.45 to -0.88)
Tropical Latin America	13970.61 (13388.05-14523.83)	14.24 (13.65-14.8)	39137.55 (36687.64-41463.66)	14.78 (13.86-15.65)	0.07 (-0.02 to 0.17)
Central Latin America	7862.36 (7618.2-8097.95)	9.37 (9.05-9.66)	14412.99 (12715.93-16447.39)	5.69 (5.02-6.49)	-2.08 (-2.24 to -1.92)
Southern Latin America	9460.71 (8735.91-10269.84)	20.14 (18.59-21.84)	10425.45 (9560.97-11368.81)	12.16 (11.14-13.28)	-1.76 (-1.93 to -1.58)
Caribbean	4401.65 (4073.61-4793.72)	16.81 (15.56-18.29)	11124.58 (9611.78-13026.52)	20.49 (17.72-23.95)	0.8 (0.67 to 0.92)
Central Europe	36568.96 (34593.07-38816.84)	24.04 (22.74-25.5)	50704.68 (46653.44-55168.41)	25.25 (23.18-27.54)	0.07 (-0.07 to 0.22)
Eastern Europe	64121.46 (61655.09-66742.01)	22.48 (21.59-23.44)	55020.28 (49067.21-61068.97)	16.07 (14.3-17.85)	-1.6 (-1.84 to -1.36)
Central Asia	7710.6 (7378.23-8091.12)	15.26 (14.59-16)	6821.24 (6144.08-7570.06)	7.57 (6.84-8.37)	-2.32 (-2.46 to -2.18)
North Africa and Middle East	21960.93 (18621.9-25164.5)	12.35 (10.46-14.23)	65349.35 (57925.13-74139.83)	13.76 (12.22-15.55)	0.33 (0.27 to 0.39)
South Asia	88017.94 (75934.7-102164.44)	14.03 (12.06-16.26)	211819.02 (186092.69-241891.5)	13.54 (11.9-15.43)	-0.28 (-0.42 to -0.14)
Southeast Asia	17675 (15585.64-19869.76)	6.58 (5.78-7.39)	54771.47 (47082.24-63721.64)	7.83 (6.79-9.12)	0.54 (0.48 to 0.59)
East Asia	72233.99 (59761.98-84285.25)	7.85 (6.53-9.12)	224549.9 (178186.68-280669.58)	9.83 (7.85-12.23)	0.86 (0.71 to 1.01)
Oceania	76.91 (59.06-99.62)	2.57 (2-3.27)	178.14 (138.59-231.29)	2.35 (1.84-3.01)	-0.36 (-0.41 to -0.31)
Western Sub-Saharan Africa	4456.93 (3598.18-5485.86)	4.81 (3.91-5.87)	10285.98 (8058.04-12494.23)	4.9 (3.96-5.89)	0.17 (0.11 to 0.23)
Eastern Sub-Saharan Africa	5556.69 (4379.96-6658.2)	6.83 (5.45-8.11)	10874.89 (8645.83-13991.79)	5.79 (4.65-7.35)	-0.71 (-0.78 to -0.64)
Central Sub-Saharan Africa	1531.42 (1101.26-2018.99)	6.32 (4.72-8.21)	3608.24 (2693.27-4637.09)	5.92 (4.52-7.52)	-0.2 (-0.38 to -0.02)
Southern Sub-Saharan Africa	2885.47 (2412.52-3675.8)	9.99 (8.35-12.78)	5822.46 (5137.78-6612.51)	9.26 (8.2-10.45)	-0.44 (-0.58 to -0.3)
Incidence					
Global	125175.07 (118981.12-131639)	3.07 (2.92-3.23)	200883.04 (186941.11-216097.56)	2.29 (2.13-2.47)	-1.09 (-1.16 to -1.01)
High SDI	39154.71 (37905.27-40347.82)	3.64 (3.53-3.75)	46407.47 (43637.52-48485.54)	2.36 (2.23-2.46)	-1.52 (-1.59 to -1.45)
High-middle SDI	39521.01 (37783.93-41308.8)	3.84 (3.66-4.02)	50678.71 (45728.48-55956.71)	2.53 (2.28-2.79)	-1.57 (-1.67 to -1.47)
Middle SDI	23554.33 (21565.46-25385.91)	2.22 (2.04-2.39)	56149.98 (50174.28-62743.92)	2.03 (1.81-2.27)	-0.4 (-0.48 to -0.32)
Low-middle SDI	17257.74 (14795.17-20178.95)	2.72 (2.33-3.17)	36992.11 (33138.53-41433.36)	2.49 (2.23-2.79)	-0.32 (-0.4 to -0.24)
Low SDI	5502.09 (4313.74-6752.65)	2.33 (1.83-2.86)	10395.35 (8917.55-12043.56)	1.99 (1.72-2.29)	-0.64 (-0.73 to -0.54)
High-income Asia Pacific	4554.97 (4134.72-4957.99)	2.21 (2.01-2.41)	5960.51 (5172.16-6631.27)	1.35 (1.17-1.51)	-1.87 (-2.07 to -1.66)
High-income North America	13526.58 (13093-13884.49)	4.06 (3.94-4.16)	17677.25 (16746.11-18376.15)	2.76 (2.62-2.86)	-1.55 (-1.67 to -1.44)
Western Europe	26873.91 (25775.69-27918.72)	4.98 (4.78-5.18)	24817.01 (23105.05-26412)	2.99 (2.8-3.18)	-1.63 (-1.71 to -1.55)
Australasia	481.41 (443.75-526.4)	2.08 (1.91-2.28)	581.3 (517.91-648.55)	1.15 (1.02-1.28)	-1.91 (-1.99 to -1.82)
Andean Latin America	254.06 (221.93-290.52)	1.26 (1.1-1.45)	475.3 (370.47-604.52)	0.81 (0.63-1.02)	-1.66 (-1.94 to -1.38)
Tropical Latin America	3147.1 (3019.07-3272.61)	3.29 (3.15-3.43)	7874.88 (7367.77-8341.26)	2.99 (2.8-3.17)	-0.32 (-0.41 to -0.23)
Central Latin America	1868.19 (1801.72-1927.49)	2.31 (2.22-2.39)	3058.83 (2688.63-3499.19)	1.22 (1.07-1.4)	-2.51 (-2.65 to -2.37)
Southern Latin America	1914.28 (1772.9-2080.75)	4.09 (3.79-4.44)	1854.07 (1675.85-2030.98)	2.15 (1.95-2.36)	-2.14 (-2.3 to -1.99)
Caribbean	963.38 (885.63-1050.49)	3.72 (3.42-4.05)	2209.13 (1903.87-2581.84)	4.07 (3.51-4.75)	0.43 (0.32 to 0.54)
Central Europe	7476.14 (7100.13-7912.33)	4.92 (4.67-5.2)	9083.94 (8302.14-9897.63)	4.46 (4.07-4.87)	-0.44 (-0.57 to -0.31)
Eastern Europe	13243.73 (12774.74-13731.31)	4.64 (4.46-4.81)	10194.28 (9000.86-11446.38)	2.96 (2.61-3.33)	-2.06 (-2.29 to -1.83)
Central Asia	1686.68 (1615.38-1770.47)	3.38 (3.23-3.54)	1346.95 (1200.12-1507.82)	1.52 (1.36-1.7)	-2.62 (-2.8 to -2.43)
North Africa and Middle East	4815.08 (4024.65-5571.76)	2.81 (2.34-3.3)	12005.1 (10564.46-13714.13)	2.61 (2.3-2.98)	-0.27 (-0.34 to -0.2)
South Asia	20885.24 (17693.64-24446.67)	3.44 (2.9-4.02)	45888.62 (40030.8-52451.45)	2.99 (2.61-3.42)	-0.61 (-0.74 to -0.48)
Southeast Asia	3943.17 (3413.71-4433.44)	1.52 (1.31-1.71)	10747.68 (9203.58-12634.25)	1.58 (1.36-1.86)	0.08 (0.04 to 0.12)
East Asia	15997.52 (13226.89-18749.35)	1.81 (1.51-2.12)	40050.15 (31528.22-50649.02)	1.78 (1.41-2.24)	0.02 (-0.11 to 0.16)
Oceania	17.77 (13.29-23.25)	0.64 (0.48-0.83)	40.01 (30.82-52.54)	0.56 (0.44-0.74)	-0.45 (-0.5 to -0.4)
Western Sub-Saharan Africa	1088.71 (861.12-1349.25)	1.21 (0.97-1.49)	2380.83 (1881.88-2894.78)	1.19 (0.97-1.43)	0.07 (-0.01 to 0.15)
Eastern Sub-Saharan Africa	1409.41 (1098.81-1715.56)	1.79 (1.41-2.17)	2504.95 (1968.61-3237.62)	1.38 (1.1-1.76)	-1.05 (-1.13 to -0.97)
Central Sub-Saharan Africa	392.46 (278.17-521.43)	1.7 (1.25-2.22)	853.12 (629.29-1104.46)	1.47 (1.1-1.88)	-0.47 (-0.63 to -0.3)
Southern Sub-Saharan Africa	635.27 (531.57-824.86)	2.23 (1.87-2.92)	1279.12 (1121.49-1457.57)	2.07 (1.83-2.35)	-0.46 (-0.72 to -0.21)
Deaths					
Global	85789.7 (80408.78-91207.55)	2.15 (2.01-2.28)	117251.6 (109354.57-125952.42)	1.35 (1.26-1.45)	-1.66 (-1.74 to -1.58)
High SDI	17521.86 (16915.54-18076.53)	1.61 (1.55-1.66)	15697.93 (14579.75-16475.36)	0.75 (0.71-0.79)	-2.59 (-2.68 to -2.51)
High-middle SDI	27382.23 (26032.2-28741.69)	2.7 (2.57-2.84)	25889.53 (23623.18-28383.71)	1.29 (1.18-1.42)	-2.67 (-2.77 to -2.57)
Middle SDI	19864.18 (18170.36-21395.07)	1.96 (1.8-2.1)	35944.27 (32306.89-39866.7)	1.34 (1.2-1.49)	-1.35 (-1.4 to -1.3)
Low-middle SDI	15754.02 (13442.26-18422.87)	2.56 (2.19-2.99)	30465.97 (27264.72-34217.1)	2.11 (1.9-2.37)	-0.66 (-0.72 to -0.6)
Low SDI	5132.58 (4033.15-6314.26)	2.25 (1.78-2.76)	9099.16 (7802.06-10533.93)	1.82 (1.56-2.1)	-0.79 (-0.87 to -0.71)
High-income Asia Pacific	1664.87 (1459.64-1837.25)	0.84 (0.74-0.92)	1634.09 (1403.03-1814.89)	0.31 (0.27-0.35)	-3.61 (-3.77 to -3.44)
High-income North America	4685.11 (4503.02-4811.8)	1.36 (1.31-1.4)	5057.21 (4755.08-5287.23)	0.76 (0.72-0.8)	-2.14 (-2.2 to -2.07)
Western Europe	13230.97 (12684.72-13722.46)	2.38 (2.28-2.46)	9084.66 (8333.55-9651.35)	0.99 (0.92-1.05)	-2.84 (-2.96 to -2.72)
Australasia	277.44 (255.19-305.19)	1.18 (1.09-1.3)	266.33 (233.26-297.03)	0.49 (0.43-0.54)	-2.95 (-3.04 to -2.85)
Andean Latin America	236.94 (206.99-272.01)	1.21 (1.05-1.39)	367.37 (286.05-458.12)	0.63 (0.49-0.79)	-2.27 (-2.51 to -2.03)
Tropical Latin America	2599.3 (2492.38-2705.11)	2.8 (2.68-2.92)	5593.11 (5229.86-5917.11)	2.15 (2.01-2.27)	-0.82 (-0.91 to -0.73)
Central Latin America	1644.75 (1587.4-1698.85)	2.11 (2.03-2.18)	2309.97 (2029.59-2634.05)	0.94 (0.83-1.07)	-3.02 (-3.14 to -2.9)
Southern Latin America	1429.12 (1330.95-1550.18)	3.07 (2.86-3.33)	1176.22 (1071.26-1281.61)	1.34 (1.23-1.46)	-2.64 (-2.77 to -2.51)
Caribbean	747.9 (689.58-813.9)	2.93 (2.7-3.18)	1456.89 (1264.28-1700.49)	2.69 (2.33-3.13)	-0.15 (-0.25 to -0.05)
Central Europe	5604.47 (5325.88-5920.01)	3.7 (3.51-3.9)	5322.49 (4877.04-5763.99)	2.5 (2.29-2.71)	-1.43 (-1.52 to -1.33)
Eastern Europe	9603.85 (9280.58-9918.98)	3.36 (3.25-3.48)	6043.03 (5361.33-6777.52)	1.73 (1.54-1.95)	-2.81 (-3.04 to -2.59)
Central Asia	1377.56 (1321.4-1442.11)	2.81 (2.69-2.94)	999.09 (891.2-1118.16)	1.18 (1.05-1.31)	-2.86 (-3.09 to -2.63)
North Africa and Middle East	3935 (3270.52-4563.82)	2.41 (1.99-2.83)	6991.7 (6119.12-7962.25)	1.61 (1.42-1.84)	-1.34 (-1.4 to -1.28)
South Asia	19008.78 (16065.39-22229.13)	3.25 (2.73-3.8)	37433.15 (32670.16-42840.95)	2.51 (2.2-2.87)	-0.98 (-1.07 to -0.88)
Southeast Asia	3262.32 (2830.71-3681.88)	1.31 (1.13-1.48)	7054.52 (6139.91-8306.95)	1.09 (0.95-1.28)	-0.66 (-0.69 to -0.64)
East Asia	13216.86 (10938.67-15502.85)	1.57 (1.31-1.83)	20328.09 (16104.33-25531.67)	0.93 (0.74-1.16)	-1.74 (-1.82 to -1.65)
Oceania	15.32 (11.42-20.15)	0.59 (0.45-0.77)	33.93 (25.94-44.84)	0.51 (0.4-0.68)	-0.48 (-0.52 to -0.44)
Western Sub-Saharan Africa	1023.56 (814-1268.74)	1.17 (0.94-1.44)	2119.18 (1706.55-2568.08)	1.11 (0.92-1.33)	-0.02 (-0.11 to 0.08)
Eastern Sub-Saharan Africa	1317.75 (1029.01-1608.32)	1.73 (1.36-2.1)	2200.44 (1725.31-2831.07)	1.27 (1.01-1.6)	-1.2 (-1.27 to -1.13)
Central Sub-Saharan Africa	370.07 (263.53-492.59)	1.68 (1.26-2.18)	758.17 (558.7-981.67)	1.38 (1.04-1.76)	-0.63 (-0.77 to -0.49)
Southern Sub-Saharan Africa	537.75 (451.47-697.22)	1.95 (1.64-2.53)	1021.96 (898.72-1164.93)	1.71 (1.51-1.93)	-0.63 (-0.94 to -0.33)
Disability-adjusted life years					
Global	2475842.22 (2313813.82-2636993.32)	59.3 (55.47-63.1)	3143308.77 (2922791.99-3383513.94)	35.8 (33.29-38.54)	-1.82 (-1.9 to -1.73)
High SDI	474650.13 (459363.62-490223.61)	44.84 (43.45-46.3)	374680.05 (353341.75-392842.13)	19.52 (18.47-20.42)	-2.81 (-2.89 to -2.74)
High-middle SDI	800358.31 (762946.56-840011.96)	76.97 (73.37-80.79)	672171.11 (610773.14-738584.64)	33.81 (30.72-37.12)	-2.99 (-3.11 to -2.88)
Middle SDI	575232.87 (525692.15-622085.81)	51.12 (46.83-55.19)	961528.98 (863184.64-1070151.17)	34.12 (30.71-37.96)	-1.45 (-1.5 to -1.39)
Low-middle SDI	468502.95 (400031.54-548586.25)	69.5 (59.39-81.33)	865607.99 (772731.93-976130.77)	56.07 (50.13-63.17)	-0.73 (-0.78 to -0.67)
Low SDI	153217.3 (119609.44-188657.58)	60.89 (47.73-75.04)	265293.29 (226143.41-308264.02)	47.26 (40.5-54.8)	-0.96 (-1.04 to -0.88)
High-income Asia Pacific	42366.68 (36682.18-47460.08)	20.57 (17.86-23.02)	31591.08 (27473.12-35245.16)	7.1 (6.22-7.97)	-3.83 (-3.98 to -3.68)
High-income North America	122557.9 (118367.98-126136.11)	37.33 (36.09-38.39)	124213.61 (118353.96-129908.17)	19.77 (18.88-20.65)	-2.31 (-2.38 to -2.24)
Western Europe	354612.92 (339961.97-369065.24)	67.26 (64.52-70)	210237.34 (195734.22-223427.18)	25.72 (24.02-27.36)	-3.14 (-3.24 to -3.03)
Australasia	7115.09 (6486.89-7826.67)	30.91 (28.09-34.02)	5843.74 (5186.85-6521.43)	11.53 (10.23-12.91)	-3.23 (-3.32 to -3.13)
Andean Latin America	6123.71 (5332.41-7015.25)	28.77 (25.02-32.95)	8755.54 (6758.21-10981.12)	14.65 (11.31-18.37)	-2.42 (-2.68 to -2.16)
Tropical Latin America	77184.61 (74117-80182.43)	77.36 (74.31-80.4)	155325.81 (145994.06-164600.08)	58.65 (55.12-62.14)	-0.92 (-1.02 to -0.81)
Central Latin America	42062.42 (40672.71-43347.54)	49.13 (47.41-50.69)	55571.44 (48791.54-63689.49)	21.88 (19.22-25.07)	-3.06 (-3.18 to -2.93)
Southern Latin America	40070.78 (37170.2-43556.44)	85.38 (79.24-92.86)	28982.03 (26307.66-31799.54)	34 (30.86-37.32)	-3.02 (-3.16 to -2.88)
Caribbean	19082.14 (17577.49-20742.96)	72.57 (66.93-78.93)	36861.47 (31615.95-43355.47)	67.98 (58.33-79.96)	-0.04 (-0.14 to 0.06)
Central Europe	167827.27 (159550.26-177662.66)	111.11 (105.61-117.71)	140094.17 (127656.78-152554.94)	69.93 (63.58-76.15)	-1.69 (-1.8 to -1.57)
Eastern Europe	302200.05 (292438.37-312362.98)	106.81 (103.18-110.57)	172245.09 (152566.87-193400.24)	51.29 (45.47-57.66)	-3.09 (-3.32 to -2.85)
Central Asia	42704.84 (40929.12-44722.16)	83.67 (80.18-87.67)	29421.89 (26003.05-33078.19)	32.31 (28.65-36.25)	-3.2 (-3.42 to -2.97)
North Africa and Middle East	112667.7 (93508.39-130088.12)	61.6 (51.28-71.34)	191928.94 (166569.44-219659.61)	39.45 (34.38-45.16)	-1.51 (-1.56 to -1.45)
South Asia	575770.42 (490461.69-672476.67)	88.93 (75.45-103.97)	1066560.26 (929501.43-1228037.22)	67.29 (58.66-77.4)	-1.05 (-1.14 to -0.95)
Southeast Asia	93619.74 (81149.24-105815.36)	33.9 (29.46-38.31)	194228.13 (167982.6-230360.97)	27.61 (23.9-32.69)	-0.72 (-0.74 to -0.7)
East Asia	372617.38 (306394.99-438644.27)	39.94 (33.02-46.95)	508207.46 (397895.99-639685.81)	22.59 (17.73-28.27)	-1.9 (-1.99 to -1.81)
Oceania	438.91 (321.96-585.33)	14.13 (10.55-18.58)	949.47 (717.3-1263.33)	12.02 (9.21-15.94)	-0.56 (-0.6 to -0.52)
Western Sub-Saharan Africa	29529.67 (23182.75-36886.59)	31.11 (24.58-38.66)	60794.41 (47457.73-74635.06)	27.96 (22.29-33.92)	-0.25 (-0.34 to -0.16)
Eastern Sub-Saharan Africa	39797.25 (30691.02-48676.96)	47.38 (36.92-57.79)	67635.53 (52442.3-88562.51)	34.49 (27.03-44.42)	-1.24 (-1.31 to -1.16)
Central Sub-Saharan Africa	10970.16 (7661.63-14610.1)	43.75 (31.29-57.68)	22969.88 (16740.01-29889.17)	36.27 (26.7-46.84)	-0.6 (-0.73 to -0.47)
Southern Sub-Saharan Africa	16522.56 (13881.29-21161.24)	55.59 (46.65-71.65)	30891.49 (26811.15-35636.07)	48.13 (41.99-55.22)	-0.69 (-1 to -0.38)

### Regional level

Laryngeal cancer burden shows considerable variation across regions, strongly associated with the level of the SDI. The ASPR shows marked differences, with the highest prevalence in high-SDI regions at 15.56 per 100,000 people (95% UI: 14.82–16.16), and the lowest in low-SDI regions at 8.36 per 100,000 people (95% UI: 7.24–9.61) (**Table 1**, **Figure 1**).

The time trends of ASPR reveal distinct patterns linked to SDI levels, potentially reflecting different phases of public health evolution. High-SDI regions experience the most substantial reduction, with an EAPC of -1.15 (95% CI: -1.25 to -1.06). In these areas, the ASPR fell from 21.48 (95% UI: 20.76–22.22) in 1990 to 15.56 (95% UI: 14.82–16.16) in 2021, indicating the success of preventive measures and healthcare interventions (**Table 1**, **Figure 1**).

On the other hand, regions with middle SDI showed the most significant rise in EAPC, at 0.16 (95% CI: 0.07 to 0.26), reflecting an ongoing upward trend in laryngeal cancer cases in these regions (**Table 1**, **Supplementary Figure S1A**). This pattern may stem from a combination of influences, including improvements in medical technology that facilitate early diagnosis, an aging population contributing to a larger pool of high-risk individuals, and environmental exposures linked to rapid urban development and shifting lifestyles. In these transitioning economies, factors like increased tobacco and alcohol use, occupational hazards, prolonged exposure to physical irritants (e.g., noise or chemicals at work), Human Papillomavirus (HPV) infections, genetic predispositions, and age are all closely associated with the rising incidence of laryngeal cancer.

**Figure 2:**
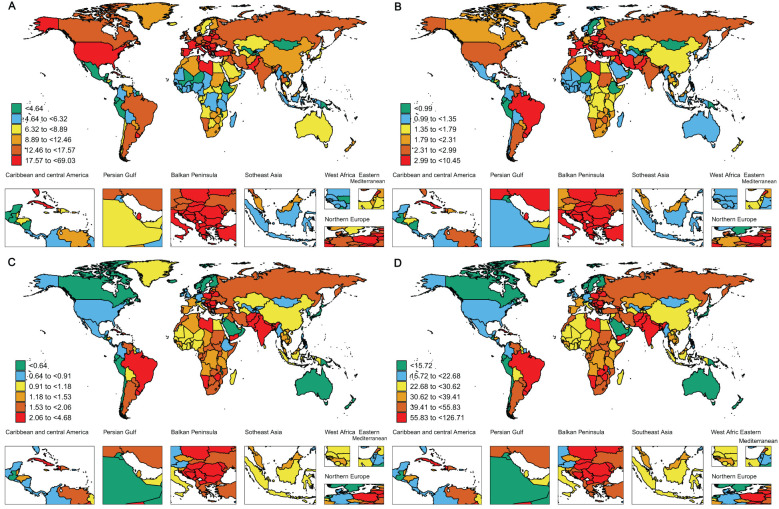
The global disease burden of larynx cancer for both sexes in 204 countries and territories. **(A)** Prevalence rate. **(B)** Incidence rate. **(C)** Death rate. **(D)** DALYs rate.

Both the ASIR and ASDR further highlight regional disparities. High-middle-SDI regions exhibit the highest ASIR, whereas low-SDI regions show the lowest. Specifically, the ASIR in high-middle-SDI regions is 2.53 (95% UI: 2.28–2.79), while high-SDI regions have a lower rate of 1.99 (95% UI: 1.72–2.29). At the same time, middle-low-SDI regions report the highest ASDR, with high-SDI regions having the lowest. The ASDR in middle-low-SDI regions is 2.11 (95% UI: 1.9–2.37), while in high-SDI regions, it is substantially lower at 0.75 (95% UI: 0.71–0.79) (**Table 1**, **Figures 1**, **2C**).This significant difference not only reveals the disparity in the prevalence of risk factors but also reflects differences in acute medical management, the implementation of secondary prevention measures, and public health strategies, all of which collectively influence the distribution of disease burden.

The age-standardized Disability-Adjusted Life Years (DALY) rate highlights these regional differences even more. Middle-low-SDI regions experience the greatest burden, with a DALY rate of 56.07 (95% UI: 50.13–63.17), while high-SDI regions report the lowest, with a rate of 19.52 (95% UI: 18.47–20.42) (**Table 1**, **Figures 1**, **2D**). These results collectively underscore the intricate connection between socio-demographic factors and the outcomes of laryngeal cancer. While high-SDI regions have achieved substantial progress in lowering both the prevalence and burden of laryngeal cancer, regions with middle and high-middle SDI are encountering progressively more significant obstacles.

The results of our study reveal that Central Europe carries the greatest burden of laryngeal cancer prevalence worldwide. Notably, Central Europe exhibits the highest ASPR, reaching 25.25 (95% UI: 23.18–27.54), with the Caribbean coming next at 20.49 (95% UI: 17.72–23.95) (**Table 1**, **Figure 2A**). Western Europe also shows a relatively high ASPR of 20.17 (95% UI: 19.04–21.36), ranking third among the regions examined. The elevated prevalence of laryngeal cancer in Central Europe may result from a combination of factors, such as higher rates of smoking, alcohol consumption, occupational exposures, and advancements in early detection. Additionally, the higher proportion of elderly populations in the region, coupled with the increased incidence of laryngeal cancer with age, may further contribute to the elevated prevalence. Furthermore, higher rates of diabetes and hypertension may also be strongly associated with the incidence of laryngeal cancer. In contrast, the high ASPR in the Caribbean may be related to dietary habits, lifestyle changes, and increased environmental and health risks associated with rapid urbanization. These factors may contribute to the high incidence of laryngeal cancer in the region. Similarly, Central Europe, the Caribbean, and Western Europe report the highest ASIRs, with Central Europe at 4.46 (95% UI: 4.07–4.87), the Caribbean at 4.07 (95% UI: 3.51–4.75), and Western Europe at 2.99 (95% UI: 2.8–3.18). In contrast, Oceania has the lowest ASIR at 0.56 (95% UI: 0.44–0.74) (**Table 1**, **Figure 2B**).

The time trends from 1990 to 2021 reveal varying patterns of change across different global regions. The Caribbean experienced the most significant rise in ASIR, with an EAPC of 0.43 (95% CI: 0.32 to 0.54), whereas Central Asia saw the sharpest decline, with an EAPC of -2.62 (95% CI: -2.8 to -2.43) (**Table 1**, **Supplementary Figure S1B**). These divergent trends emphasize the complex and region-specific nature of laryngeal cancer incidence rates, highlighting the importance of region-tailored interventions and further research to understand and address these disparities.

The ASDR for laryngeal cancer is significantly higher in the Caribbean, South Asia, and Central Europe, with all regions recording rates above 2.5 per 100,000 people. From 1990 to 2021, a downward trend was observed globally in the ASDR for laryngeal cancer, with the steepest declines noted in high-income populations of the Asia-Pacific region (EAPC -3.61, 95% CI: -3.77 to -3.44). Australasia and Western Europe also showed substantial decreases (EAPC -2.95, 95% CI: -3.04 to -2.85 and EAPC -2.84, 95% CI: -2.96 to -2.72, respectively) (**Table 1**, **Supplementary Figure S1C**). In terms of age-standardized DALY rates, Central Europe, the Caribbean, and South Asia emerge as the most affected regions. Central Europe leads with 69.93 per 100,000 people (95% UI: 63.58–76.15), followed by the Caribbean at 67.98 per 100,000 people (95% UI: 58.33–79.96), and South Asia at 67.29 per 100,000 people (95% UI: 58.66–77.4) (**Table 1**, **Figure 2D**).

Between 1990 and 2021, age-standardized DALY rates for laryngeal cancer showed a decline across all 21 global regions. The most pronounced reductions occurred in high-income Asia-Pacific regions (EAPC -3.83, 95% CI: -3.98 to -3.68), followed by Australasia (EAPC -3.23, 95% CI: -3.32 to -3.13) and Western Europe (EAPC -3.14, 95% CI: -3.24 to -3.03) (**Table 1**, **Supplementary Figure S1D**). These findings indicate an inverse relationship between age-standardized DALY rates and SDI levels (**Supplementary Figure S2**).

**Figure 3:**
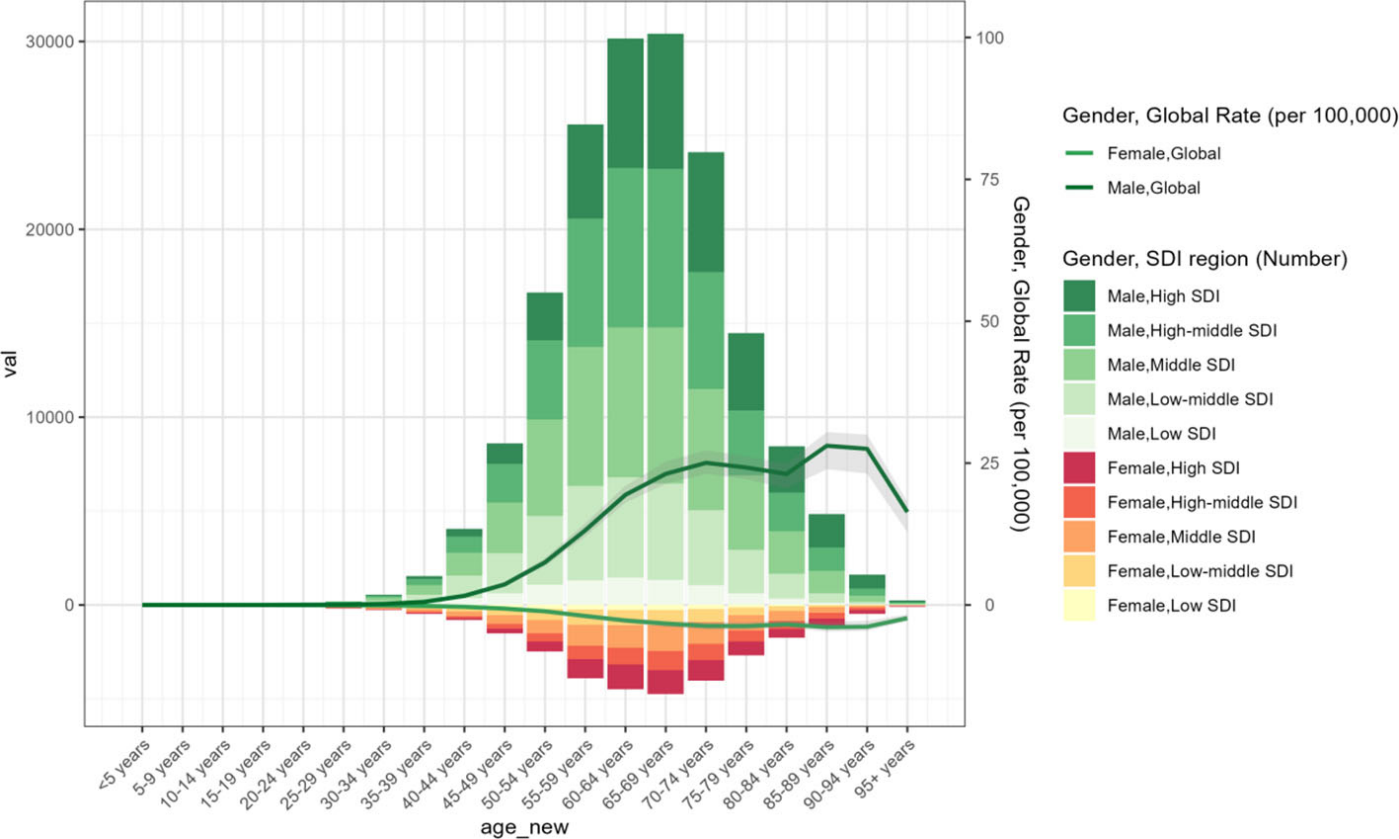
The age-specific numbers and ASIRs of larynx cancer by SDI regions in 2021.

From 1990 to 2021, the incidence of laryngeal cancer demonstrated a clear increase with advancing age, particularly among men aged 50 years and older. High-SDI regions showed a greater concentration of laryngeal cancer cases among the elderly, while reporting a smaller proportion of cases in individuals below 50 years (**Figure 3**). Overall, the incidence of laryngeal cancer was markedly higher in older populations compared to younger ones across all SDI regions (**Supplementary Figure S3**).

### National level

The ASPR of laryngeal cancer shows considerable variability across countries, ranging from approximately 0.89 to 69.02 per 100,000 people. Monaco, Montenegro, and Cuba have the highest ASPR, with Monaco reporting 69.02 per 100,000 (95% UI: 52.52–93.39), Montenegro 49.48 per 100,000 (95% UI: 38.74–64.64), and Cuba 40.96 per 100,000 (95% UI: 34.04–49.37) (**Figure 2A**, **Supplementary Table S1**). Notably, Monaco is in Western Europe, and Cuba is in the Caribbean, both of which are regions with some of the highest rates of laryngeal cancer prevalence globally. Consistent with these findings, our regional analysis also reveals that Western Europe and the Caribbean are among the areas with the most elevated laryngeal cancer incidence rates, further emphasizing the significant challenges these areas face in addressing the disease.

Romania has recorded the sharpest rise in ASPR (EAPC: 1.59, 95% CI: 1.41–1.77), while Uzbekistan exhibited the steepest drop in ASPR (EAPC: -3.13, 95% CI: -3.65 to -2.62) (**Supplementary Figure S1A**, **Supplementary Table S1**). The global distribution of the ASIR is illustrated in **Figure 2B**, with detailed data in **Supplementary Table S2**. Monaco reports the highest ASIR at 10.44 per 100,000 (95% UI: 7.8–14.22), while Kiribati has the lowest at 0.23 per 100,000 (95% UI: 0.16–0.32). These findings highlight the significant global epidemiological differences in laryngeal cancer, with particular emphasis on countries like Romania, where the substantial rise in both incidence and prevalence may point to notable disparities in environmental exposures, medical infrastructure, and public health initiatives.

ASDR and DALYs in 2021 indicate a persistent pattern in the nations hardest hit by laryngeal cancer. Montenegro, Cuba, and Pakistan are notably high on both measures. Montenegro reports the highest ASDR at 4.67 per 100,000 (95% UI: 3.61–6.1), followed by Cuba at 4.65 per 100,000 (95% UI: 3.93–5.61), and Pakistan at 4.58 per 100,000 (95% UI: 3.35–6.04) (**Figure 2C**, **Supplementary Table S3**). For DALYs, Montenegro leads with 127.61 per 100,000 (95% UI: 97.86–170.11), followed by Pakistan at 121.47 per 100,000 (95% UI: 88.73–161.68), and Cuba in third place with 117.58 per 100,000 (95% UI: 97.77–142.57) (**Figure 2D**, **Supplementary Table S4**). This convergence of high ASDR and DALY rates suggests that these nations bear an exceptionally heavy burden of laryngeal cancer, affecting not only death rates but also the general quality of health and life.

South Korea (EAPC: -5.95, 95% CI: -6.28 to -5.62), Singapore (EAPC: -5.15, 95% CI: -5.48 to -4.81), and Kuwait (EAPC: -3.9, 95% CI: -4.48 to -3.31) have shown the steepest declines in age-standardized DALYs attributed to laryngeal cancer **Supplementary Figure S1D**, **Supplementary Table S4**).

**Figure 4:**
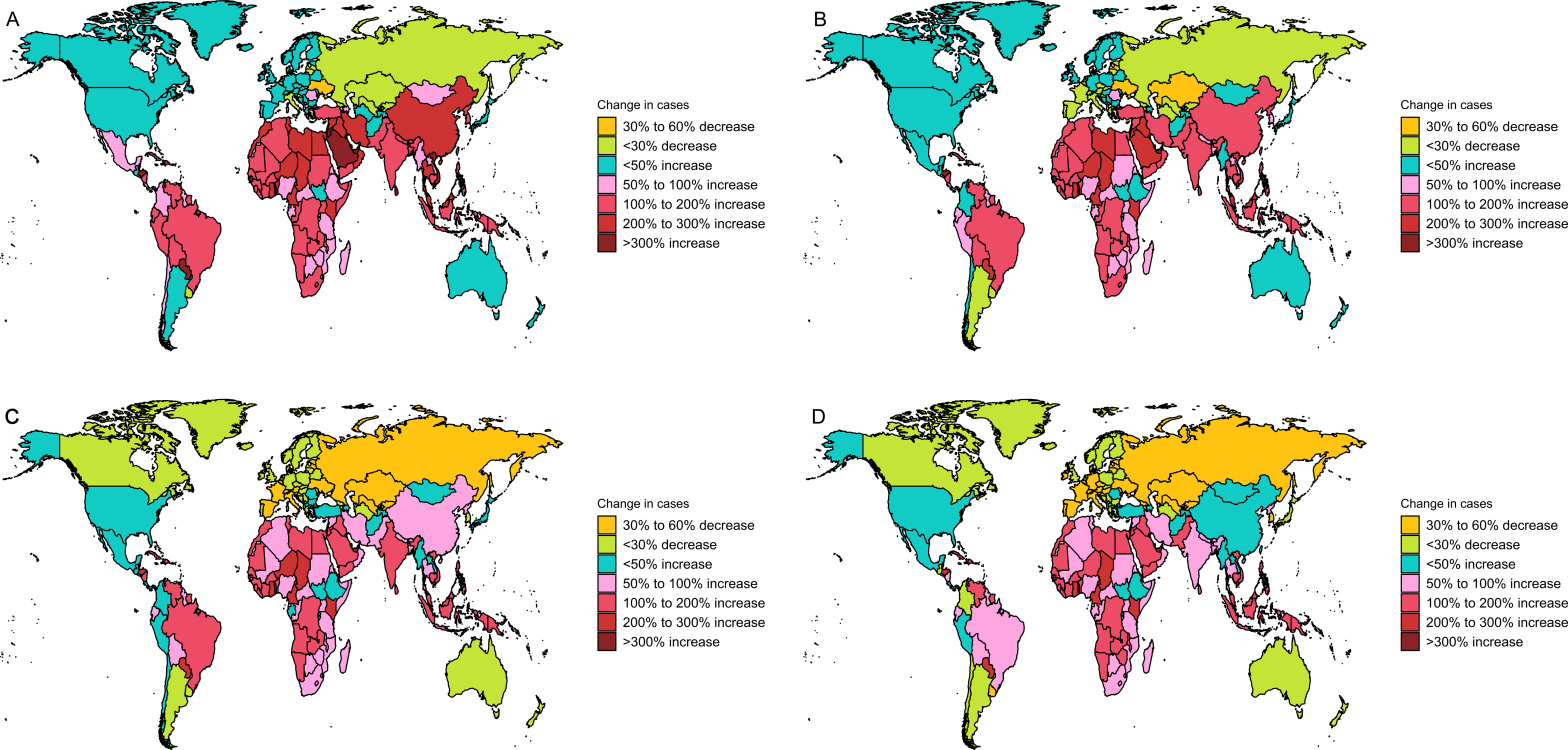
Change cases of larynx cancer for both sexes in 204 countries and territories. **(A)** Change prevalence cases. **(B)** Change incidence cases. **(C)** Change deaths cases. **(D)** Change DALYs.

Regionally, the United Arab Emirates (UAE) recorded an extraordinary surge in laryngeal cancer cases, with a 674% increase, while Ukraine saw a 35% reduction (**Figure 4A**). The UAE also exhibited the sharpest rise in incidence, growing by 540%, as opposed to Ukraine, which experienced a 40% decline (**Figure 4B**). In terms of mortality, the UAE had the most pronounced growth, with a 352% escalation, whereas Armenia achieved a 35% drop (**Figure 4C**). Additionally, DALYs in the UAE surged by 361%, marking the largest increase, while Belgium achieved a notable 51% reduction (**Figure 4D**).

These observations highlight stark regional and national variations in the burden of laryngeal cancer, emphasizing the need for focused healthcare policies, effective preventive measures, and further investigation to understand and mitigate the diverse epidemiological trends and underlying risk factors.

### Age and sex patterns

**Figure 5:**
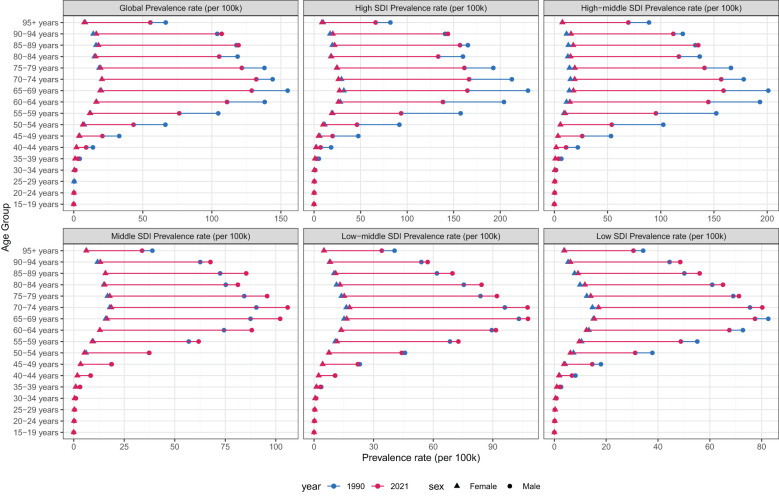
Age-standardized prevalence rates of larynx cancer by sex, age group, and socio-demographic index, 1990 and 2021.

In 2021, the ASPR of laryngeal cancer increased steadily with age, reaching its highest levels in the 60–79 age group (**Figure 5**). Males consistently showed higher ASPR values compared to females within the same age range. Similarly, the age-standardized incidence rate (ASIR) of laryngeal cancer rose progressively with age, peaking in individuals aged 85 and above (**Supplementary Figure S4**). Male incidence rates were uniformly greater than those of females.

Compared to 1990, the age-standardized death rate (ASDR) for laryngeal cancer showed an overall decline in both genders by 2021. Nevertheless, mortality rates exhibited an upward trend with advancing age, and across nearly all age groups, males had consistently higher ASDRs than females (**Supplementary Figure S5**). Moreover, while the overall age-standardized mortality rate (ASR) dropped relative to 1990, males continued to have a higher age-standardized mortality rate (ASMR) compared to females (**Supplementary Figure S6**).

These findings emphasize the growing impact of laryngeal cancer with aging, particularly among men, highlighting that both age and gender remain critical factors influencing the disease’s incidence and mortality rates. The persistently higher mortality rate in men underscores the importance of developing gender-specific prevention and treatment approaches.

### Risk factors for larynx cancer

This study gathered data on laryngeal cancer-related deaths and DALYs, examining the impact of four key risk factors: smoking, alcohol consumption, and occupational exposures (including sulfuric acid and asbestos). Globally, tobacco use emerged as the primary driver of deaths and DALYs linked to laryngeal cancer in 2021. Notably, smoking was responsible for 66.5% of all laryngeal cancer deaths (**Supplementary Figure S7**). Regionally, Eastern Europe reported the largest share of tobacco-attributable deaths at 79.8%, whereas the lowest proportion was observed in Western Sub-Saharan Africa, at 34.8%.

Alcohol use was another key factor, contributing the most in Central Europe (23.5%) and the least in North Africa and the Middle East (1.9%). Occupational exposures also had a notable impact on laryngeal cancer mortality, with sulfuric acid exposure accounting for the highest proportion of deaths in Southeast Asia (3.8%) and the lowest in Southern Sub-Saharan Africa (1.4%). Asbestos exposure was most prominent in Australasia (17.9%), whereas the smallest share was recorded in Eastern and Central Sub-Saharan Africa (0.8%).

The influence of these risk factors differed across SDI regions. In comparison to low SDI regions, high-middle SDI regions recorded a greater share of deaths linked to smoking, whereas high SDI regions showed a higher percentage of deaths associated with alcohol consumption.

On a global scale, tobacco use emerged as the leading risk factor for DALYs, contributing 65.6% of all laryngeal cancer-related DALYs (**Supplementary Figure S8**). Regionally, Eastern Europe recorded the largest share of tobacco-attributable DALYs at 80.8%, whereas Western Sub-Saharan Africa had the smallest share at 35.2%. Alcohol use was another key contributor, with Central Europe accounting for 24.5% of DALYs, contrasting with North Africa and the Middle East at just 2.1%.

Sulfuric acid exposure significantly influenced DALYs, with East Asia reporting the greatest impact at 4.9% and Southern Sub-Saharan Africa the lowest at 1.6%. Similarly, asbestos exposure contributed notably, with Australasia leading at 14.3% and Eastern Sub-Saharan Africa at the bottom with 0.6%.

Among the five SDI regions, tobacco-related DALYs were most prevalent in middle-high SDI regions (77.3%) and least common in low SDI regions (61%). The effects of other risk factors on DALYs varied across these regions, highlighting the complexity of the global laryngeal cancer burden.

These findings underscore the intricate relationship between risk factors, regional characteristics, and the global burden of laryngeal cancer, pointing to the importance of tailored prevention and intervention strategies for different regions based on specific risk profiles.

### Global health inequality in laryngeal cancer

**Figure 6:**
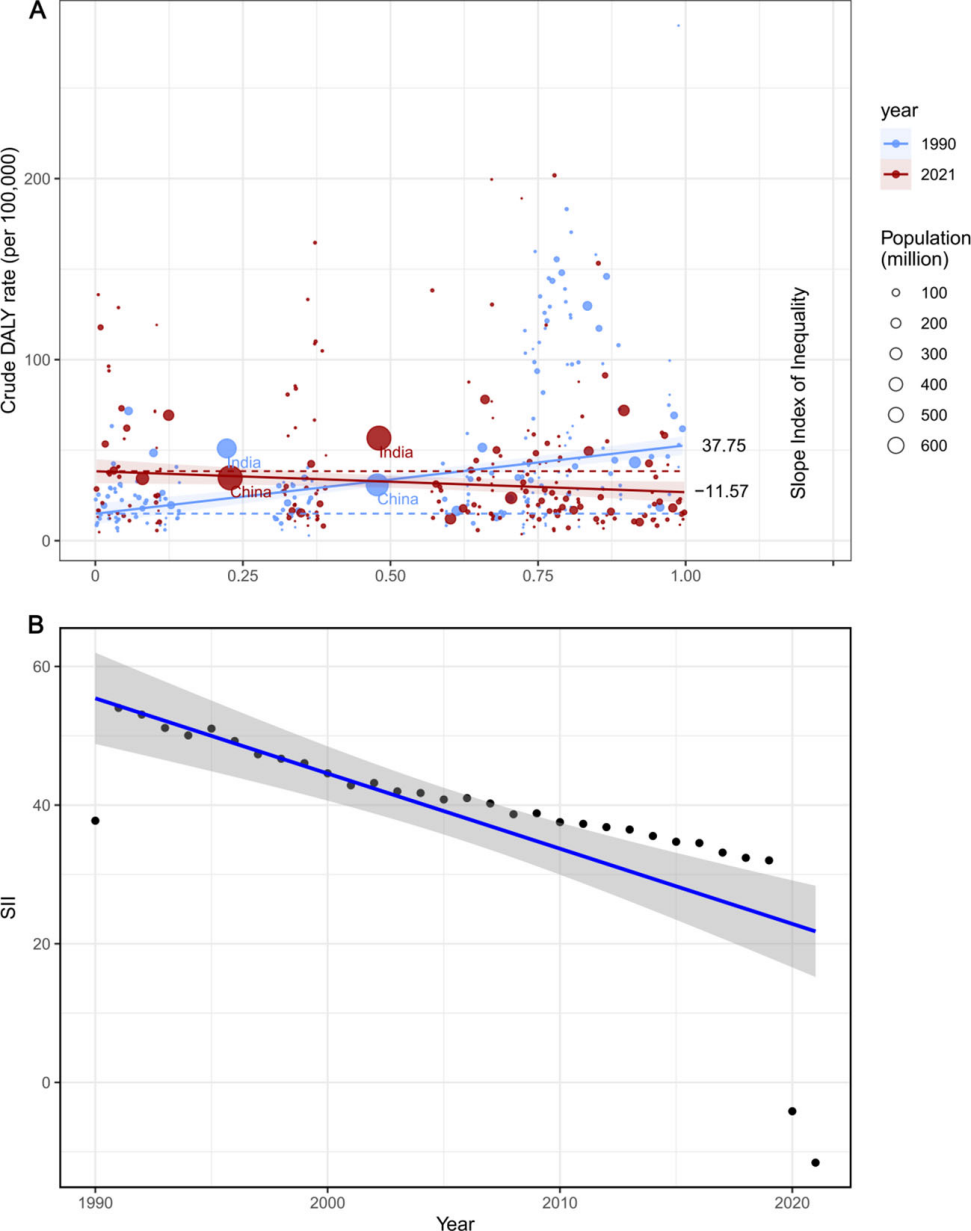
SII analysis. **(A)** Absolute income- related healthy inequality in larynx cancer burden, presented using regression lines, 1990 *vs*. 2021. **(B)** Trendline demon strates the trend in SII from 1990 to 2021.

From 1990 to 2021, the SII (Slope Index of Inequality) for DALYs per 100,000 people declined from 37.74861 (95% CI: 26.915933–48.58129) to -11.56948 (95% CI: -22.13804 to -1.000919). Over this period, the correlation between SDI and age-standardized DALY rates shifted from positive to negative, reflecting the influence of improvements in healthcare resources and socio-economic development on disease burden (**Figure 6**). The reduction in SII further indicates that health inequality in laryngeal cancer between higher- and lower-income countries has lessened.

Despite the overall decrease in the Concentration Index (CI) for disability and mortality, global inequities in the burden of laryngeal cancer remain pronounced. Although economic gaps have narrowed in certain areas, disparities in health outcomes due to laryngeal cancer continue to pose challenges. Sustained efforts to address this issue, emphasizing fair access to healthcare and prevention strategies, are critical (**Supplementary Figure S8**). These findings underscore that while progress has been made in reducing the unequal global distribution of health outcomes for laryngeal cancer, disparities across regions and income levels persist, highlighting the importance of continued focus and investment to tackle this significant public health concern.

### Future forecasts of global burden of larynx cancer

Between 2021 and 2040, the global burden of laryngeal cancer is projected to shift significantly, with diverse trends observed across key indicators. The overall ASPR for both sexes is expected to decline by approximately 10.36%, dropping from 12.56 to 11.26 per 100,000 individuals (**Supplementary Figure S9**). Among men, the prevalence rate is anticipated to decrease markedly from 22.54 to 19.89, representing an 11.77% reduction. In contrast, women are projected to experience a much smaller decline, with rates decreasing slightly from 3.57 to 3.54, a drop of only 0.84%.

Similarly, the global ASIR is forecasted to decline modestly, from 12.56 to 11.25. The combined ASDR for both sexes is predicted to fall from 2.14 to 1.74, reflecting potential advancements in treatment and management of laryngeal cancer. DALYs are also expected to see a substantial decrease, shrinking from 35.8 to 29.28, indicating improvements in the overall health impact of the disease.

Throughout this period, men are projected to consistently exhibit higher rates across all indicators compared to women. Furthermore, the disparity between sexes is expected to grow over time, particularly in terms of DALYs, highlighting persistent gender-related differences in the burden of laryngeal cancer.

## Discussion

This study utilizes the 2021 Global Burden of Disease (GBD) data to provide updated insights into the burden of laryngeal cancer, covering global data from 1990 to 2021 across 204 countries and regions. In 2021, the global burden of laryngeal cancer included 1.1 million prevalent cases, 200,000 new diagnoses, nearly 120,000 deaths, and 3.1 million Disability-Adjusted Life Years (DALYs). While age-standardized incidence rates, mortality rates, and DALYs have declined over the past three decades, the absolute number of cases continues to rise, primarily driven by factors such as population expansion, global aging, and increased life expectancy. These results are consistent with earlier GBD studies, which similarly observed a steady reduction in age-standardized incidence rates for laryngeal cancer over time (4).

Despite the declining trends in all four indicators for laryngeal cancer, significant regional disparities remain. The highest age-standardized prevalence rate (ASPR) and age-standardized incidence rate (ASIR) are observed in high and upper-middle SDI regions, reflecting the uneven distribution of laryngeal cancer across different regions and the complex and diverse nature of its global impact. Conversely, the lowest ASPR and ASIR are found in low SDI regions, which may be closely linked to economic conditions and lifestyle factors. In contrast, high SDI regions report lower age-standardized death rates (ASDR) and DALY rates, primarily due to stronger healthcare infrastructure, advanced medical technology, effective public health measures, and higher health awareness and education levels (20, 21).

Specifically, Central Europe has the highest incidence rate of laryngeal cancer, consistent with previous research findings (22),and is closely associated with high smoking rates and alcohol consumption (23, 24). Meanwhile, the burden of laryngeal cancer in the Caribbean remains high, potentially linked to dietary habits, lifestyle changes, and environmental and health risks associated with rapid urbanization. Oceania shows the lowest ASIR, which can be attributed to successful tobacco control measures, increased health awareness, and advancements in healthcare. Incidence rates in high-income Asia-Pacific regions, Oceania, and Western Europe have significantly decreased, likely due to healthier lifestyles and superior social welfare and healthcare services. Further analysis reveals a negative correlation between SDI and ASDR, consistent with previous research findings (25), indicating that health education, public health policies, medical resources, and living conditions in economically developed regions contribute to a lower incidence of laryngeal cancer.

Despite the decline in age-standardized incidence rates, the absolute numbers of prevalent cases, incidence cases, deaths, and disability caused by laryngeal cancer increased from 1990 to 2021. This growth is mainly due to population growth and aging, as the incidence of age-related cancers is expected to continue increasing. Laryngeal cancer is particularly notable in this regard, with statistics showing that it predominantly affects the middle-aged and elderly populations, primarily impacting the vocal cords. A survey based on U.S. population data revealed that from 2000 to 2019, over 100,000 cases of laryngeal cancer were recorded in the United States. Among all racial/ethnic groups, cases of laryngeal cancer were rarely reported before the age of 30-34. After that, both male and female incidence rates of laryngeal cancer increased significantly, with males peaking at ages 60–64 and females peaking at ages 65-69 (26).

Smoking and alcohol consumption remain major risk factors for laryngeal cancer. Smoking directly damages the cells of the respiratory tract and larynx (27). A meta-analysis has shown that smokers are four times more likely to develop laryngeal cancer compared to non-smokers. Additionally, frequent alcohol consumption is another significant risk factor for laryngeal cancer, as excessive drinking increases the risk of upper digestive tract cancers, including laryngeal cancer (28, 29). Studies have further confirmed a dose-response relationship between smoking and the risk of laryngeal cancer. When combined with heavy alcohol consumption, the risk of laryngeal cancer increases significantly, with individuals who both smoke and drink being 177 times more likely to develop the disease compared to those who neither smoke nor drink (30).This synergistic effect of tobacco and alcohol substantially amplifies the risk of laryngeal cancer (31).

In addition to the well-documented effects of smoking and alcohol consumption, occupational exposure is also a significant risk factor for laryngeal cancer. This study identified exposure to asbestos and strong acids as the third and fourth leading risk factors for laryngeal cancer, following smoking and alcohol use. Long-term asbestos exposure has been associated with significantly higher incidence and mortality rates of laryngeal cancer among workers. Similarly, individuals exposed to strong acids in industrial environments face an elevated risk of laryngeal cancer due to the direct chemical damage these substances can cause to laryngeal cells (32). The mucosa of the larynx is directly exposed to inhaled carcinogens, such as asbestos and strong acids, making it a primary target for respiratory system carcinogens. Although these risk factors have been confirmed to be associated with laryngeal cancer, the specific mechanisms underlying carcinogenesis remain incompletely understood.

Gender differences are particularly significant in the burden of laryngeal cancer, with men consistently showing a higher incidence rate than women. This disparity is primarily attributed to higher rates of smoking and alcohol consumption among men. In addition, hormonal and genetic factors may also play a role. According to Hashim’s study, women who undergo menopause before the age of 52 face an elevated risk of cancer due to the loss of estrogen’s protective effects (33). Furthermore, studies have demonstrated that in laryngeal cancer cells, the expression levels of androgen, estrogen, and prolactin receptors are significantly higher compared to normal tissues. This suggests that laryngeal cancer cells are more sensitive to hormones, which influences their proliferation and biological behavior. Specifically, the binding of estrogen (E2) to estrogen receptor alpha 36 (ERα 36) significantly promotes the proliferation of laryngeal cancer cells and activates molecules such as protein kinase C (PKC) and phospholipase D. These mechanisms not only enhance the resistance of cancer cells to chemotherapy-induced apoptosis but also promote angiogenesis and tumor metastasis, supplying the tumor with necessary blood and nutrients (34–37). Additionally, Fei et al. were the first to report the co-expression of prolactin receptor (PRLR) and estrogen receptor (ER) in laryngeal cancer cells. This co-expression may contribute to tumor growth and dissemination. Notably, high expression of the PRLR receptor is closely associated with poorer prognosis, indicating that tumors may exhibit greater invasiveness and reduced sensitivity to treatment (34).These findings provide deeper insights into the differences in laryngeal cancer incidence between genders. Understanding these gender-specific disparities is critical for designing health interventions tailored to the unique needs and risk factors of different populations. Considering these findings, future interventions should address both biological and behavioral risk factors to reduce disparities in laryngeal cancer burden.

Studies have also shown that the burden of laryngeal cancer is particularly significant among middle-aged and older adults. With the global trend of population aging, this issue is likely to worsen.

To effectively reduce the global burden of laryngeal cancer, public health efforts should prioritize cost-effective, region-specific interventions. In high-income regions, focus should be placed on optimizing screening programs, such as electronic laryngoscopy and advanced diagnostic technologies, as well as implementing long-term surveillance systems for high-risk populations. In contrast, economically underdeveloped regions require expanded access to primary healthcare services, alongside targeted health education campaigns aimed at reducing smoking and alcohol consumption.

A multi-pronged approach is necessary, combining robust anti-tobacco policies, such as increased taxation and advertising restrictions, with improved workplace safety standards to minimize exposure to occupational hazards like asbestos and strong acids. Public health campaigns should leverage mass media to educate communities about modifiable risk factors, emphasizing lifestyle changes to reduce cancer risk.

Regular screenings for high-risk individuals—such as smokers, heavy drinkers, and those exposed to industrial chemicals—should be integrated into national cancer control programs. These efforts could significantly improve early detection rates and reduce both morbidity and mortality associated with laryngeal cancer. Moreover, governments and healthcare systems must allocate resources equitably to ensure accessibility of preventive and therapeutic services across all socioeconomic groups.

Finally, strengthening international collaboration and data-sharing initiatives can enhance global monitoring of laryngeal cancer trends, enabling more accurate disease forecasting and better-targeted interventions. These strategies will not only alleviate the burden of laryngeal cancer but also contribute to reducing global health inequalities.

This study has several limitations. Differences in the completeness and reliability of data across regions exist, particularly in low-income countries, which may lead to an underestimation of the actual burden of laryngeal cancer. The use of national-level aggregated data may introduce bias, potentially obscuring geographical disparities. Moreover, the GBD database lacks anatomical details of laryngeal cancer, making it difficult to analyze how tumor location affects treatment outcomes and quality of life. For instance, early-stage laryngeal cancer can be treated with laser surgery to preserve vocal function, whereas tumors located in the supraglottic or subglottic regions may require more extensive surgical resection, significantly reducing patients’ quality of life. Therefore, future studies should focus on the anatomical subdivision of laryngeal cancer to inform more effective public health strategies.

## Conclusion

This study provides a comprehensive assessment of the global burden of laryngeal cancer across 204 countries and regions. Although age-standardized prevalence, incidence, mortality, and DALY rates of laryngeal cancer have declined from 1990 to 2021, the absolute numbers of cases, deaths, and disability continue to rise, primarily driven by population growth and aging. Regions with higher incidence rates, such as Central Europe and the Caribbean, require urgent attention to address the underlying risk factors and implement targeted interventions. To mitigate the burden of laryngeal cancer, especially in economically underdeveloped regions, governments and public health organizations should prioritize cost-effective strategies. These include expanding access to early screening programs, promoting health education campaigns targeting smoking and alcohol consumption, and enforcing stricter occupational safety regulations to reduce exposure to carcinogens such as asbestos and strong acids. Finally, international collaboration and data-sharing initiatives are crucial to enhance global monitoring and forecasting of laryngeal cancer trends. By implementing region-specific interventions and fostering equitable resource allocation, it is possible to alleviate the burden of laryngeal cancer and address the persistent health disparities associated with this disease.

## Data Availability

The original contributions presented in the study are included in the article/**Supplementary Material**. Further inquiries can be directed to the corresponding authors.
