# Supplementary material for: Global, regional, and national larynx cancer burden and health inequality analysis from 1990 to 2021 with a prediction from 2022 to 2040
Source: Front Oncol. 2025 Jul 18;15:1617613. doi: 10.3389/fonc.2025.1617613 (PMC12351386; doi:10.3389/fonc.2025.1617613)
Supplement: Supplementary file 1 [file Table1.docx]

| **TableS1: Prevalence cases and ASPR of larynx cancer in 1990 and 2021and its trends** | | | | | |
| --- | --- | --- | --- | --- | --- |
| Location | Number 1990 | ASR 1990 | Number 2021 | ASR 2021 | EAPC_95%CI |
| American Samoa | 1.27 (0.99-1.6) | 5.49 (4.26-6.88) | 1.5 (1.2-1.89) | 3.08 (2.49-3.86) | -2.46 (-2.74 to -2.18) |
| Antigua and Barbuda | 4.93 (4.49-5.36) | 9.83 (8.94-10.68) | 11.64 (10.44-13.09) | 10.31 (9.24-11.61) | 0.22 (-0.05 to 0.48) |
| Arab Republic of Egypt | 1666.09 (1427.99-2065.93) | 5.74 (4.92-7.13) | 5238.96 (4124.62-6625.58) | 7.67 (6.12-9.54) | 1.1 (0.96 to 1.25) |
| Argentine Republic | 7135.46 (6517.98-7790.46) | 21.78 (19.88-23.76) | 7435.66 (6761.63-8177.39) | 13.54 (12.3-14.89) | -1.7 (-1.93 to -1.46) |
| Australia | 2010.98 (1811.73-2233.62) | 10.52 (9.45-11.68) | 2856 (2545.63-3152.83) | 6.9 (6.09-7.64) | -1.31 (-1.46 to -1.16) |
| Barbados | 22.18 (20.42-24.15) | 8.27 (7.64-8.96) | 51.79 (40.67-65.42) | 10.01 (7.84-12.71) | 0.67 (0.51 to 0.83) |
| Belize | 5.9 (5.48-6.35) | 6.36 (5.91-6.85) | 26.31 (22.82-30.72) | 8.35 (7.28-9.76) | 1.12 (0.69 to 1.55) |
| Bermuda | 9.9 (8.95-10.93) | 15.54 (14.07-17.13) | 21.4 (17.74-26.62) | 16.69 (13.7-20.73) | 0.51 (0.31 to 0.72) |
| Bolivarian Republic of Venezuela | 1243.45 (1180.44-1305.73) | 12.66 (11.99-13.3) | 3494.58 (2662.05-4544.5) | 11.25 (8.6-14.57) | -0.89 (-1.1 to -0.69) |
| Bosnia and Herzegovina | 1093.49 (929.67-1274.98) | 23.69 (20.38-27.33) | 1405.48 (1033.47-1782.28) | 23.35 (17.06-29.75) | 0.09 (-0.01 to 0.19) |
| Brunei Darussalam | 14.31 (11.66-17.56) | 14.17 (11.51-17.37) | 30.19 (23.93-38.19) | 8.37 (6.72-10.48) | -1.84 (-1.95 to -1.73) |
| Burkina Faso | 174.72 (130.8-224.63) | 3.84 (2.93-4.9) | 457.62 (320.77-616.23) | 4.75 (3.39-6.32) | 1.04 (0.88 to 1.2) |
| Canada | 7700.02 (6991.84-8402.57) | 24.25 (22.08-26.41) | 9128.26 (8255.89-10099.58) | 13.16 (11.87-14.62) | -1.88 (-2.07 to -1.69) |
| Central African Republic | 97.76 (58.77-134.82) | 7.64 (4.85-10.31) | 162.46 (95.73-237.24) | 6.32 (4.08-8.88) | -0.65 (-0.72 to -0.59) |
| Commonwealth of Dominica | 6.03 (5.15-7.13) | 10.32 (8.79-12.19) | 10.85 (8.31-14.12) | 12.35 (9.5-15.97) | 0.67 (0.62 to 0.72) |
| Commonwealth of the Bahamas | 22.52 (20.39-24.76) | 14.01 (12.71-15.35) | 64.18 (51.11-80.11) | 14.55 (11.6-18.1) | 0.43 (0.3 to 0.57) |
| Cook Islands | 0.38 (0.3-0.47) | 3.07 (2.42-3.77) | 0.8 (0.62-1.07) | 3.05 (2.38-4.11) | -0.17 (-0.53 to 0.21) |
| Czech Republic | 2443.57 (2120.39-2807.13) | 18.45 (16.03-21.16) | 2895.76 (2349.66-3533.67) | 15.14 (12.26-18.5) | -0.57 (-0.78 to -0.37) |
| Democratic People's Republic of Korea | 998.24 (716.77-1343.02) | 5.6 (4.11-7.4) | 2210.84 (1555.4-2917.86) | 6.4 (4.54-8.44) | 0.6 (0.49 to 0.71) |
| Democratic Republic of Sao Tome and Principe | 1.96 (1.56-2.41) | 2.94 (2.35-3.6) | 4.28 (3.48-5.42) | 3.77 (3.09-4.75) | 0.89 (0.82 to 0.95) |
| Democratic Republic of Timor-Leste | 13.06 (8.95-18.63) | 4.42 (3.11-6.11) | 39.65 (28.05-55.47) | 4.51 (3.21-6.28) | 0.27 (0.03 to 0.51) |
| Democratic Republic of the Congo | 972.51 (678.63-1307.54) | 5.83 (4.2-7.69) | 2265.1 (1603.38-3067.08) | 5.58 (3.99-7.43) | -0.13 (-0.33 to 0.08) |
| Democratic Socialist Republic of Sri Lanka | 560.89 (451.21-699.35) | 5.02 (4.08-6.25) | 2426.44 (1454.11-3662.36) | 8.6 (5.22-12.95) | 2.88 (2.44 to 3.31) |
| Dominican Republic | 281.32 (226.37-358.18) | 7.42 (5.98-9.37) | 848.88 (615.8-1134.38) | 8.37 (6.08-11.18) | 0.59 (0.47 to 0.71) |
| Eastern Republic of Uruguay | 1298.59 (1195.75-1416.1) | 34.08 (31.38-37.14) | 1188.2 (1078.66-1318.37) | 23.65 (21.38-26.26) | -1.31 (-1.42 to -1.2) |
| Federal Democratic Republic of Ethiopia | 1258.08 (722.96-1785.98) | 5.74 (3.44-8.1) | 1899.43 (1388.85-2542.34) | 4.12 (3.04-5.47) | -1.38 (-1.53 to -1.22) |
| Federal Democratic Republic of Nepal | 1219.2 (842.92-1709.4) | 12.02 (8.46-16.52) | 2635.4 (1952.23-3621.3) | 10.79 (8.03-14.7) | -0.37 (-0.61 to -0.12) |
| Federal Republic of Germany | 20325.3 (18638-22286.97) | 17.49 (15.99-19.17) | 25167.08 (22931.56-27615.42) | 14.64 (13.31-16.14) | -0.87 (-1.08 to -0.67) |
| Federal Republic of Nigeria | 2650.2 (1897.64-3544.31) | 5.66 (4.1-7.51) | 4839.38 (3313.5-6625.24) | 4.94 (3.51-6.63) | -0.42 (-0.53 to -0.32) |
| Federal Republic of Somalia | 231.15 (150.96-336.67) | 8.16 (5.5-11.48) | 463.56 (292.99-670.35) | 6.49 (4.23-9.12) | -0.8 (-0.85 to -0.75) |
| Federated States of Micronesia | 1.65 (1.26-2.2) | 3.31 (2.54-4.38) | 2.52 (1.83-3.43) | 3.17 (2.35-4.28) | -0.22 (-0.26 to -0.17) |
| Federative Republic of Brazil | 13815.1 (13238.09-14359.33) | 14.42 (13.81-14.99) | 38510.96 (36140.67-40806.33) | 14.89 (13.96-15.77) | 0.05 (-0.04 to 0.15) |
| French Republic | 37813.38 (34311.77-41572.71) | 51.53 (46.72-56.62) | 39252.28 (34934.76-44109.69) | 33.62 (29.92-37.59) | -1.07 (-1.33 to -0.82) |
| Gabonese Republic | 47.93 (35.91-62.86) | 8.09 (6.09-10.55) | 88.74 (63.61-117.96) | 7.7 (5.6-10.14) | -0.21 (-0.26 to -0.16) |
| Georgia | 1554.93 (1436.3-1685.97) | 23.72 (21.8-25.71) | 1138.97 (994.57-1305.61) | 19.77 (17.25-22.6) | -0.34 (-0.6 to -0.07) |
| Grand Duchy of Luxembourg | 157.9 (144.35-172.72) | 30.15 (27.56-32.94) | 197.19 (175.64-219.8) | 19.38 (17.24-21.64) | -1.34 (-1.49 to -1.19) |
| Greenland | 6.2 (5.03-7.55) | 15.62 (12.72-18.73) | 7.96 (6.4-10.02) | 10.26 (8.29-12.89) | -0.95 (-1.15 to -0.76) |
| Grenada | 5.15 (4.6-5.78) | 7.73 (6.86-8.69) | 8.56 (7.34-9.89) | 6.93 (5.96-8.01) | 0.03 (-0.38 to 0.45) |
| Guam | 2.91 (2.47-3.36) | 3.62 (3.07-4.16) | 4.34 (3.75-5.05) | 2.09 (1.81-2.42) | -1.73 (-2.14 to -1.31) |
| Hashemite Kingdom of Jordan | 131.97 (100.38-166.49) | 9.08 (6.95-11.47) | 586.4 (422.33-805.2) | 7.14 (5.18-9.77) | -1.12 (-1.38 to -0.86) |
| Hellenic Republic | 5442.56 (5060.32-5812.09) | 35.92 (33.5-38.39) | 5995.72 (5483.91-6468.75) | 29.53 (26.91-31.9) | -0.62 (-0.73 to -0.51) |
| Hungary | 4238.86 (3807.43-4686.82) | 30.11 (26.97-33.34) | 5238.8 (4416.02-6181.19) | 31.28 (26.02-37.43) | -0.36 (-0.6 to -0.11) |
| Independent State of Papua New Guinea | 41.17 (27.37-58.56) | 2.27 (1.55-3.17) | 108.68 (76.73-156.81) | 2.15 (1.54-3.04) | -0.26 (-0.33 to -0.18) |
| Independent State of Samoa | 1.31 (1-1.71) | 1.46 (1.14-1.89) | 2.31 (1.79-3.09) | 1.52 (1.19-2) | 0 (-0.09 to 0.09) |
| Ireland | 816.77 (739.72-904.58) | 20.88 (18.89-23.18) | 1104.41 (971.71-1258.38) | 14.61 (12.76-16.69) | -0.62 (-0.79 to -0.44) |
| Islamic Republic of Afghanistan | 923.36 (506.69-1434.8) | 12.72 (7.2-19.37) | 1345.71 (749.94-2082.11) | 12.27 (7.29-17.88) | -0.23 (-0.29 to -0.17) |
| Islamic Republic of Iran | 4206.14 (3564.14-4785.74) | 14.91 (12.65-16.93) | 14141.2 (12651.53-16089.09) | 17.48 (15.72-19.78) | 0.54 (0.42 to 0.67) |
| Islamic Republic of Mauritania | 38.5 (30.45-48.9) | 3.79 (3.02-4.76) | 103.94 (72.74-147.38) | 4.68 (3.3-6.59) | 0.81 (0.59 to 1.03) |
| Islamic Republic of Pakistan | 12461.21 (10191.89-15173.3) | 21.28 (17.44-25.87) | 28711.38 (21568.44-37815.21) | 21.43 (16.16-27.97) | -0.25 (-0.42 to -0.09) |
| Jamaica | 114.23 (102.3-126.72) | 6.63 (5.92-7.4) | 248.08 (182.27-333.33) | 8.07 (5.94-10.84) | 0.65 (0.24 to 1.07) |
| Japan | 20747.28 (19654.71-21972.74) | 11.92 (11.28-12.63) | 25321.88 (23271.01-27057.47) | 7.42 (6.9-7.85) | -1.55 (-1.8 to -1.3) |
| Kingdom of Bahrain | 29.99 (24.44-38.13) | 16.17 (13.33-20.4) | 138.24 (101.79-196.88) | 13.73 (10.29-19.01) | -1.09 (-1.29 to -0.9) |
| Kingdom of Belgium | 5038.29 (4591.83-5491.07) | 35.43 (32.28-38.62) | 4108.45 (3727.28-4566.75) | 19.87 (17.97-22.01) | -2 (-2.13 to -1.87) |
| Kingdom of Bhutan | 28.09 (18.6-40.33) | 10.48 (7.08-14.86) | 61.42 (43.2-84.48) | 9.9 (7.01-13.53) | -0.22 (-0.35 to -0.08) |
| Kingdom of Cambodia | 342.57 (253.54-453.16) | 7.22 (5.51-9.54) | 1004 (707.37-1493.61) | 7.61 (5.42-11.28) | 0.1 (-0.07 to 0.28) |
| Kingdom of Denmark | 1760.23 (1605.82-1912.24) | 24.22 (22.02-26.34) | 1825.16 (1662.23-1987.91) | 16.79 (15.22-18.34) | -1.15 (-1.26 to -1.05) |
| Kingdom of Eswatini | 38.69 (25.87-52.92) | 12.23 (8.15-16.45) | 75.49 (46.2-109.07) | 11.84 (7.42-16.93) | 0.03 (-0.19 to 0.24) |
| Kingdom of Lesotho | 76.69 (59.04-97.54) | 8.7 (6.72-11.03) | 150.76 (103.45-204.56) | 12.73 (8.8-17.13) | 1.59 (1.39 to 1.78) |
| Kingdom of Morocco | 1414.4 (1096.12-1741.73) | 9.69 (7.58-11.89) | 4279.82 (3020.93-5666.84) | 11.79 (8.34-15.47) | 0.71 (0.51 to 0.92) |
| Kingdom of Norway | 716.65 (658.94-779.26) | 11.57 (10.62-12.61) | 885.72 (803.06-975.56) | 9.17 (8.36-10.12) | -0.85 (-1.13 to -0.56) |
| Kingdom of Saudi Arabia | 304.76 (227.61-397.5) | 4.96 (3.75-6.37) | 1500.02 (1147.24-1992.87) | 6.34 (5.03-8.14) | 0.7 (0.63 to 0.78) |
| Kingdom of Spain | 22999.87 (20192.78-25834.66) | 44.79 (39.29-50.33) | 24225.86 (21147.19-27927.94) | 28.31 (24.56-32.92) | -1.69 (-1.9 to -1.49) |
| Kingdom of Sweden | 1208.68 (1081.69-1335.37) | 8.61 (7.73-9.58) | 1361.23 (1188.68-1544.58) | 6.73 (5.85-7.69) | -0.41 (-0.63 to -0.19) |
| Kingdom of Thailand | 4418.66 (3627.76-5309.94) | 11.71 (9.66-13.98) | 14089.66 (10668.05-18388.64) | 12.83 (9.73-16.65) | 0.04 (-0.06 to 0.15) |
| Kingdom of Tonga | 1.53 (1.12-2.11) | 2.76 (2.04-3.77) | 2.27 (1.64-3.11) | 2.83 (2.04-3.87) | -0.04 (-0.19 to 0.11) |
| Kingdom of the Netherlands | 4324.62 (3980.26-4710.38) | 22.81 (20.94-24.82) | 4904.32 (4472.02-5362.37) | 14.64 (13.31-16.06) | -1.45 (-1.56 to -1.34) |
| Kyrgyz Republic | 328.42 (279.35-385.59) | 10.36 (8.8-12.18) | 267.13 (207.62-335.13) | 4.94 (3.86-6.15) | -2.42 (-2.57 to -2.26) |
| Lao People's Democratic Republic | 148.54 (103.65-205.71) | 6.73 (4.77-9.25) | 264.18 (182.96-381.99) | 5.45 (3.84-7.84) | -0.69 (-0.82 to -0.56) |
| Lebanese Republic | 417.61 (285.13-575.78) | 18.07 (12.57-24.37) | 1229.47 (989.87-1540.22) | 21.06 (16.78-26.42) | 0.89 (0.69 to 1.08) |
| Malaysia | 780.77 (607.53-941.53) | 8.26 (6.37-9.94) | 2789.57 (2209.56-3310.46) | 9.43 (7.44-11.13) | 0.35 (0.14 to 0.55) |
| Mongolia | 70.23 (51.99-90.53) | 6.35 (4.75-8.1) | 119.7 (87.63-161.58) | 4.44 (3.35-6) | -1.33 (-1.48 to -1.19) |
| Montenegro | 301.78 (243.22-377.66) | 45.52 (36.74-56.77) | 482.17 (377.79-629.65) | 49.48 (38.74-64.64) | 0.47 (0.33 to 0.61) |
| New Zealand | 632.6 (566.36-701.29) | 16.25 (14.55-18.04) | 841.89 (734.39-959.58) | 10.07 (8.8-11.43) | -1.61 (-1.81 to -1.4) |
| North Macedonia | 505.88 (423.18-619.17) | 25.33 (21.19-31.05) | 947.92 (726.45-1224.91) | 27.52 (21.18-35.35) | 0.41 (0.17 to 0.66) |
| Northern Mariana Islands | 1.32 (0.94-1.79) | 7.04 (5.25-9.03) | 2.56 (2.1-3.03) | 4.78 (3.99-5.66) | -1.99 (-2.38 to -1.59) |
| Palestine | 66.43 (50.7-85.39) | 7.57 (5.79-9.7) | 200.71 (162.9-246.83) | 7.43 (6.07-9.08) | -0.34 (-0.46 to -0.22) |
| People's Democratic Republic of Algeria | 1252.48 (978.63-1616.45) | 10.04 (7.95-12.81) | 3756.46 (2850.9-4907.4) | 10.15 (7.79-13.22) | 0.01 (-0.16 to 0.19) |
| People's Republic of Bangladesh | 7870.26 (6183.8-9814.91) | 16.06 (12.68-19.96) | 17576.79 (12854.61-23628.75) | 12.25 (8.99-16.36) | -0.93 (-1.02 to -0.85) |
| People's Republic of China | 69308.82 (56887.4-81339.87) | 7.83 (6.47-9.13) | 217849.05 (171468.97-273850.85) | 9.86 (7.81-12.35) | 0.9 (0.74 to 1.06) |
| Plurinational State of Bolivia | 196.14 (143.83-256.09) | 5.95 (4.35-7.75) | 442.48 (308.96-622.87) | 4.73 (3.33-6.64) | -0.75 (-0.85 to -0.64) |
| Portuguese Republic | 2572.37 (2352.7-2830.17) | 18.95 (17.32-20.77) | 2946.9 (2666.57-3272.69) | 14.26 (12.87-15.89) | -0.87 (-1.1 to -0.64) |
| Principality of Andorra | 12.41 (8.63-18.74) | 21.04 (14.69-31.57) | 23.36 (15.29-33.04) | 15.26 (9.97-21.58) | -0.9 (-1.14 to -0.66) |
| Principality of Monaco | 48.77 (35.75-64.64) | 84.77 (61.56-112.94) | 55.77 (43.04-73.48) | 69.02 (52.52-93.39) | -0.72 (-0.79 to -0.64) |
| Puerto Rico | 533.16 (473.62-591.83) | 14.74 (13.09-16.37) | 568.87 (458.06-692.47) | 9.23 (7.35-11.37) | -1.23 (-1.56 to -0.9) |
| Republic of Albania | 377.03 (311.96-454.12) | 17.23 (14.24-20.81) | 752.27 (545.42-1039.23) | 17.62 (13.01-24.32) | 0.55 (0.39 to 0.7) |
| Republic of Angola | 307.67 (210.66-426.35) | 7.16 (5.02-9.75) | 848.99 (641.52-1107.19) | 6.49 (5.05-8.28) | -0.28 (-0.43 to -0.12) |
| Republic of Armenia | 693.36 (658.14-725.22) | 22.83 (21.74-23.83) | 569.1 (508.03-635.31) | 12.98 (11.61-14.48) | -1.83 (-2.06 to -1.6) |
| Republic of Austria | 1984.91 (1803.22-2183.29) | 18.67 (16.88-20.59) | 2060.47 (1836.97-2273.38) | 12.57 (11.2-13.9) | -1.26 (-1.4 to -1.11) |
| Republic of Azerbaijan | 832.81 (728.33-956.24) | 15.05 (13.09-17.43) | 1281.01 (963.8-1793.38) | 10.85 (8.24-14.94) | -1.11 (-1.23 to -0.98) |
| Republic of Belarus | 3082.47 (2710.86-3521.76) | 23.34 (20.49-26.6) | 3581.11 (2768.92-4581.98) | 22.8 (17.45-29.25) | -1.09 (-1.43 to -0.76) |
| Republic of Benin | 76.81 (61.24-93.94) | 3.83 (3.06-4.67) | 232 (170.38-303.49) | 4.31 (3.18-5.58) | 0.6 (0.48 to 0.72) |
| Republic of Botswana | 66.51 (49.29-84.23) | 10.85 (8.12-13.6) | 132.31 (94.5-204.17) | 8.11 (5.87-12.14) | -1.14 (-1.28 to -1.01) |
| Republic of Bulgaria | 2818.71 (2418.23-3255.23) | 22.71 (19.48-26.34) | 3861.41 (3133.98-4748.67) | 31.01 (25.08-38.32) | 1.22 (1.01 to 1.43) |
| Republic of Burundi | 209.9 (135.53-290.49) | 8.5 (5.57-11.68) | 324.35 (228.97-459.95) | 5.95 (4.29-8.24) | -1.38 (-1.58 to -1.18) |
| Republic of Cabo Verde | 15.86 (12.5-19.58) | 6.97 (5.51-8.62) | 36.95 (25.61-48.41) | 7.9 (5.5-10.23) | 0.28 (-0.18 to 0.75) |
| Republic of Cameroon | 201.97 (160.12-262) | 4.26 (3.38-5.43) | 738.32 (491.51-1044.61) | 5.45 (3.7-7.62) | 1.01 (0.9 to 1.12) |
| Republic of Chad | 90.69 (69.2-113.99) | 3.19 (2.45-3.99) | 317.12 (222.76-421.91) | 5.26 (3.76-6.93) | 1.92 (1.79 to 2.04) |
| Republic of Chile | 1026.21 (936.9-1133.34) | 9.98 (9.12-11.03) | 1801 (1605.66-1991) | 7.03 (6.27-7.77) | -1 (-1.11 to -0.9) |
| Republic of Colombia | 2083.25 (1915.5-2243.76) | 11.55 (10.63-12.46) | 3318.83 (2681.55-4076.56) | 6.01 (4.86-7.37) | -2.73 (-2.93 to -2.53) |
| Republic of Costa Rica | 182.69 (166.21-201.24) | 10.42 (9.47-11.47) | 307.77 (265.42-361.22) | 5.55 (4.79-6.52) | -2.16 (-2.35 to -1.96) |
| Republic of Croatia | 2166.46 (1964.35-2395.33) | 33.14 (30.01-36.53) | 1922.39 (1617.86-2225.58) | 24.41 (20.54-28.42) | -1 (-1.19 to -0.82) |
| Republic of Cuba | 2777.21 (2527.99-3071.84) | 27.2 (24.75-30.05) | 7932.66 (6563.73-9588.24) | 40.96 (34.04-49.37) | 1.49 (1.34 to 1.64) |
| Republic of Cyprus | 109.44 (89.97-133.36) | 13.37 (11-16.23) | 294.59 (237.31-366.27) | 14.49 (11.69-18.02) | 0.78 (0.57 to 0.99) |
| Republic of C么te d'Ivoire | 235.31 (184.56-300.71) | 5.26 (4.22-6.6) | 653.59 (455.82-990.68) | 5.2 (3.73-7.72) | -0.22 (-0.31 to -0.12) |
| Republic of Djibouti | 13.53 (9.27-20.28) | 8.43 (5.93-12.32) | 59.23 (39.92-85.1) | 7.94 (5.5-11.07) | -0.31 (-0.37 to -0.25) |
| Republic of Ecuador | 241.15 (223.98-261.79) | 4.48 (4.14-4.86) | 514.52 (395.73-662.27) | 3.12 (2.4-4) | -1.3 (-1.56 to -1.04) |
| Republic of El Salvador | 123.97 (107.13-143.69) | 4.16 (3.59-4.83) | 255.9 (198.83-328.84) | 4.23 (3.27-5.45) | -0.28 (-0.47 to -0.08) |
| Republic of Equatorial Guinea | 14.85 (8.93-21.45) | 7.01 (4.37-9.95) | 33.82 (20.39-50.23) | 6.12 (3.77-8.89) | -0.48 (-0.8 to -0.17) |
| Republic of Estonia | 470.21 (410.82-534.87) | 22.98 (20.14-26.04) | 476.56 (395.78-584.7) | 20.73 (17.04-25.64) | -0.66 (-0.91 to -0.4) |
| Republic of Fiji | 12.95 (10.33-16.18) | 3.52 (2.85-4.35) | 27.34 (20.45-35.83) | 3.41 (2.59-4.42) | -0.02 (-0.16 to 0.11) |
| Republic of Finland | 870.21 (797.76-951.93) | 12.87 (11.74-14.09) | 1184.57 (1056.92-1320.94) | 10.97 (9.84-12.28) | -0.49 (-0.66 to -0.33) |
| Republic of Ghana | 226.24 (175.61-295.8) | 3.43 (2.71-4.4) | 883.07 (631.53-1144.6) | 4.93 (3.59-6.31) | 1.7 (1.51 to 1.89) |
| Republic of Guatemala | 209.91 (200.36-220.39) | 5.97 (5.69-6.26) | 278.25 (238.1-325.85) | 2.5 (2.15-2.93) | -2.98 (-3.18 to -2.78) |
| Republic of Guinea | 113.14 (87.1-144.12) | 3.35 (2.58-4.25) | 291.69 (215.16-377.3) | 5.03 (3.77-6.46) | 1.77 (1.61 to 1.92) |
| Republic of Guinea-Bissau | 21.55 (13.63-29.99) | 5.14 (3.32-7.08) | 47.95 (33.19-66.3) | 6.04 (4.19-8.2) | 0.92 (0.77 to 1.07) |
| Republic of Guyana | 19.1 (16.54-22.32) | 4.86 (4.23-5.67) | 36.06 (26.84-47.69) | 5.13 (3.87-6.73) | 0.63 (0.42 to 0.84) |
| Republic of Haiti | 352.44 (237.14-468.48) | 10.24 (7-13.46) | 677.59 (443.97-950.46) | 8.89 (5.82-12.34) | -0.33 (-0.48 to -0.19) |
| Republic of Honduras | 100.66 (82.37-125.09) | 4.82 (3.95-6) | 407.76 (314.02-527.86) | 6.32 (4.81-8.13) | 1.05 (0.96 to 1.15) |
| Republic of Iceland | 34.95 (31.29-39.07) | 12.9 (11.54-14.49) | 48.06 (42.01-54.55) | 8.89 (7.76-10.13) | -1.41 (-1.55 to -1.27) |
| Republic of India | 66439.18 (56168.63-78915.06) | 12.95 (10.93-15.35) | 162834.03 (142268.54-186821.61) | 12.92 (11.32-14.8) | -0.16 (-0.35 to 0.03) |
| Republic of Indonesia | 5277.28 (4078.19-6315.12) | 5.08 (3.93-6.09) | 14599.95 (10643.22-19216.75) | 5.66 (4.13-7.36) | 0.32 (0.27 to 0.36) |
| Republic of Iraq | 1114.6 (876.89-1411.42) | 13.66 (10.73-17.19) | 4200.15 (2997.37-5568.38) | 16.35 (11.75-21.41) | 0.38 (0.25 to 0.51) |
| Republic of Italy | 29406.1 (27508.81-31263.61) | 34.32 (32.11-36.49) | 25579.25 (23692.45-27653.44) | 20.14 (18.72-21.72) | -1.78 (-1.92 to -1.65) |
| Republic of Kazakhstan | 2376.87 (2197.98-2562.28) | 17.26 (15.99-18.62) | 1696.24 (1476.13-1923.07) | 8.52 (7.43-9.62) | -2.75 (-2.9 to -2.59) |
| Republic of Kenya | 462.4 (342.81-617.97) | 5.29 (3.94-7.03) | 1595.7 (1207.21-2062.18) | 6.19 (4.7-7.91) | 0.58 (0.47 to 0.69) |
| Republic of Kiribati | 0.31 (0.25-0.41) | 0.82 (0.65-1.07) | 0.69 (0.5-0.99) | 0.89 (0.66-1.26) | 0.23 (0.15 to 0.31) |
| Republic of Korea | 6804.54 (4822.9-8616.95) | 21.32 (15.28-26.84) | 14029.54 (10669.61-17835.53) | 14.62 (11.15-18.56) | -1.85 (-2.16 to -1.55) |
| Republic of Latvia | 711.82 (635.07-794.84) | 19.99 (17.78-22.44) | 526.51 (448.07-615.23) | 15.71 (13.12-18.6) | -1.08 (-1.27 to -0.88) |
| Republic of Liberia | 42.01 (30.81-59.91) | 3.61 (2.67-5.14) | 103.46 (70.78-148.11) | 4.63 (3.25-6.49) | 1 (0.83 to 1.16) |
| Republic of Lithuania | 1007.09 (881.44-1155.46) | 22.3 (19.54-25.57) | 930.53 (747.51-1129.48) | 18.55 (14.83-22.62) | -0.89 (-1.08 to -0.69) |
| Republic of Madagascar | 361.68 (268.75-447.56) | 6.62 (5.01-8.21) | 621.68 (452.91-834.81) | 4.75 (3.49-6.3) | -1.13 (-1.26 to -1) |
| Republic of Malawi | 97.95 (77.01-129.07) | 2.35 (1.86-3.09) | 215.13 (162.32-299.98) | 2.59 (1.98-3.56) | 0.18 (0.07 to 0.28) |
| Republic of Maldives | 5.08 (3.55-6.55) | 5.57 (4.13-7) | 13.89 (10.32-18.15) | 3.95 (3-5.08) | -1.31 (-1.43 to -1.18) |
| Republic of Mali | 184.19 (153.08-224.32) | 4.29 (3.6-5.14) | 404.7 (302.05-551) | 4.25 (3.22-5.72) | 0.18 (0.05 to 0.32) |
| Republic of Malta | 94.03 (81.75-107.18) | 21.89 (19.03-24.96) | 129.6 (111.01-151.96) | 15.53 (13.14-18.43) | -1.16 (-1.24 to -1.07) |
| Republic of Mauritius | 94.67 (88.3-102.9) | 12.25 (11.45-13.28) | 228.05 (207.72-246.24) | 11.85 (10.82-12.76) | 0.03 (-0.62 to 0.69) |
| Republic of Moldova | 930.92 (873.58-988.29) | 19.92 (18.72-21.11) | 1192.55 (1060.83-1353.71) | 20.07 (17.86-22.73) | -0.06 (-0.37 to 0.26) |
| Republic of Mozambique | 479.63 (343.57-631.97) | 7.33 (5.43-9.61) | 957.75 (707.93-1252.34) | 7.54 (5.6-9.68) | 0.38 (0.28 to 0.48) |
| Republic of Namibia | 77.39 (63.13-94.65) | 10.97 (9.02-13.35) | 192.98 (138.43-262.23) | 12.53 (9.19-16.81) | 0.31 (0.15 to 0.48) |
| Republic of Nauru | 0.2 (0.13-0.27) | 4.37 (2.95-5.78) | 0.22 (0.14-0.31) | 3.6 (2.43-4.93) | -0.77 (-0.88 to -0.66) |
| Republic of Nicaragua | 84.81 (68.98-102.89) | 5.49 (4.45-6.65) | 222.63 (173.77-287.91) | 4.48 (3.49-5.78) | -0.68 (-0.81 to -0.55) |
| Republic of Niue | 0.06 (0.04-0.08) | 2.73 (2.01-3.55) | 0.07 (0.05-0.09) | 2.95 (2.24-3.86) | 0.02 (-0.1 to 0.15) |
| Republic of Palau | 0.29 (0.22-0.38) | 2.92 (2.16-3.8) | 0.67 (0.49-0.86) | 2.77 (2.04-3.56) | -0.3 (-0.41 to -0.18) |
| Republic of Panama | 118.42 (109.99-125.88) | 8.01 (7.44-8.51) | 214.67 (167.24-260.52) | 4.88 (3.8-5.93) | -1.97 (-2.16 to -1.78) |
| Republic of Paraguay | 155.51 (120.88-200.21) | 6.84 (5.33-8.75) | 626.59 (435.68-862.41) | 10.36 (7.24-14.19) | 1.43 (1.36 to 1.51) |
| Republic of Peru | 589.35 (476.26-715.85) | 4.89 (3.95-5.93) | 1260.77 (910.4-1755.75) | 3.76 (2.71-5.24) | -1.25 (-1.65 to -0.85) |
| Republic of Poland | 10486.66 (10030.76-10982.86) | 24.07 (23.02-25.22) | 14254.99 (12850.57-15575.83) | 21.17 (19.1-23.17) | -0.62 (-0.85 to -0.38) |
| Republic of Rwanda | 306.07 (219.24-393.34) | 9.83 (7.19-12.6) | 467.45 (321.25-673.07) | 6.68 (4.65-9.53) | -1.81 (-2.15 to -1.48) |
| Republic of San Marino | 11.87 (9.27-14.83) | 36.05 (28.06-45.19) | 12.29 (8.16-17.54) | 18.79 (12.08-27.12) | -1.51 (-1.82 to -1.19) |
| Republic of Senegal | 139.71 (111.71-168.47) | 4.17 (3.35-5.01) | 409.44 (305.76-533.3) | 5.05 (3.73-6.54) | 0.9 (0.72 to 1.08) |
| Republic of Serbia | 3423.52 (2522.66-4749.69) | 27.78 (20.55-38.53) | 4882.61 (3471.76-6549.79) | 32.47 (22.97-43.81) | 0.49 (0.28 to 0.7) |
| Republic of Seychelles | 15.72 (13.43-18.5) | 28.09 (23.86-33.07) | 36.2 (29.72-43.99) | 29.01 (23.82-34.94) | 0.76 (0.51 to 1) |
| Republic of Sierra Leone | 80.7 (61.45-104.59) | 3.91 (3-5.06) | 183.05 (129.56-254.66) | 4.65 (3.36-6.44) | 0.96 (0.77 to 1.15) |
| Republic of Singapore | 449.24 (401.77-500.09) | 19.68 (17.69-21.9) | 988.11 (859.66-1134.28) | 11.26 (9.84-12.87) | -2.19 (-2.53 to -1.86) |
| Republic of Slovenia | 570.59 (516.71-634.86) | 23.03 (20.89-25.62) | 789.24 (667.01-928.24) | 20.05 (16.86-23.74) | -0.39 (-0.56 to -0.21) |
| Republic of South Africa | 2213.18 (1818.99-2937.55) | 10.05 (8.24-13.4) | 4483.75 (3940.54-5027.9) | 8.97 (7.88-10.04) | -0.63 (-0.79 to -0.47) |
| Republic of South Sudan | 214.91 (151.83-286.72) | 8 (5.68-10.68) | 292.13 (192.09-423.31) | 6.64 (4.46-9.44) | -0.83 (-1 to -0.65) |
| Republic of Sudan | 1010.38 (640.2-1552.52) | 10.43 (6.69-15.94) | 2223.52 (1373.07-3330.66) | 10.64 (6.8-15.64) | -0.06 (-0.14 to 0.02) |
| Republic of Suriname | 10.81 (9.02-12.8) | 4.08 (3.4-4.82) | 27.52 (19.85-37.73) | 4.13 (2.97-5.65) | 0.24 (0.06 to 0.42) |
| Republic of Tajikistan | 229.82 (178.43-287.8) | 7.86 (6.12-9.82) | 311.49 (221.42-424.26) | 4.62 (3.36-6.24) | -1.91 (-2.06 to -1.75) |
| Republic of Trinidad and Tobago | 58.31 (54.3-62.53) | 6.87 (6.39-7.37) | 142.37 (107.54-184.83) | 7.19 (5.43-9.31) | 0.17 (0.05 to 0.29) |
| Republic of Tunisia | 858.92 (661.89-1102.34) | 16.39 (12.72-20.88) | 2891.46 (2042.16-4076.1) | 20.8 (14.72-29.18) | 0.61 (0.55 to 0.67) |
| Republic of Turkey | 6862.74 (5062.21-9147.95) | 18.71 (13.97-24.87) | 17857.8 (13866.72-22941.34) | 18.41 (14.32-23.57) | -0.18 (-0.33 to -0.04) |
| Republic of Uganda | 609.18 (457-775.75) | 8.85 (6.7-11.29) | 1323.98 (911.67-1861.99) | 8.03 (5.71-11.09) | -0.9 (-1.11 to -0.7) |
| Republic of Uzbekistan | 1369.57 (1196.56-1561.25) | 11.21 (9.87-12.69) | 1151.21 (922.26-1428.21) | 3.92 (3.18-4.79) | -3.13 (-3.65 to -2.62) |
| Republic of Vanuatu | 1.5 (1.01-2.15) | 2.39 (1.67-3.27) | 3.67 (2.61-5.06) | 2.06 (1.49-2.78) | -0.67 (-0.75 to -0.59) |
| Republic of Yemen | 637.28 (405.92-906.86) | 11.93 (7.74-16.86) | 1879.26 (1181.57-2695.26) | 12.45 (8.02-17.61) | 0.11 (0.06 to 0.16) |
| Republic of Zambia | 257.38 (192.99-325.53) | 8.3 (6.21-10.41) | 759.26 (374.79-1760.09) | 9.34 (4.89-20.56) | 0.3 (0.14 to 0.45) |
| Republic of Zimbabwe | 413.01 (320.19-517.68) | 9.49 (7.4-11.84) | 787.18 (592.67-1058.39) | 9.84 (7.55-12.97) | 0.17 (0 to 0.33) |
| Republic of the Congo | 90.7 (58.03-120.24) | 7.9 (5.14-10.31) | 209.13 (155.34-275.62) | 6.99 (5.29-9.09) | -0.54 (-0.7 to -0.37) |
| Republic of the Gambia | 8.15 (6.28-10.52) | 2.17 (1.68-2.8) | 22.34 (16.89-28.48) | 2.14 (1.63-2.73) | -0.19 (-0.36 to -0.01) |
| Republic of the Marshall Islands | 0.53 (0.38-0.72) | 3.13 (2.28-4.22) | 1.2 (0.81-1.68) | 3.34 (2.29-4.66) | 0.26 (0.17 to 0.35) |
| Republic of the Niger | 100.27 (73.47-130.64) | 3.4 (2.53-4.38) | 321.75 (219.82-454.12) | 3.76 (2.61-5.23) | 0.69 (0.54 to 0.85) |
| Republic of the Philippines | 1550.11 (1306.65-1894.38) | 4.96 (4.19-6.04) | 4216.53 (3423.74-5071.26) | 4.84 (3.96-5.78) | -0.24 (-0.3 to -0.17) |
| Republic of the Union of Myanmar | 1611.87 (1017.18-2219.67) | 6.6 (4.28-8.94) | 2423.38 (1698.39-3393.54) | 4.77 (3.41-6.64) | -1.2 (-1.36 to -1.04) |
| Romania | 5951.51 (5276.2-6661.82) | 20.61 (18.25-23.08) | 10704.95 (9207.18-12356.94) | 33.31 (28.4-38.5) | 1.59 (1.41 to 1.77) |
| Russian Federation | 40289.33 (39137.04-41395.53) | 21.58 (20.96-22.17) | 36894.86 (32815.75-40324.65) | 15.52 (13.81-16.97) | -1.56 (-1.85 to -1.28) |
| Saint Kitts and Nevis | 2.46 (2.23-2.69) | 6.97 (6.32-7.69) | 7.03 (5.74-8.59) | 8.96 (7.37-10.81) | 1.29 (1.04 to 1.55) |
| Saint Lucia | 10.04 (9.38-10.79) | 11.5 (10.76-12.38) | 30.8 (24.96-37.47) | 12.47 (10.11-15.16) | 0.16 (0.03 to 0.29) |
| Saint Vincent and the Grenadines | 8.14 (7.4-8.96) | 11.46 (10.42-12.6) | 21.71 (19.07-25.19) | 14.71 (12.93-17.06) | 0.81 (0.64 to 0.98) |
| Slovak Republic | 1606.09 (1226.31-2108.16) | 27.5 (21.08-36.16) | 1828.66 (1329.32-2483.61) | 20.12 (14.48-27.45) | -1.01 (-1.07 to -0.95) |
| Socialist Republic of Viet Nam | 2830.21 (2202.33-3654.05) | 6.86 (5.37-8.84) | 12563.59 (9179.72-16868.92) | 11.58 (8.54-15.42) | 1.79 (1.75 to 1.83) |
| Solomon Islands | 4.39 (2.68-6.26) | 3.16 (2.04-4.46) | 10.96 (7.86-14.91) | 3 (2.2-4.02) | -0.19 (-0.29 to -0.1) |
| State of Eritrea | 112.76 (79.59-157.1) | 7.83 (5.68-10.67) | 221.2 (155.75-303.72) | 6.68 (4.83-8.96) | -0.78 (-0.87 to -0.69) |
| State of Israel | 586.47 (532.36-656.01) | 12.53 (11.35-14.03) | 1347.47 (1192.63-1520.47) | 11.63 (10.24-13.12) | -0.46 (-0.68 to -0.25) |
| State of Kuwait | 98 (86.7-109.31) | 14.09 (12.48-15.81) | 181.63 (148.44-224.32) | 5.77 (4.68-7.1) | -1.99 (-2.54 to -1.43) |
| State of Libya | 384.73 (283.05-520.61) | 19.46 (14.4-26.24) | 1470.32 (1065.91-2015.78) | 25.44 (18.51-34.34) | 1.23 (1.02 to 1.44) |
| State of Qatar | 20.52 (16.01-26.59) | 16.95 (13.03-21.75) | 203.62 (143.44-282.49) | 17.88 (12.44-24.76) | 0.38 (-0.11 to 0.87) |
| Sultanate of Oman | 36.69 (26.4-49.31) | 5.04 (3.66-6.72) | 112.54 (84.17-150.03) | 4.87 (3.72-6.41) | -0.04 (-0.21 to 0.13) |
| Swiss Confederation | 2042.85 (1848.75-2236.24) | 21.61 (19.53-23.73) | 2094.86 (1865.89-2341.55) | 12.8 (11.38-14.32) | -1.54 (-1.67 to -1.41) |
| Syrian Arab Republic | 459.25 (364.08-585.35) | 8.42 (6.7-10.79) | 1444.3 (1069.18-2031.18) | 10.22 (7.72-14.16) | 0.32 (0.18 to 0.47) |
| Taiwan (Province of China) | 1926.93 (1783.62-2081.28) | 11.26 (10.44-12.13) | 4490 (3970.54-4987.27) | 10.67 (9.39-11.87) | -0.41 (-0.65 to -0.18) |
| Togolese Republic | 54.8 (41.87-70.16) | 4.24 (3.27-5.39) | 235.21 (169.75-316.34) | 5.53 (4.03-7.38) | 1.07 (0.98 to 1.17) |
| Tokelau | 0.04 (0.03-0.05) | 2.77 (2.05-3.87) | 0.04 (0.03-0.05) | 2.63 (1.91-3.68) | -0.34 (-0.41 to -0.26) |
| Turkmenistan | 254.6 (236.73-272.83) | 12.16 (11.32-13.03) | 286.4 (224.39-367.54) | 6.32 (4.99-8.08) | -2.44 (-2.63 to -2.25) |
| Tuvalu | 0.19 (0.15-0.24) | 2.74 (2.15-3.43) | 0.31 (0.24-0.4) | 2.89 (2.26-3.7) | 0.12 (0.04 to 0.2) |
| Ukraine | 17629.63 (15847.77-19599.17) | 24.99 (22.36-27.98) | 11418.17 (7822.37-15763.67) | 15.78 (10.74-21.78) | -1.99 (-2.16 to -1.81) |
| Union of the Comoros | 16.1 (10.46-21.16) | 7.45 (5-9.73) | 31.5 (23.2-43.54) | 5.97 (4.43-8.14) | -1 (-1.17 to -0.83) |
| United Arab Emirates | 52.56 (33.94-73.91) | 10 (6.64-13.7) | 406.76 (295.4-531.04) | 8.4 (6.38-10.5) | 0.21 (-0.15 to 0.57) |
| United Kingdom of Great Britain and Northern Ireland | 16165.62 (15557.1-16854.4) | 19.18 (18.55-19.87) | 20621.03 (19644.28-21359.85) | 17.24 (16.53-17.84) | -0.35 (-0.45 to -0.25) |
| United Mexican States | 3715.21 (3608.1-3823.18) | 8.75 (8.49-9.01) | 5912.62 (5026.14-6852.47) | 4.6 (3.92-5.33) | -2.56 (-2.76 to -2.36) |
| United Republic of Tanzania | 921.99 (662.36-1276.27) | 7.84 (5.68-10.72) | 1633.06 (1143.69-2475.56) | 5.82 (4.16-8.67) | -1.23 (-1.31 to -1.14) |
| United States Virgin Islands | 8.78 (7.08-11.22) | 9.66 (7.8-12.23) | 11.83 (8.48-15.95) | 6.95 (5.01-9.23) | -1.07 (-1.29 to -0.85) |
| United States of America | 75975.55 (73396.1-78367.18) | 25.39 (24.6-26.13) | 107782.48 (102469.27-112333.18) | 18.94 (18.06-19.71) | -1.28 (-1.42 to -1.14) |

| **TableS2: Incidence cases and ASIR of larynx cancer in 1990 and 2021 and its trends** | | | | | |
| --- | --- | --- | --- | --- | --- |
| Location | Number 1990 | ASR 1990 | Number 2021 | ASR 2021 | EAPC_95%CI |
| American Samoa | 0.28 (0.22-0.36) | 1.32 (1.01-1.69) | 0.31 (0.25-0.4) | 0.68 (0.53-0.87) | -2.83 (-3.14 to -2.51) |
| Antigua and Barbuda | 1.13 (1.03-1.23) | 2.19 (1.99-2.39) | 2.4 (2.15-2.71) | 2.17 (1.94-2.44) | -0.02 (-0.33 to 0.29) |
| Arab Republic of Egypt | 354.64 (299.05-454.53) | 1.28 (1.09-1.64) | 1000.72 (765.72-1280.87) | 1.54 (1.21-1.95) | 0.86 (0.66 to 1.05) |
| Argentine Republic | 1451.49 (1328.81-1593.2) | 4.44 (4.07-4.86) | 1347.08 (1209.84-1486.86) | 2.44 (2.19-2.69) | -2.04 (-2.24 to -1.83) |
| Australia | 377.02 (340.47-420.72) | 1.96 (1.77-2.19) | 455.84 (403.91-509.76) | 1.08 (0.96-1.22) | -1.89 (-2.02 to -1.77) |
| Barbados | 5.01 (4.58-5.52) | 1.8 (1.66-1.97) | 10.83 (8.47-13.72) | 2.08 (1.63-2.65) | 0.45 (0.28 to 0.63) |
| Belize | 1.33 (1.23-1.43) | 1.44 (1.33-1.54) | 5.59 (4.83-6.55) | 1.82 (1.58-2.14) | 0.94 (0.43 to 1.46) |
| Bermuda | 2.11 (1.92-2.33) | 3.35 (3.05-3.7) | 3.82 (3.16-4.76) | 2.92 (2.4-3.64) | -0.11 (-0.3 to 0.09) |
| Bolivarian Republic of Venezuela | 292.46 (276.4-307.77) | 3.07 (2.89-3.24) | 754.09 (571.62-977.71) | 2.47 (1.88-3.2) | -1.25 (-1.45 to -1.06) |
| Bosnia and Herzegovina | 224.18 (190.01-262.35) | 4.95 (4.23-5.75) | 259.22 (185.28-334.29) | 4.27 (3.04-5.52) | -0.46 (-0.56 to -0.36) |
| Brunei Darussalam | 2.82 (2.22-3.53) | 2.92 (2.28-3.67) | 4.92 (3.8-6.38) | 1.43 (1.12-1.83) | -2.34 (-2.49 to -2.2) |
| Burkina Faso | 43.79 (32.35-56.05) | 0.99 (0.74-1.26) | 112.21 (78.26-151.74) | 1.21 (0.86-1.63) | 1.04 (0.88 to 1.2) |
| Canada | 1249.61 (1133.32-1363.26) | 3.92 (3.57-4.27) | 1304.1 (1160.71-1456.32) | 1.88 (1.67-2.1) | -2.24 (-2.41 to -2.07) |
| Central African Republic | 26.68 (15.78-36.84) | 2.18 (1.38-2.94) | 41.94 (24.45-61.69) | 1.72 (1.09-2.44) | -0.81 (-0.87 to -0.76) |
| Commonwealth of Dominica | 1.47 (1.23-1.75) | 2.48 (2.09-2.96) | 2.43 (1.84-3.17) | 2.8 (2.13-3.62) | 0.47 (0.41 to 0.54) |
| Commonwealth of the Bahamas | 4.99 (4.51-5.49) | 3.16 (2.86-3.47) | 13.38 (10.61-16.7) | 3.11 (2.48-3.86) | 0.22 (0.09 to 0.35) |
| Cook Islands | 0.08 (0.06-0.1) | 0.7 (0.55-0.85) | 0.15 (0.12-0.2) | 0.57 (0.44-0.78) | -0.8 (-1.2 to -0.39) |
| Czech Republic | 490.55 (425.13-564.21) | 3.68 (3.18-4.23) | 482.38 (384.94-598.34) | 2.49 (1.98-3.1) | -1.14 (-1.3 to -0.98) |
| Democratic People's Republic of Korea | 211.58 (149.09-284.62) | 1.23 (0.89-1.62) | 429.44 (301.28-573.35) | 1.26 (0.89-1.67) | 0.16 (0.1 to 0.23) |
| Democratic Republic of Sao Tome and Principe | 0.45 (0.35-0.57) | 0.69 (0.54-0.87) | 0.94 (0.75-1.21) | 0.88 (0.7-1.13) | 0.94 (0.85 to 1.03) |
| Democratic Republic of Timor-Leste | 3.06 (2.08-4.4) | 1.1 (0.77-1.54) | 9.11 (6.38-12.94) | 1.06 (0.75-1.51) | 0.07 (-0.17 to 0.31) |
| Democratic Republic of the Congo | 245.98 (171.25-333.16) | 1.55 (1.11-2.05) | 535.05 (372.86-719.94) | 1.38 (0.97-1.85) | -0.36 (-0.55 to -0.17) |
| Democratic Socialist Republic of Sri Lanka | 119.82 (96.49-150.85) | 1.11 (0.9-1.4) | 445.17 (258.59-675.37) | 1.6 (0.94-2.41) | 2.35 (1.9 to 2.81) |
| Dominican Republic | 64.77 (51.36-82.72) | 1.77 (1.41-2.23) | 188.04 (135.87-252.35) | 1.87 (1.35-2.51) | 0.47 (0.34 to 0.61) |
| Eastern Republic of Uruguay | 263.33 (242.52-285.88) | 6.86 (6.32-7.46) | 214.19 (192.77-238.63) | 4.2 (3.77-4.7) | -1.67 (-1.8 to -1.55) |
| Federal Democratic Republic of Ethiopia | 337.25 (186.86-485.24) | 1.59 (0.91-2.29) | 433.15 (307.37-588.05) | 0.97 (0.69-1.31) | -1.91 (-2.09 to -1.74) |
| Federal Democratic Republic of Nepal | 297.77 (202.38-420.22) | 3.06 (2.11-4.26) | 593.44 (431.9-824.17) | 2.49 (1.82-3.46) | -0.63 (-0.91 to -0.35) |
| Federal Republic of Germany | 3579.4 (3276.33-3924.2) | 3.06 (2.8-3.35) | 3866.98 (3454.11-4294.9) | 2.23 (1.99-2.48) | -1.29 (-1.44 to -1.14) |
| Federal Republic of Nigeria | 645.52 (449.68-877.13) | 1.41 (0.99-1.9) | 1096.79 (748.81-1508.87) | 1.18 (0.83-1.59) | -0.58 (-0.72 to -0.44) |
| Federal Republic of Somalia | 58.5 (37.31-85.51) | 2.19 (1.43-3.12) | 112.92 (69.46-163.92) | 1.65 (1.03-2.34) | -0.98 (-1.03 to -0.93) |
| Federated States of Micronesia | 0.42 (0.31-0.56) | 0.87 (0.65-1.16) | 0.57 (0.41-0.78) | 0.76 (0.56-1.05) | -0.53 (-0.59 to -0.48) |
| Federative Republic of Brazil | 3112.64 (2986.62-3236.96) | 3.33 (3.19-3.47) | 7744.66 (7237.06-8203.21) | 3.01 (2.81-3.19) | -0.34 (-0.43 to -0.25) |
| French Republic | 6514.63 (5910.08-7176.47) | 8.79 (7.98-9.66) | 5660.33 (4953.89-6435.13) | 4.82 (4.22-5.44) | -1.61 (-1.95 to -1.27) |
| Gabonese Republic | 12.28 (8.98-16.3) | 2.11 (1.56-2.76) | 20.17 (14.25-26.97) | 1.82 (1.32-2.44) | -0.53 (-0.58 to -0.48) |
| Georgia | 334.04 (307.31-362.25) | 5.12 (4.7-5.56) | 238.08 (205.56-273.07) | 4.09 (3.52-4.7) | -0.34 (-0.64 to -0.04) |
| Grand Duchy of Luxembourg | 27.91 (25.55-30.42) | 5.3 (4.84-5.77) | 29.27 (25.88-32.95) | 2.86 (2.53-3.23) | -1.88 (-2.02 to -1.73) |
| Greenland | 1.28 (1.02-1.56) | 3.33 (2.68-4.05) | 1.43 (1.12-1.85) | 1.87 (1.46-2.43) | -1.41 (-1.65 to -1.16) |
| Grenada | 1.19 (1.05-1.34) | 1.74 (1.55-1.97) | 1.76 (1.5-2.07) | 1.45 (1.24-1.69) | -0.21 (-0.75 to 0.33) |
| Guam | 0.58 (0.48-0.67) | 0.77 (0.64-0.89) | 0.8 (0.68-0.94) | 0.39 (0.33-0.45) | -2.24 (-2.63 to -1.85) |
| Hashemite Kingdom of Jordan | 26.83 (20.04-34.53) | 1.93 (1.45-2.48) | 98.98 (69.93-138.16) | 1.25 (0.88-1.74) | -1.86 (-2.13 to -1.6) |
| Hellenic Republic | 931.51 (864.59-992.48) | 6.12 (5.71-6.52) | 926.2 (840.54-1006.05) | 4.48 (4.05-4.87) | -0.97 (-1.07 to -0.87) |
| Hungary | 867.85 (777.65-966.9) | 6.13 (5.48-6.86) | 897 (741.72-1076.52) | 5.3 (4.36-6.44) | -0.95 (-1.16 to -0.74) |
| Independent State of Papua New Guinea | 9.69 (6.28-13.98) | 0.58 (0.38-0.83) | 24.8 (17.19-36.45) | 0.53 (0.37-0.77) | -0.32 (-0.38 to -0.26) |
| Independent State of Samoa | 0.29 (0.22-0.38) | 0.33 (0.26-0.43) | 0.48 (0.37-0.63) | 0.32 (0.25-0.42) | -0.26 (-0.35 to -0.18) |
| Ireland | 144.01 (130.74-160.1) | 3.65 (3.31-4.05) | 162.01 (140.71-185.99) | 2.14 (1.85-2.45) | -1.19 (-1.37 to -1.01) |
| Islamic Republic of Afghanistan | 235.86 (127.2-366.65) | 3.35 (1.84-5.1) | 307.44 (164.68-473.87) | 2.98 (1.69-4.34) | -0.48 (-0.57 to -0.4) |
| Islamic Republic of Iran | 880.96 (736.9-997.06) | 3.29 (2.75-3.75) | 2506.46 (2236.74-2843.25) | 3.18 (2.82-3.58) | -0.04 (-0.16 to 0.08) |
| Islamic Republic of Mauritania | 9.48 (7.36-12.1) | 0.95 (0.74-1.19) | 23.41 (16.28-33.62) | 1.09 (0.77-1.55) | 0.63 (0.4 to 0.87) |
| Islamic Republic of Pakistan | 3004.34 (2422.79-3707.65) | 5.25 (4.23-6.49) | 6462.21 (4735.92-8611.88) | 5.02 (3.67-6.65) | -0.45 (-0.66 to -0.24) |
| Jamaica | 25.35 (22.58-28.15) | 1.45 (1.29-1.62) | 52.36 (38.42-70.94) | 1.7 (1.25-2.31) | 0.59 (0.15 to 1.03) |
| Japan | 3233.03 (3074.79-3402.52) | 1.87 (1.77-1.96) | 3756.13 (3385.36-4017.34) | 1.07 (0.99-1.13) | -1.86 (-2.1 to -1.62) |
| Kingdom of Bahrain | 6.32 (5.11-8.1) | 3.8 (3.07-4.83) | 22.84 (16.38-32.98) | 2.53 (1.85-3.51) | -2.01 (-2.26 to -1.77) |
| Kingdom of Belgium | 875.34 (800.51-955.19) | 6.09 (5.56-6.63) | 609.44 (546.89-683) | 2.92 (2.62-3.26) | -2.46 (-2.59 to -2.33) |
| Kingdom of Bhutan | 6.66 (4.31-9.67) | 2.59 (1.69-3.74) | 13.47 (9.24-18.68) | 2.21 (1.52-3.05) | -0.53 (-0.67 to -0.4) |
| Kingdom of Cambodia | 86.1 (64.26-115.72) | 1.89 (1.43-2.51) | 225.73 (156.6-340.64) | 1.78 (1.24-2.66) | -0.26 (-0.43 to -0.09) |
| Kingdom of Denmark | 306.76 (280.1-334.61) | 4.16 (3.79-4.55) | 278.92 (251.59-307.65) | 2.54 (2.29-2.8) | -1.61 (-1.73 to -1.49) |
| Kingdom of Eswatini | 9.32 (6.05-12.82) | 3.04 (1.97-4.12) | 17.9 (10.65-26.04) | 2.88 (1.74-4.15) | 0.06 (-0.33 to 0.45) |
| Kingdom of Lesotho | 18.74 (14.25-24.33) | 2.15 (1.65-2.8) | 39.12 (26.52-53.36) | 3.35 (2.29-4.55) | 1.88 (1.57 to 2.18) |
| Kingdom of Morocco | 321.23 (243.63-400.88) | 2.24 (1.72-2.78) | 869.65 (602.23-1160.02) | 2.45 (1.71-3.24) | 0.39 (0.18 to 0.6) |
| Kingdom of Norway | 114.38 (105.63-124.05) | 1.82 (1.68-1.97) | 130.12 (116.57-144.68) | 1.33 (1.19-1.48) | -1.17 (-1.46 to -0.87) |
| Kingdom of Saudi Arabia | 63.81 (46.76-83.75) | 1.07 (0.79-1.4) | 253.48 (192.02-339.12) | 1.12 (0.87-1.45) | -0.04 (-0.11 to 0.04) |
| Kingdom of Spain | 4117.87 (3607.1-4598.87) | 7.95 (6.97-8.91) | 3628.09 (3122.9-4248.79) | 4.2 (3.61-4.97) | -2.27 (-2.42 to -2.12) |
| Kingdom of Sweden | 189.19 (170.22-209.26) | 1.33 (1.2-1.48) | 201.41 (173.1-231.65) | 0.98 (0.84-1.14) | -0.63 (-0.87 to -0.39) |
| Kingdom of Thailand | 933.67 (760.72-1121.45) | 2.58 (2.09-3.07) | 2509.3 (1843.16-3286.65) | 2.29 (1.69-3) | -0.7 (-0.83 to -0.58) |
| Kingdom of Tonga | 0.35 (0.25-0.48) | 0.65 (0.48-0.91) | 0.5 (0.35-0.69) | 0.63 (0.44-0.87) | -0.22 (-0.42 to -0.01) |
| Kingdom of the Netherlands | 705.34 (648.93-770.32) | 3.69 (3.39-4.04) | 723.16 (646.95-800.13) | 2.14 (1.91-2.37) | -1.81 (-1.9 to -1.71) |
| Kyrgyz Republic | 72.55 (60.58-85.94) | 2.3 (1.93-2.72) | 51.03 (38.32-65.32) | 0.95 (0.72-1.22) | -2.83 (-3.01 to -2.66) |
| Lao People's Democratic Republic | 38.68 (26.84-53.86) | 1.81 (1.28-2.49) | 60.18 (41.41-88.91) | 1.3 (0.91-1.9) | -1.12 (-1.25 to -0.99) |
| Lebanese Republic | 87.6 (59.33-119.82) | 3.91 (2.72-5.29) | 218.71 (174.72-274.19) | 3.7 (2.95-4.65) | 0.26 (0.05 to 0.46) |
| Malaysia | 174.25 (132.92-214.09) | 1.9 (1.43-2.33) | 546.9 (427.36-655.38) | 1.89 (1.48-2.27) | -0.17 (-0.38 to 0.05) |
| Mongolia | 16.76 (12.15-21.84) | 1.53 (1.11-1.98) | 24.7 (17.77-34.41) | 0.94 (0.7-1.27) | -1.86 (-2.08 to -1.64) |
| Montenegro | 55.37 (43.75-69.76) | 8.41 (6.66-10.63) | 84.97 (65.25-112.88) | 8.71 (6.69-11.62) | 0.23 (0.06 to 0.39) |
| New Zealand | 104.39 (93.04-116.31) | 2.67 (2.38-2.97) | 125.46 (107.88-143.79) | 1.49 (1.28-1.71) | -1.93 (-2.19 to -1.67) |
| North Macedonia | 100.21 (81.8-124.96) | 5.07 (4.14-6.32) | 171.79 (128.2-225.09) | 5 (3.74-6.51) | -0.03 (-0.33 to 0.27) |
| Northern Mariana Islands | 0.25 (0.18-0.35) | 1.52 (1.13-1.95) | 0.48 (0.39-0.58) | 0.96 (0.78-1.14) | -2.24 (-2.64 to -1.83) |
| Palestine | 14.39 (10.67-18.9) | 1.7 (1.26-2.23) | 36.52 (29.18-45.61) | 1.42 (1.16-1.76) | -0.87 (-1.08 to -0.65) |
| People's Democratic Republic of Algeria | 259.45 (198.6-343.96) | 2.23 (1.71-2.91) | 666.15 (488.8-891.03) | 1.88 (1.4-2.53) | -0.51 (-0.69 to -0.33) |
| People's Republic of Bangladesh | 1932.42 (1512.02-2426.46) | 4.03 (3.14-5.08) | 3698.02 (2628.53-4996.26) | 2.63 (1.88-3.56) | -1.45 (-1.57 to -1.34) |
| People's Republic of China | 15434.15 (12624.19-18174.01) | 1.82 (1.5-2.13) | 38904.86 (30369.67-49486.18) | 1.79 (1.4-2.26) | 0.04 (-0.1 to 0.19) |
| Plurinational State of Bolivia | 51.28 (37.02-67.5) | 1.62 (1.17-2.12) | 105.54 (72.89-150.09) | 1.17 (0.82-1.65) | -1.04 (-1.15 to -0.92) |
| Portuguese Republic | 558.95 (508.44-616.7) | 4.11 (3.74-4.53) | 513.98 (457.42-583.32) | 2.44 (2.18-2.76) | -1.68 (-1.88 to -1.48) |
| Principality of Andorra | 1.98 (1.33-3.1) | 3.37 (2.26-5.24) | 3.31 (1.98-4.91) | 2.16 (1.29-3.19) | -1.29 (-1.54 to -1.04) |
| Principality of Monaco | 8.39 (6.05-11.31) | 14.17 (10.15-19.05) | 8.56 (6.49-11.41) | 10.44 (7.8-14.22) | -1.03 (-1.13 to -0.93) |
| Puerto Rico | 114.01 (101.6-126.1) | 3.15 (2.81-3.49) | 102.97 (81.86-125.59) | 1.62 (1.28-2) | -1.88 (-2.2 to -1.56) |
| Republic of Albania | 78.94 (63.2-96.52) | 3.69 (2.96-4.53) | 138.89 (96.78-195.88) | 3.24 (2.29-4.54) | 0.01 (-0.18 to 0.19) |
| Republic of Angola | 79.75 (53.76-111.25) | 1.96 (1.36-2.7) | 200.04 (150.82-259.76) | 1.62 (1.24-2.09) | -0.59 (-0.74 to -0.44) |
| Republic of Armenia | 148.18 (140.58-155.02) | 4.98 (4.74-5.2) | 107.42 (94.89-120.66) | 2.44 (2.16-2.74) | -2.3 (-2.56 to -2.04) |
| Republic of Austria | 360.89 (328.24-397.2) | 3.35 (3.03-3.7) | 320.08 (282.06-357.03) | 1.93 (1.71-2.15) | -1.73 (-1.88 to -1.58) |
| Republic of Azerbaijan | 184.98 (161.61-214.54) | 3.4 (2.94-3.96) | 258.48 (188.67-366.75) | 2.25 (1.66-3.16) | -1.37 (-1.5 to -1.23) |
| Republic of Belarus | 630.44 (551.12-722.06) | 4.77 (4.18-5.45) | 650.16 (491.82-840.85) | 4.11 (3.1-5.3) | -1.63 (-2.01 to -1.25) |
| Republic of Benin | 19.4 (15.34-23.95) | 0.98 (0.78-1.21) | 55.2 (39.94-72.55) | 1.07 (0.79-1.38) | 0.53 (0.41 to 0.65) |
| Republic of Botswana | 16.15 (11.72-20.59) | 2.71 (1.98-3.41) | 30.58 (21.5-47.44) | 1.94 (1.38-2.93) | -1.46 (-1.75 to -1.17) |
| Republic of Bulgaria | 544.64 (466.66-636.26) | 4.41 (3.77-5.16) | 716.41 (571.08-889.87) | 5.63 (4.47-7.02) | 1.01 (0.79 to 1.24) |
| Republic of Burundi | 55.87 (35.38-77.5) | 2.31 (1.48-3.19) | 76.67 (53.29-110.04) | 1.47 (1.03-2.06) | -1.84 (-2.08 to -1.6) |
| Republic of Cabo Verde | 3.82 (2.95-4.79) | 1.66 (1.29-2.08) | 8.15 (5.52-10.63) | 1.79 (1.23-2.3) | 0.02 (-0.49 to 0.53) |
| Republic of Cameroon | 48.79 (37.94-63.89) | 1.07 (0.84-1.38) | 172.7 (113.9-246.99) | 1.34 (0.89-1.89) | 0.97 (0.83 to 1.11) |
| Republic of Chad | 22.82 (17.43-29.34) | 0.81 (0.62-1.05) | 78.81 (54.61-105.88) | 1.36 (0.95-1.81) | 1.98 (1.81 to 2.15) |
| Republic of Chile | 199.36 (179.95-220.71) | 1.95 (1.76-2.16) | 292.71 (260.87-326.31) | 1.14 (1.02-1.27) | -1.58 (-1.7 to -1.45) |
| Republic of Colombia | 485.97 (445.97-523.67) | 2.79 (2.57-3.01) | 677.23 (546.77-825.52) | 1.23 (0.99-1.5) | -3.31 (-3.51 to -3.1) |
| Republic of Costa Rica | 39.24 (35.61-43.21) | 2.27 (2.05-2.5) | 60.45 (51.78-70.81) | 1.09 (0.94-1.28) | -2.45 (-2.65 to -2.25) |
| Republic of Croatia | 426.45 (385.18-472.8) | 6.61 (5.98-7.32) | 328.49 (273.49-383.63) | 4.09 (3.38-4.82) | -1.55 (-1.71 to -1.38) |
| Republic of Cuba | 588.17 (530.1-654.62) | 5.77 (5.2-6.42) | 1521.6 (1259.15-1842.31) | 7.8 (6.47-9.43) | 1.1 (0.96 to 1.24) |
| Republic of Cyprus | 18.97 (14.9-23.49) | 2.47 (1.94-3.05) | 43.59 (33.86-55.9) | 2.14 (1.67-2.74) | 0.02 (-0.17 to 0.21) |
| Republic of C么te d'Ivoire | 57.12 (44.65-73.31) | 1.34 (1.07-1.69) | 148.1 (101.71-224.13) | 1.23 (0.86-1.82) | -0.53 (-0.69 to -0.38) |
| Republic of Djibouti | 3.19 (2.16-4.81) | 2.1 (1.45-3.09) | 13.31 (8.86-19.19) | 1.88 (1.28-2.63) | -0.5 (-0.58 to -0.42) |
| Republic of Ecuador | 58.01 (53.71-62.96) | 1.11 (1.03-1.21) | 110.55 (84.06-141.67) | 0.68 (0.52-0.87) | -1.62 (-1.89 to -1.36) |
| Republic of El Salvador | 30.26 (26.03-35.22) | 1.03 (0.88-1.2) | 55.21 (42.64-70.68) | 0.9 (0.7-1.16) | -0.78 (-0.99 to -0.58) |
| Republic of Equatorial Guinea | 3.96 (2.39-5.76) | 1.95 (1.22-2.77) | 7.49 (4.47-11.19) | 1.43 (0.86-2.1) | -1.1 (-1.46 to -0.73) |
| Republic of Estonia | 89.31 (78.29-101.9) | 4.36 (3.84-4.95) | 77.31 (62.78-96.02) | 3.32 (2.69-4.15) | -1.3 (-1.59 to -1.01) |
| Republic of Fiji | 2.83 (2.22-3.64) | 0.81 (0.64-1.03) | 5.97 (4.32-8.03) | 0.79 (0.57-1.04) | -0.01 (-0.14 to 0.11) |
| Republic of Finland | 142.42 (130.24-156.42) | 2.09 (1.91-2.31) | 172.44 (152.2-193.24) | 1.59 (1.41-1.79) | -0.88 (-1.07 to -0.69) |
| Republic of Ghana | 53.39 (40.91-70.42) | 0.84 (0.66-1.1) | 205.07 (144.82-266.42) | 1.2 (0.85-1.55) | 1.77 (1.53 to 2) |
| Republic of Guatemala | 53.7 (51.16-56.48) | 1.65 (1.57-1.74) | 60.77 (51.91-71.43) | 0.56 (0.48-0.65) | -3.7 (-3.91 to -3.5) |
| Republic of Guinea | 27.97 (21.04-36.1) | 0.84 (0.64-1.09) | 71.81 (52.53-92.81) | 1.28 (0.94-1.65) | 1.87 (1.68 to 2.05) |
| Republic of Guinea-Bissau | 5.75 (3.61-8.09) | 1.42 (0.92-1.97) | 11.88 (8.04-16.47) | 1.58 (1.08-2.13) | 0.79 (0.64 to 0.95) |
| Republic of Guyana | 4.87 (4.23-5.69) | 1.27 (1.1-1.48) | 8.36 (6.18-11.11) | 1.22 (0.91-1.61) | 0.41 (0.19 to 0.63) |
| Republic of Haiti | 93.27 (62.63-124.04) | 2.83 (1.95-3.72) | 169.37 (109.09-238.25) | 2.34 (1.5-3.28) | -0.44 (-0.53 to -0.36) |
| Republic of Honduras | 24.52 (19.9-30.77) | 1.21 (0.98-1.51) | 98.1 (73.46-127.4) | 1.58 (1.18-2.04) | 1.09 (0.97 to 1.22) |
| Republic of Iceland | 5.56 (4.95-6.23) | 2.04 (1.82-2.29) | 6.91 (5.99-7.9) | 1.27 (1.11-1.46) | -1.73 (-1.88 to -1.58) |
| Republic of India | 15644.05 (12795.05-18673.63) | 3.15 (2.56-3.77) | 35121.49 (30597.62-40702.52) | 2.84 (2.48-3.29) | -0.47 (-0.65 to -0.29) |
| Republic of Indonesia | 1189.87 (894.3-1441.75) | 1.19 (0.88-1.44) | 3098.18 (2201.42-4110.87) | 1.26 (0.89-1.66) | 0.18 (0.15 to 0.22) |
| Republic of Iraq | 242.66 (188.01-308.88) | 3.02 (2.32-3.84) | 794.8 (553.5-1050.3) | 3.23 (2.28-4.28) | -0.06 (-0.18 to 0.07) |
| Republic of Italy | 5157.81 (4837.21-5486.21) | 5.97 (5.59-6.34) | 3900.73 (3585.87-4256.04) | 3.02 (2.77-3.29) | -2.21 (-2.31 to -2.11) |
| Republic of Kazakhstan | 525.17 (481.02-566.6) | 3.86 (3.53-4.17) | 323.23 (279.18-370.82) | 1.63 (1.41-1.88) | -3.32 (-3.53 to -3.11) |
| Republic of Kenya | 106.13 (77.3-144.52) | 1.24 (0.9-1.69) | 359.11 (267.09-467.35) | 1.45 (1.07-1.86) | 0.54 (0.36 to 0.72) |
| Republic of Kiribati | 0.08 (0.06-0.1) | 0.21 (0.16-0.27) | 0.17 (0.12-0.24) | 0.23 (0.16-0.32) | 0.17 (0.07 to 0.27) |
| Republic of Korea | 1242.36 (862.79-1590.51) | 4.04 (2.84-5.14) | 2058.2 (1517.22-2688.66) | 2.15 (1.59-2.8) | -2.74 (-3.02 to -2.46) |
| Republic of Latvia | 149.19 (133.51-167.2) | 4.18 (3.74-4.7) | 98.48 (82.08-116.98) | 2.89 (2.37-3.43) | -1.51 (-1.73 to -1.29) |
| Republic of Liberia | 10.58 (7.54-15.4) | 0.93 (0.67-1.36) | 23.69 (15.95-34.04) | 1.12 (0.77-1.57) | 0.8 (0.64 to 0.97) |
| Republic of Lithuania | 208.13 (181.26-240.15) | 4.61 (4.02-5.31) | 174.38 (138.3-215.07) | 3.44 (2.73-4.25) | -1.21 (-1.44 to -0.97) |
| Republic of Madagascar | 88.33 (64.62-110.9) | 1.66 (1.23-2.09) | 140.91 (99.05-191.67) | 1.13 (0.79-1.52) | -1.31 (-1.45 to -1.17) |
| Republic of Malawi | 24.22 (18.55-32.43) | 0.6 (0.46-0.79) | 50.1 (37.58-70.74) | 0.62 (0.47-0.86) | -0.11 (-0.29 to 0.07) |
| Republic of Maldives | 1.16 (0.82-1.5) | 1.37 (0.99-1.72) | 2.57 (1.88-3.37) | 0.77 (0.57-0.99) | -2.12 (-2.25 to -1.99) |
| Republic of Mali | 45.17 (37.43-54.92) | 1.09 (0.92-1.31) | 94.01 (69.56-128.41) | 1.03 (0.77-1.39) | 0.08 (-0.08 to 0.23) |
| Republic of Malta | 16.57 (14.38-18.87) | 3.86 (3.35-4.4) | 19.29 (16.25-22.99) | 2.31 (1.93-2.76) | -1.71 (-1.8 to -1.63) |
| Republic of Mauritius | 20.53 (19.18-22.31) | 2.73 (2.55-2.96) | 43.95 (39.97-47.54) | 2.31 (2.11-2.49) | -0.34 (-1.07 to 0.39) |
| Republic of Moldova | 200.8 (188.39-213.89) | 4.33 (4.07-4.6) | 228.26 (200.54-260.14) | 3.82 (3.35-4.35) | -0.47 (-0.81 to -0.14) |
| Republic of Mozambique | 119.89 (85.98-160.36) | 1.9 (1.39-2.52) | 234.28 (170.18-307.15) | 1.92 (1.4-2.48) | 0.39 (0.26 to 0.52) |
| Republic of Namibia | 18.45 (14.91-22.69) | 2.68 (2.18-3.29) | 42.97 (30.71-58.64) | 2.87 (2.09-3.83) | 0.05 (-0.2 to 0.31) |
| Republic of Nauru | 0.05 (0.03-0.07) | 1.15 (0.77-1.54) | 0.05 (0.03-0.07) | 0.87 (0.59-1.2) | -1.06 (-1.24 to -0.88) |
| Republic of Nicaragua | 19.83 (15.99-24.04) | 1.33 (1.07-1.61) | 46.67 (35.92-60.92) | 0.96 (0.74-1.25) | -1.09 (-1.24 to -0.94) |
| Republic of Niue | 0.01 (0.01-0.02) | 0.63 (0.46-0.83) | 0.01 (0.01-0.02) | 0.65 (0.49-0.86) | -0.16 (-0.31 to -0.01) |
| Republic of Palau | 0.07 (0.05-0.09) | 0.69 (0.51-0.91) | 0.13 (0.1-0.18) | 0.6 (0.43-0.78) | -0.62 (-0.74 to -0.5) |
| Republic of Panama | 27.28 (25.29-29.08) | 1.88 (1.74-2) | 44.14 (34.37-53.79) | 1 (0.78-1.22) | -2.36 (-2.55 to -2.16) |
| Republic of Paraguay | 34.46 (26.64-44.02) | 1.54 (1.19-1.96) | 130.22 (90.11-179.23) | 2.18 (1.52-3) | 1.27 (1.19 to 1.35) |
| Republic of Peru | 144.77 (116.3-175.65) | 1.23 (0.99-1.5) | 259.21 (184.21-362.71) | 0.78 (0.55-1.09) | -1.91 (-2.32 to -1.5) |
| Republic of Poland | 2263.35 (2170.05-2361.1) | 5.18 (4.98-5.41) | 2679.84 (2398.02-2952.34) | 3.92 (3.51-4.32) | -1.13 (-1.34 to -0.93) |
| Republic of Rwanda | 80.85 (57.15-104.82) | 2.69 (1.92-3.49) | 106.47 (72.61-155.69) | 1.58 (1.09-2.29) | -2.61 (-2.95 to -2.26) |
| Republic of San Marino | 1.95 (1.48-2.45) | 5.86 (4.47-7.39) | 1.72 (1.03-2.59) | 2.64 (1.54-3.99) | -1.85 (-2.18 to -1.52) |
| Republic of Senegal | 34.2 (27.11-41.87) | 1.05 (0.83-1.28) | 96.27 (70.51-125.97) | 1.23 (0.9-1.6) | 0.85 (0.66 to 1.04) |
| Republic of Serbia | 673.15 (480.93-953.62) | 5.59 (3.98-7.85) | 846.76 (589.89-1158.95) | 5.55 (3.82-7.62) | -0.1 (-0.32 to 0.12) |
| Republic of Seychelles | 3.65 (3.09-4.31) | 6.49 (5.5-7.68) | 7.14 (5.8-8.68) | 5.87 (4.78-7.08) | 0.34 (0.06 to 0.62) |
| Republic of Sierra Leone | 20.46 (15.36-26.57) | 1.01 (0.76-1.3) | 43.62 (30.89-61) | 1.15 (0.83-1.58) | 0.88 (0.69 to 1.07) |
| Republic of Singapore | 76.75 (68.48-85.38) | 3.42 (3.05-3.78) | 141.28 (121.82-163.22) | 1.62 (1.4-1.86) | -2.86 (-3.21 to -2.51) |
| Republic of Slovenia | 107.15 (96.39-120.34) | 4.32 (3.89-4.85) | 125.04 (103.76-149.55) | 3.14 (2.6-3.77) | -1.03 (-1.21 to -0.84) |
| Republic of South Africa | 476.49 (386.22-647.06) | 2.19 (1.77-3) | 961.27 (842.36-1083.06) | 1.96 (1.72-2.19) | -0.66 (-0.92 to -0.4) |
| Republic of South Sudan | 54.61 (37.66-73.73) | 2.07 (1.44-2.78) | 68.82 (44.45-101.28) | 1.64 (1.07-2.37) | -0.99 (-1.18 to -0.8) |
| Republic of Sudan | 241.98 (148.1-379.85) | 2.58 (1.57-4.12) | 462.58 (281.13-698.27) | 2.31 (1.45-3.44) | -0.48 (-0.55 to -0.41) |
| Republic of Suriname | 2.53 (2.09-3) | 0.98 (0.8-1.15) | 6.1 (4.33-8.46) | 0.93 (0.66-1.3) | 0.05 (-0.16 to 0.25) |
| Republic of Tajikistan | 50.72 (37.56-64.45) | 1.75 (1.31-2.22) | 63.9 (43.55-88.71) | 0.98 (0.68-1.37) | -2.01 (-2.22 to -1.8) |
| Republic of Trinidad and Tobago | 13.78 (12.85-14.82) | 1.65 (1.54-1.77) | 29.99 (22.62-38.74) | 1.52 (1.15-1.96) | -0.31 (-0.45 to -0.18) |
| Republic of Tunisia | 177.66 (135.62-228.49) | 3.49 (2.68-4.46) | 515.23 (358.05-733.81) | 3.76 (2.63-5.33) | 0.04 (-0.02 to 0.11) |
| Republic of Turkey | 1537.06 (1109.36-2072.44) | 4.34 (3.15-5.88) | 3166.48 (2435.04-4114.65) | 3.31 (2.54-4.27) | -1.05 (-1.24 to -0.86) |
| Republic of Uganda | 155.48 (116.06-199.39) | 2.33 (1.76-2.96) | 302.96 (207.97-426.38) | 1.91 (1.35-2.65) | -1.33 (-1.57 to -1.08) |
| Republic of Uzbekistan | 297.2 (258.85-340.47) | 2.46 (2.15-2.8) | 223.29 (174.9-285.26) | 0.78 (0.62-0.98) | -3.39 (-4.02 to -2.75) |
| Republic of Vanuatu | 0.37 (0.24-0.53) | 0.63 (0.43-0.86) | 0.87 (0.6-1.2) | 0.52 (0.37-0.71) | -0.79 (-0.88 to -0.69) |
| Republic of Yemen | 149.77 (93.38-216.07) | 2.92 (1.83-4.13) | 407 (248.3-590.02) | 2.83 (1.75-4.1) | -0.14 (-0.19 to -0.09) |
| Republic of Zambia | 64.84 (48.44-82.08) | 2.17 (1.61-2.74) | 176.18 (85.43-408.47) | 2.26 (1.17-5.01) | -0.09 (-0.23 to 0.05) |
| Republic of Zimbabwe | 96.12 (73.62-121.8) | 2.26 (1.75-2.84) | 187.28 (139.03-252.71) | 2.43 (1.85-3.21) | 0.25 (-0.02 to 0.51) |
| Republic of the Congo | 23.81 (14.98-31.68) | 2.15 (1.38-2.81) | 48.44 (35.35-64.67) | 1.71 (1.29-2.26) | -0.94 (-1.12 to -0.76) |
| Republic of the Gambia | 1.93 (1.47-2.5) | 0.53 (0.4-0.68) | 5.15 (3.84-6.67) | 0.51 (0.38-0.66) | -0.24 (-0.4 to -0.08) |
| Republic of the Marshall Islands | 0.13 (0.09-0.19) | 0.83 (0.59-1.15) | 0.27 (0.18-0.39) | 0.83 (0.55-1.19) | 0.13 (0.01 to 0.24) |
| Republic of the Niger | 24.88 (17.94-32.87) | 0.88 (0.64-1.16) | 78.4 (52.35-112.1) | 0.97 (0.66-1.36) | 0.74 (0.58 to 0.9) |
| Republic of the Philippines | 330.36 (275.96-404.03) | 1.1 (0.92-1.35) | 883.98 (709.81-1067.63) | 1.04 (0.84-1.25) | -0.25 (-0.32 to -0.18) |
| Republic of the Union of Myanmar | 406.2 (256.51-561.84) | 1.72 (1.12-2.35) | 526.44 (367.75-752.73) | 1.07 (0.76-1.51) | -1.74 (-1.88 to -1.6) |
| Romania | 1198.27 (1049.58-1347.27) | 4.16 (3.63-4.68) | 1895.44 (1610.69-2201.24) | 5.82 (4.92-6.78) | 1.02 (0.83 to 1.21) |
| Russian Federation | 8438.39 (8214.72-8644.13) | 4.52 (4.4-4.63) | 6866.92 (6067.49-7555.17) | 2.88 (2.54-3.17) | -2.06 (-2.33 to -1.79) |
| Saint Kitts and Nevis | 0.64 (0.58-0.7) | 1.75 (1.58-1.92) | 1.51 (1.23-1.84) | 2.01 (1.64-2.41) | 0.87 (0.62 to 1.12) |
| Saint Lucia | 2.37 (2.21-2.54) | 2.74 (2.57-2.95) | 6.53 (5.26-7.97) | 2.67 (2.15-3.25) | -0.22 (-0.4 to -0.05) |
| Saint Vincent and the Grenadines | 1.92 (1.75-2.12) | 2.7 (2.46-2.97) | 4.79 (4.18-5.58) | 3.28 (2.87-3.81) | 0.62 (0.44 to 0.81) |
| Slovak Republic | 326.47 (245.56-432.9) | 5.57 (4.22-7.35) | 325.46 (231.18-447.67) | 3.56 (2.52-4.93) | -1.44 (-1.51 to -1.37) |
| Socialist Republic of Viet Nam | 630.13 (488.17-828.07) | 1.55 (1.21-2.02) | 2374.05 (1725.81-3210.29) | 2.24 (1.65-2.98) | 1.25 (1.2 to 1.29) |
| Solomon Islands | 1.1 (0.66-1.6) | 0.86 (0.54-1.22) | 2.58 (1.82-3.55) | 0.75 (0.53-1.04) | -0.44 (-0.52 to -0.36) |
| State of Eritrea | 29.17 (20.27-40.61) | 2.15 (1.52-2.94) | 51.61 (35.52-71.09) | 1.63 (1.15-2.23) | -1.12 (-1.21 to -1.03) |
| State of Israel | 101.12 (91.15-113.94) | 2.16 (1.95-2.43) | 206.99 (180.31-236.53) | 1.77 (1.55-2.02) | -0.96 (-1.19 to -0.73) |
| State of Kuwait | 17.43 (15.32-19.51) | 2.61 (2.3-2.93) | 27.93 (22.49-34.68) | 0.92 (0.73-1.14) | -2.42 (-3.02 to -1.81) |
| State of Libya | 79.32 (56.98-107.97) | 4.11 (2.98-5.61) | 269.42 (192.23-371.96) | 4.8 (3.43-6.51) | 0.85 (0.66 to 1.04) |
| State of Qatar | 3.89 (2.91-5.13) | 3.71 (2.77-4.89) | 30.92 (20.97-43.1) | 2.99 (2.07-4.2) | -0.65 (-1.21 to -0.08) |
| Sultanate of Oman | 7.5 (5.21-10.34) | 1.06 (0.75-1.45) | 18.84 (13.8-25.42) | 0.86 (0.65-1.16) | -0.52 (-0.68 to -0.36) |
| Swiss Confederation | 336.24 (301.75-368.41) | 3.52 (3.16-3.87) | 301.01 (262.82-340.37) | 1.83 (1.6-2.07) | -1.9 (-2.02 to -1.78) |
| Syrian Arab Republic | 93.8 (71.15-122.66) | 1.77 (1.35-2.34) | 254.05 (184.1-360.36) | 1.88 (1.39-2.62) | -0.08 (-0.24 to 0.08) |
| Taiwan (Province of China) | 351.8 (327.05-379.47) | 2.1 (1.95-2.26) | 715.86 (626.56-799.92) | 1.7 (1.48-1.89) | -0.99 (-1.22 to -0.76) |
| Togolese Republic | 13.15 (9.83-16.97) | 1.06 (0.8-1.37) | 54.59 (38.87-73.8) | 1.35 (0.97-1.83) | 1.03 (0.93 to 1.14) |
| Tokelau | 0.01 (0.01-0.01) | 0.69 (0.5-0.98) | 0.01 (0.01-0.01) | 0.58 (0.41-0.82) | -0.74 (-0.83 to -0.64) |
| Turkmenistan | 57.08 (52.93-61.4) | 2.77 (2.57-2.99) | 56.82 (42.82-74.95) | 1.27 (0.97-1.68) | -2.9 (-3.13 to -2.67) |
| Tuvalu | 0.05 (0.04-0.06) | 0.71 (0.55-0.91) | 0.07 (0.05-0.09) | 0.67 (0.51-0.86) | -0.23 (-0.33 to -0.14) |
| Ukraine | 3527.49 (3149.97-3929.21) | 4.97 (4.42-5.58) | 2098.77 (1366.65-2981.57) | 2.88 (1.87-4.1) | -2.34 (-2.53 to -2.14) |
| Union of the Comoros | 3.95 (2.57-5.23) | 1.89 (1.27-2.49) | 7.23 (5.26-10.04) | 1.41 (1.04-1.95) | -1.25 (-1.44 to -1.06) |
| United Arab Emirates | 10.27 (6.25-14.87) | 2.09 (1.29-2.95) | 65.7 (45.39-88.46) | 1.57 (1.15-2.01) | 0.1 (-0.33 to 0.53) |
| United Kingdom of Great Britain and Northern Ireland | 2634.62 (2561.77-2697.44) | 3.09 (3.01-3.16) | 3080.62 (2918.56-3191.9) | 2.55 (2.43-2.63) | -0.66 (-0.76 to -0.55) |
| United Mexican States | 894.93 (869.37-918.42) | 2.21 (2.14-2.27) | 1262.16 (1071.41-1464.7) | 1 (0.85-1.16) | -2.97 (-3.14 to -2.79) |
| United Republic of Tanzania | 226.12 (161.44-310.84) | 1.98 (1.42-2.7) | 369.04 (255.75-564.77) | 1.36 (0.96-2.02) | -1.55 (-1.66 to -1.44) |
| United States Virgin Islands | 1.87 (1.49-2.43) | 2.12 (1.69-2.74) | 2.52 (1.77-3.44) | 1.44 (1.02-1.94) | -1.2 (-1.41 to -0.99) |
| United States of America | 12275.38 (11852.1-12580.97) | 4.07 (3.94-4.17) | 16371.45 (15509.4-17060.58) | 2.87 (2.72-2.98) | -1.48 (-1.61 to -1.36) |

| **TableS3: Deaths cases and ASDR of larynx cancer in 1990 and 2021 and its trends** | | | | | |
| --- | --- | --- | --- | --- | --- |
| Location | Number 1990 | ASR 1990 | Number 2021 | ASR 2021 | EAPC_95%CI |
| American Samoa | 0.22 (0.17-0.28) | 1.14 (0.87-1.45) | 0.24 (0.19-0.31) | 0.55 (0.43-0.7) | -2.93 (-3.23 to -2.63) |
| Antigua and Barbuda | 0.93 (0.84-1.01) | 1.76 (1.6-1.9) | 1.67 (1.49-1.88) | 1.56 (1.39-1.74) | -0.36 (-0.68 to -0.04) |
| Arab Republic of Egypt | 299.41 (252.6-380.2) | 1.16 (0.98-1.44) | 647.42 (498.75-835.62) | 1.09 (0.86-1.37) | 0.19 (-0.03 to 0.41) |
| Argentine Republic | 1094.74 (1009.37-1198.14) | 3.37 (3.11-3.68) | 891.52 (805.87-977.75) | 1.59 (1.44-1.74) | -2.41 (-2.58 to -2.23) |
| Australia | 242.08 (220.19-268.27) | 1.24 (1.13-1.37) | 234.11 (204.76-262.32) | 0.51 (0.45-0.57) | -2.97 (-3.07 to -2.87) |
| Barbados | 4.13 (3.77-4.55) | 1.43 (1.31-1.56) | 7.79 (6.17-9.74) | 1.48 (1.17-1.86) | 0.1 (-0.08 to 0.28) |
| Belize | 1.14 (1.06-1.22) | 1.24 (1.15-1.33) | 4.33 (3.71-5.02) | 1.47 (1.27-1.7) | 0.66 (0.1 to 1.22) |
| Bermuda | 1.47 (1.34-1.61) | 2.36 (2.16-2.58) | 1.87 (1.55-2.32) | 1.36 (1.12-1.69) | -1.44 (-1.64 to -1.24) |
| Bolivarian Republic of Venezuela | 251.75 (237.94-264.36) | 2.72 (2.57-2.87) | 567.95 (431.86-732.96) | 1.9 (1.45-2.45) | -1.72 (-1.92 to -1.52) |
| Bosnia and Herzegovina | 172.56 (147.48-200.24) | 3.95 (3.38-4.58) | 164.81 (117.5-212.62) | 2.64 (1.88-3.42) | -1.44 (-1.59 to -1.3) |
| Brunei Darussalam | 1.89 (1.47-2.37) | 2.08 (1.62-2.62) | 2.4 (1.88-3.08) | 0.8 (0.63-1.02) | -2.99 (-3.18 to -2.81) |
| Burkina Faso | 41.24 (30.8-53.42) | 0.96 (0.73-1.23) | 103.51 (72.55-140.67) | 1.16 (0.83-1.56) | 1 (0.85 to 1.16) |
| Canada | 485.43 (447.4-524.96) | 1.5 (1.38-1.62) | 435.96 (389.22-483.78) | 0.59 (0.53-0.65) | -2.88 (-2.99 to -2.77) |
| Central African Republic | 25.41 (15.22-35.09) | 2.16 (1.4-2.89) | 39.35 (23.36-57.4) | 1.7 (1.1-2.38) | -0.82 (-0.87 to -0.77) |
| Commonwealth of Dominica | 1.3 (1.09-1.57) | 2.19 (1.83-2.62) | 1.99 (1.49-2.58) | 2.32 (1.75-3) | 0.26 (0.19 to 0.34) |
| Commonwealth of the Bahamas | 3.95 (3.58-4.32) | 2.55 (2.32-2.78) | 9.76 (7.78-12.12) | 2.36 (1.89-2.92) | -0.05 (-0.18 to 0.08) |
| Cook Islands | 0.06 (0.05-0.08) | 0.56 (0.44-0.68) | 0.09 (0.07-0.12) | 0.34 (0.26-0.46) | -1.79 (-2.19 to -1.38) |
| Czech Republic | 349.66 (306.56-400.84) | 2.6 (2.27-2.98) | 244.99 (195.16-303.34) | 1.2 (0.95-1.49) | -2.34 (-2.45 to -2.23) |
| Democratic People's Republic of Korea | 160.96 (114.88-216.89) | 0.98 (0.71-1.29) | 278.3 (199.23-367.64) | 0.83 (0.6-1.09) | -0.51 (-0.56 to -0.45) |
| Democratic Republic of Sao Tome and Principe | 0.43 (0.33-0.54) | 0.67 (0.52-0.85) | 0.8 (0.63-1.03) | 0.78 (0.62-1) | 0.74 (0.61 to 0.86) |
| Democratic Republic of Timor-Leste | 2.75 (1.88-3.95) | 1.05 (0.75-1.47) | 7.8 (5.53-10.96) | 0.94 (0.67-1.31) | -0.26 (-0.47 to -0.04) |
| Democratic Republic of the Congo | 231.79 (161.14-312.8) | 1.54 (1.11-2.01) | 477.59 (329.83-639.95) | 1.3 (0.91-1.72) | -0.52 (-0.68 to -0.36) |
| Democratic Socialist Republic of Sri Lanka | 94.46 (76.71-119.22) | 0.93 (0.75-1.16) | 250.93 (148-376.63) | 0.92 (0.56-1.38) | 1.12 (0.67 to 1.57) |
| Dominican Republic | 58.16 (46.16-73.22) | 1.65 (1.31-2.07) | 153.62 (111.09-206.12) | 1.55 (1.12-2.07) | 0.14 (0.01 to 0.27) |
| Eastern Republic of Uruguay | 186.84 (173.33-202.45) | 4.82 (4.47-5.21) | 131.25 (118.35-146.72) | 2.45 (2.21-2.74) | -2.22 (-2.34 to -2.1) |
| Federal Democratic Republic of Ethiopia | 323.95 (180.34-467.51) | 1.58 (0.91-2.27) | 387.31 (276.28-521.67) | 0.9 (0.65-1.2) | -2.11 (-2.26 to -1.96) |
| Federal Democratic Republic of Nepal | 277.15 (190.42-386.13) | 2.96 (2.05-4.11) | 503.24 (367.49-707.34) | 2.18 (1.6-3.05) | -0.93 (-1.19 to -0.67) |
| Federal Republic of Germany | 1947.5 (1802.25-2111) | 1.62 (1.5-1.75) | 1562.26 (1402.14-1732.83) | 0.83 (0.75-0.92) | -2.22 (-2.32 to -2.11) |
| Federal Republic of Nigeria | 608.62 (424.07-820.34) | 1.37 (0.97-1.83) | 976.51 (677.12-1328.63) | 1.11 (0.79-1.47) | -0.65 (-0.8 to -0.49) |
| Federal Republic of Somalia | 54.26 (34.83-79.66) | 2.13 (1.39-3.04) | 104.53 (65.02-153.74) | 1.6 (1.01-2.27) | -0.99 (-1.04 to -0.94) |
| Federated States of Micronesia | 0.38 (0.29-0.51) | 0.82 (0.62-1.1) | 0.46 (0.33-0.63) | 0.67 (0.49-0.91) | -0.83 (-0.9 to -0.76) |
| Federative Republic of Brazil | 2570.46 (2463.04-2672.4) | 2.84 (2.72-2.96) | 5497.44 (5144.06-5811.97) | 2.16 (2.02-2.29) | -0.84 (-0.94 to -0.75) |
| French Republic | 3003.9 (2755.65-3282.36) | 3.91 (3.61-4.26) | 1667.14 (1464.98-1868.91) | 1.27 (1.12-1.42) | -3.49 (-3.86 to -3.11) |
| Gabonese Republic | 11.47 (8.43-15.07) | 2.01 (1.49-2.63) | 16.7 (12-22.3) | 1.59 (1.15-2.1) | -0.82 (-0.86 to -0.77) |
| Georgia | 256.62 (235.86-277.95) | 3.96 (3.65-4.29) | 182.24 (157.54-207.67) | 3.08 (2.66-3.51) | -0.31 (-0.61 to -0.01) |
| Grand Duchy of Luxembourg | 13.94 (12.88-15.03) | 2.6 (2.4-2.8) | 9.84 (8.75-10.97) | 0.93 (0.83-1.04) | -3.25 (-3.43 to -3.06) |
| Greenland | 0.91 (0.73-1.11) | 2.51 (2.02-3.07) | 0.85 (0.67-1.1) | 1.18 (0.92-1.54) | -2.06 (-2.27 to -1.84) |
| Grenada | 1.07 (0.95-1.21) | 1.52 (1.35-1.72) | 1.34 (1.13-1.57) | 1.13 (0.96-1.31) | -0.56 (-1.15 to 0.03) |
| Guam | 0.37 (0.31-0.43) | 0.56 (0.46-0.65) | 0.5 (0.42-0.58) | 0.24 (0.21-0.28) | -2.75 (-3.05 to -2.45) |
| Hashemite Kingdom of Jordan | 19.75 (15.08-25.36) | 1.51 (1.14-1.95) | 48.65 (34.6-67.59) | 0.67 (0.48-0.93) | -3.25 (-3.57 to -2.93) |
| Hellenic Republic | 420.36 (394.26-444.06) | 2.74 (2.57-2.88) | 384.26 (348.58-414.88) | 1.65 (1.51-1.78) | -1.56 (-1.64 to -1.47) |
| Hungary | 629.49 (563.32-696.73) | 4.39 (3.92-4.87) | 482.71 (401.65-574.82) | 2.71 (2.25-3.23) | -1.94 (-2.08 to -1.8) |
| Independent State of Papua New Guinea | 8.55 (5.6-12.29) | 0.55 (0.37-0.8) | 21.66 (14.95-31.75) | 0.5 (0.35-0.74) | -0.32 (-0.36 to -0.27) |
| Independent State of Samoa | 0.24 (0.18-0.31) | 0.28 (0.22-0.36) | 0.35 (0.28-0.47) | 0.25 (0.19-0.33) | -0.55 (-0.63 to -0.46) |
| Ireland | 72.36 (65.81-79.37) | 1.8 (1.65-1.97) | 52.06 (45.95-58.3) | 0.66 (0.58-0.74) | -2.94 (-3.09 to -2.79) |
| Islamic Republic of Afghanistan | 224.21 (122.97-345.18) | 3.29 (1.85-4.93) | 266.31 (145.06-405.31) | 2.75 (1.58-3.94) | -0.67 (-0.78 to -0.55) |
| Islamic Republic of Iran | 673.86 (562.6-766.48) | 2.7 (2.24-3.1) | 1345.99 (1193.35-1501.56) | 1.8 (1.59-2) | -1.17 (-1.24 to -1.09) |
| Islamic Republic of Mauritania | 8.93 (6.97-11.29) | 0.92 (0.72-1.15) | 19.91 (13.9-27.91) | 0.97 (0.68-1.35) | 0.42 (0.2 to 0.64) |
| Islamic Republic of Pakistan | 2817.36 (2265.48-3485.56) | 5.04 (4.05-6.25) | 5616.89 (4105.95-7453.41) | 4.58 (3.35-6.04) | -0.61 (-0.85 to -0.37) |
| Jamaica | 20.83 (18.58-23.12) | 1.17 (1.04-1.3) | 39.33 (28.55-52.29) | 1.28 (0.93-1.7) | 0.4 (-0.02 to 0.82) |
| Japan | 981.14 (933.73-1012.55) | 0.58 (0.55-0.6) | 1092.46 (966.58-1164.8) | 0.25 (0.23-0.27) | -2.92 (-3.05 to -2.78) |
| Kingdom of Bahrain | 4.67 (3.79-5.91) | 3.09 (2.49-3.89) | 10.34 (7.44-15.04) | 1.39 (1.02-1.91) | -3.35 (-3.63 to -3.08) |
| Kingdom of Belgium | 407.11 (377.47-438.87) | 2.74 (2.54-2.96) | 220.62 (195.57-244.17) | 0.97 (0.87-1.08) | -3.37 (-3.49 to -3.25) |
| Kingdom of Bhutan | 6.11 (3.98-8.85) | 2.48 (1.64-3.6) | 11.25 (7.76-15.52) | 1.88 (1.31-2.6) | -0.91 (-1 to -0.81) |
| Kingdom of Cambodia | 79.6 (59.47-106.74) | 1.81 (1.38-2.4) | 181.24 (125.59-272.1) | 1.5 (1.06-2.21) | -0.69 (-0.83 to -0.55) |
| Kingdom of Denmark | 150.31 (138.59-163.24) | 1.95 (1.79-2.11) | 104.24 (93.54-115.55) | 0.88 (0.8-0.98) | -2.79 (-2.93 to -2.65) |
| Kingdom of Eswatini | 8.26 (5.28-11.38) | 2.79 (1.79-3.76) | 15.22 (9.01-22.11) | 2.53 (1.53-3.64) | -0.04 (-0.46 to 0.39) |
| Kingdom of Lesotho | 17.25 (13.04-22.31) | 2.02 (1.53-2.6) | 35.2 (23.92-48.27) | 3.08 (2.11-4.15) | 1.84 (1.53 to 2.16) |
| Kingdom of Morocco | 278.63 (215.29-349.35) | 1.98 (1.55-2.47) | 603.57 (427.29-782.87) | 1.76 (1.27-2.27) | -0.26 (-0.42 to -0.09) |
| Kingdom of Norway | 46.66 (43.56-49.23) | 0.69 (0.65-0.73) | 38.33 (34.38-41.57) | 0.37 (0.33-0.4) | -2.31 (-2.56 to -2.07) |
| Kingdom of Saudi Arabia | 51.53 (38.4-67.14) | 0.9 (0.68-1.17) | 118.65 (89.9-159.68) | 0.6 (0.47-0.78) | -1.62 (-1.76 to -1.48) |
| Kingdom of Spain | 2205.72 (1940.52-2459.97) | 4.16 (3.66-4.65) | 1414.76 (1242.07-1612.37) | 1.49 (1.31-1.71) | -3.52 (-3.63 to -3.41) |
| Kingdom of Sweden | 73.8 (67.03-80.38) | 0.48 (0.44-0.52) | 62.82 (53.83-72.07) | 0.27 (0.23-0.31) | -1.6 (-1.84 to -1.37) |
| Kingdom of Thailand | 694.33 (564.79-828.62) | 2.02 (1.64-2.38) | 1346.13 (994.28-1756.76) | 1.23 (0.91-1.6) | -1.98 (-2.1 to -1.85) |
| Kingdom of Tonga | 0.29 (0.21-0.4) | 0.57 (0.41-0.8) | 0.39 (0.28-0.54) | 0.5 (0.36-0.68) | -0.47 (-0.69 to -0.24) |
| Kingdom of the Netherlands | 267.55 (249.01-288.33) | 1.36 (1.27-1.47) | 224.33 (201.2-245.71) | 0.63 (0.56-0.68) | -2.71 (-2.85 to -2.58) |
| Kyrgyz Republic | 61.32 (51.46-72.41) | 1.97 (1.66-2.33) | 37.7 (28.18-47.86) | 0.74 (0.55-0.93) | -3.16 (-3.34 to -2.97) |
| Lao People's Democratic Republic | 36.46 (25.64-50.49) | 1.76 (1.25-2.4) | 50.95 (34.87-74.74) | 1.15 (0.8-1.68) | -1.42 (-1.52 to -1.32) |
| Lebanese Republic | 63.57 (43.99-85.29) | 2.96 (2.1-3.89) | 110.8 (89.18-137.53) | 1.82 (1.47-2.26) | -1.23 (-1.41 to -1.04) |
| Malaysia | 140.86 (107.43-174.51) | 1.58 (1.2-1.97) | 345.83 (272.05-415.5) | 1.25 (0.98-1.5) | -0.99 (-1.19 to -0.8) |
| Mongolia | 15.67 (11.4-20.4) | 1.46 (1.07-1.88) | 19.6 (14.28-26.96) | 0.79 (0.58-1.06) | -2.34 (-2.6 to -2.07) |
| Montenegro | 33.05 (26.28-41.44) | 5.12 (4.08-6.43) | 45.88 (35.37-60.22) | 4.67 (3.61-6.1) | -0.4 (-0.64 to -0.17) |
| New Zealand | 35.36 (31.99-38.93) | 0.9 (0.81-0.98) | 32.22 (27.83-36.5) | 0.37 (0.32-0.42) | -2.84 (-3.15 to -2.52) |
| North Macedonia | 79.85 (65.56-99.32) | 4.15 (3.39-5.16) | 108.48 (82.1-141.24) | 3.2 (2.45-4.13) | -0.97 (-1.23 to -0.71) |
| Northern Mariana Islands | 0.16 (0.12-0.22) | 1.14 (0.84-1.45) | 0.3 (0.24-0.36) | 0.66 (0.54-0.8) | -2.36 (-2.72 to -2.01) |
| Palestine | 11.73 (8.69-15.33) | 1.43 (1.06-1.88) | 21.42 (17.17-26.48) | 0.91 (0.75-1.11) | -1.75 (-2.02 to -1.48) |
| People's Democratic Republic of Algeria | 212.32 (162.39-281.74) | 1.96 (1.51-2.54) | 395.55 (291.36-526.25) | 1.21 (0.9-1.59) | -1.46 (-1.63 to -1.28) |
| People's Republic of Bangladesh | 1806.24 (1410.68-2264.7) | 3.86 (3-4.82) | 2971.74 (2134.87-4039.15) | 2.17 (1.57-2.94) | -1.95 (-2.1 to -1.81) |
| People's Republic of China | 12869.79 (10565.15-15142.78) | 1.59 (1.32-1.86) | 19799.45 (15579.57-25023.24) | 0.94 (0.74-1.17) | -1.74 (-1.83 to -1.65) |
| Plurinational State of Bolivia | 49.54 (35.63-65.19) | 1.62 (1.16-2.11) | 93.25 (64.05-131.43) | 1.07 (0.75-1.5) | -1.31 (-1.41 to -1.21) |
| Portuguese Republic | 474.02 (427.53-522.76) | 3.46 (3.15-3.82) | 355.84 (314.24-405.27) | 1.57 (1.39-1.78) | -2.62 (-2.77 to -2.48) |
| Principality of Andorra | 0.79 (0.54-1.19) | 1.36 (0.93-2.05) | 1.01 (0.63-1.51) | 0.65 (0.41-0.97) | -2.23 (-2.47 to -2) |
| Principality of Monaco | 3.51 (2.53-4.71) | 5.44 (3.9-7.29) | 2.99 (2.31-3.87) | 3.28 (2.5-4.39) | -1.71 (-1.8 to -1.62) |
| Puerto Rico | 83.61 (74.74-92.31) | 2.31 (2.06-2.55) | 58.86 (47.53-71.77) | 0.85 (0.68-1.04) | -3.03 (-3.32 to -2.74) |
| Republic of Albania | 66.53 (53.3-81.72) | 3.22 (2.57-3.95) | 89.77 (62.38-128.67) | 2.05 (1.44-2.93) | -1.18 (-1.39 to -0.97) |
| Republic of Angola | 75.31 (50.91-105.54) | 1.93 (1.35-2.67) | 176.59 (132.81-230.15) | 1.51 (1.16-1.97) | -0.75 (-0.87 to -0.63) |
| Republic of Armenia | 116.82 (110.97-121.61) | 4.04 (3.85-4.21) | 74.94 (66.91-84.09) | 1.7 (1.52-1.91) | -2.83 (-3.09 to -2.56) |
| Republic of Austria | 209.58 (193.84-228.24) | 1.87 (1.72-2.04) | 143.56 (126.13-159.68) | 0.81 (0.71-0.89) | -2.63 (-2.84 to -2.43) |
| Republic of Azerbaijan | 156.73 (136.08-181.26) | 2.96 (2.55-3.44) | 191.5 (141.47-272.97) | 1.76 (1.3-2.46) | -1.72 (-1.86 to -1.58) |
| Republic of Belarus | 455.4 (398.82-523.67) | 3.45 (3.03-3.95) | 347.3 (261.96-443.52) | 2.17 (1.64-2.76) | -2.69 (-3.1 to -2.28) |
| Republic of Benin | 18.66 (14.79-22.87) | 0.96 (0.77-1.17) | 49.89 (36.61-65.53) | 1 (0.75-1.3) | 0.42 (0.31 to 0.54) |
| Republic of Botswana | 14.4 (10.54-18.41) | 2.5 (1.85-3.14) | 25.58 (17.92-39.81) | 1.69 (1.21-2.54) | -1.63 (-1.96 to -1.3) |
| Republic of Bulgaria | 376.15 (322.33-437.56) | 3.07 (2.61-3.54) | 420.63 (335.54-518.13) | 3.13 (2.49-3.85) | 0.33 (0.1 to 0.57) |
| Republic of Burundi | 53.19 (34.2-74.08) | 2.25 (1.45-3.11) | 70.05 (48.6-101.01) | 1.4 (0.98-1.99) | -1.89 (-2.12 to -1.66) |
| Republic of Cabo Verde | 3.57 (2.83-4.46) | 1.54 (1.21-1.91) | 6.31 (4.31-8.06) | 1.43 (0.98-1.8) | -0.52 (-1.02 to -0.02) |
| Republic of Cameroon | 45.16 (35.38-58.63) | 1.03 (0.81-1.32) | 151.48 (99.25-218.97) | 1.23 (0.82-1.75) | 0.85 (0.7 to 1.01) |
| Republic of Chad | 22.19 (17.07-28.54) | 0.8 (0.62-1.03) | 74.13 (52.14-99.34) | 1.34 (0.95-1.78) | 1.96 (1.78 to 2.14) |
| Republic of Chile | 147.47 (133.88-163.7) | 1.47 (1.33-1.63) | 153.38 (135.61-170.96) | 0.59 (0.52-0.66) | -2.79 (-2.92 to -2.66) |
| Republic of Colombia | 416.31 (382.59-447.35) | 2.48 (2.29-2.67) | 482.11 (392.34-584.61) | 0.88 (0.71-1.06) | -3.99 (-4.18 to -3.8) |
| Republic of Costa Rica | 29.48 (26.65-32.28) | 1.74 (1.57-1.9) | 39.31 (33.93-45.91) | 0.72 (0.62-0.84) | -2.88 (-3.09 to -2.67) |
| Republic of Croatia | 283.42 (256.06-312.18) | 4.49 (4.06-4.95) | 177.47 (149.62-206.57) | 2.06 (1.74-2.41) | -2.53 (-2.67 to -2.39) |
| Republic of Cuba | 431.22 (389.45-480.29) | 4.24 (3.83-4.71) | 921.23 (780.07-1113.76) | 4.65 (3.93-5.61) | 0.4 (0.27 to 0.53) |
| Republic of Cyprus | 11.52 (9.21-14.4) | 1.59 (1.26-1.97) | 15.34 (12.21-19.66) | 0.74 (0.59-0.94) | -2.4 (-2.46 to -2.34) |
| Republic of C么te d'Ivoire | 51.8 (40.44-66.61) | 1.28 (1.01-1.62) | 126.67 (87.51-195.5) | 1.11 (0.8-1.66) | -0.69 (-0.87 to -0.51) |
| Republic of Djibouti | 2.85 (1.95-4.33) | 1.98 (1.39-2.92) | 11.34 (7.66-16.04) | 1.7 (1.17-2.35) | -0.62 (-0.73 to -0.5) |
| Republic of Ecuador | 53.6 (49.51-58.25) | 1.06 (0.97-1.15) | 87.34 (68.44-111.73) | 0.54 (0.43-0.69) | -2.08 (-2.34 to -1.81) |
| Republic of El Salvador | 27.89 (23.79-32.63) | 0.96 (0.82-1.13) | 41.74 (32.55-53.22) | 0.67 (0.52-0.86) | -1.52 (-1.72 to -1.32) |
| Republic of Equatorial Guinea | 3.78 (2.3-5.45) | 1.92 (1.23-2.72) | 6 (3.55-8.84) | 1.22 (0.73-1.78) | -1.59 (-1.93 to -1.24) |
| Republic of Estonia | 51.44 (45.62-57.96) | 2.5 (2.23-2.81) | 29.38 (23.99-35.19) | 1.18 (0.96-1.43) | -3.07 (-3.39 to -2.74) |
| Republic of Fiji | 2.37 (1.85-3.04) | 0.72 (0.56-0.93) | 4.85 (3.5-6.39) | 0.68 (0.5-0.89) | -0.09 (-0.22 to 0.04) |
| Republic of Finland | 56.09 (51.17-61.06) | 0.8 (0.73-0.88) | 49.09 (43.48-54.74) | 0.4 (0.36-0.45) | -2.25 (-2.53 to -1.98) |
| Republic of Ghana | 48.76 (37.75-64.39) | 0.8 (0.64-1.05) | 176.35 (125.15-230.75) | 1.09 (0.78-1.41) | 1.63 (1.37 to 1.88) |
| Republic of Guatemala | 51.29 (48.95-53.88) | 1.68 (1.59-1.76) | 52.57 (44.93-61.56) | 0.5 (0.42-0.58) | -4.1 (-4.31 to -3.9) |
| Republic of Guinea | 27.08 (20.67-34.75) | 0.84 (0.64-1.07) | 67.12 (49.1-86.55) | 1.23 (0.91-1.58) | 1.8 (1.61 to 1.98) |
| Republic of Guinea-Bissau | 5.53 (3.49-7.81) | 1.4 (0.91-1.94) | 10.89 (7.28-14.92) | 1.52 (1.04-2.02) | 0.71 (0.56 to 0.87) |
| Republic of Guyana | 4.48 (3.89-5.21) | 1.2 (1.04-1.4) | 7.1 (5.28-9.33) | 1.07 (0.8-1.4) | 0.22 (-0.01 to 0.45) |
| Republic of Haiti | 90.44 (61.1-119.69) | 2.83 (1.96-3.71) | 159.43 (102.76-224.26) | 2.31 (1.49-3.24) | -0.49 (-0.57 to -0.41) |
| Republic of Honduras | 23.04 (18.94-28.75) | 1.18 (0.97-1.46) | 86.81 (64.39-111.85) | 1.45 (1.07-1.87) | 0.94 (0.8 to 1.07) |
| Republic of Iceland | 2.26 (2.03-2.48) | 0.81 (0.73-0.89) | 2.07 (1.79-2.34) | 0.36 (0.31-0.41) | -2.72 (-2.9 to -2.54) |
| Republic of India | 14101.91 (11465.35-16898.08) | 2.95 (2.38-3.53) | 28330.04 (24663.74-32828.51) | 2.35 (2.05-2.72) | -0.84 (-0.98 to -0.7) |
| Republic of Indonesia | 1032.34 (773.63-1253.41) | 1.08 (0.79-1.32) | 2366.43 (1668.49-3118.31) | 1.03 (0.72-1.34) | -0.15 (-0.2 to -0.11) |
| Republic of Iraq | 193.78 (148.26-246.59) | 2.46 (1.88-3.13) | 463.14 (329.58-617.52) | 2.02 (1.45-2.67) | -0.99 (-1.09 to -0.88) |
| Republic of Italy | 2599.43 (2475.32-2720.74) | 2.93 (2.79-3.06) | 1641.07 (1486.56-1774.47) | 1.11 (1.02-1.19) | -3.13 (-3.25 to -3.02) |
| Republic of Kazakhstan | 426.85 (393.26-459.78) | 3.19 (2.95-3.45) | 221.54 (190.58-253.59) | 1.15 (1-1.32) | -3.89 (-4.2 to -3.59) |
| Republic of Kenya | 95.11 (69.03-129.38) | 1.15 (0.84-1.57) | 304.59 (224.39-393.74) | 1.29 (0.95-1.64) | 0.46 (0.26 to 0.66) |
| Republic of Kiribati | 0.07 (0.06-0.09) | 0.21 (0.16-0.27) | 0.15 (0.11-0.21) | 0.22 (0.16-0.3) | 0.09 (-0.01 to 0.19) |
| Republic of Korea | 649.14 (459.88-822.69) | 2.3 (1.63-2.91) | 504.52 (370.83-647.75) | 0.53 (0.39-0.68) | -5.65 (-6 to -5.29) |
| Republic of Latvia | 110.01 (99.02-123.08) | 3.07 (2.77-3.45) | 61.87 (51.47-74.13) | 1.72 (1.42-2.07) | -2.11 (-2.3 to -1.91) |
| Republic of Liberia | 10.24 (7.39-14.76) | 0.92 (0.67-1.33) | 20.63 (13.92-29.55) | 1.03 (0.72-1.45) | 0.57 (0.42 to 0.72) |
| Republic of Lithuania | 152.63 (133.2-174.67) | 3.38 (2.95-3.87) | 110.75 (87.64-135.6) | 2.11 (1.67-2.6) | -1.67 (-1.91 to -1.44) |
| Republic of Madagascar | 82.14 (60.56-102.99) | 1.59 (1.18-2) | 123.79 (87.23-166.72) | 1.05 (0.75-1.4) | -1.39 (-1.51 to -1.26) |
| Republic of Malawi | 22.59 (17.31-30.16) | 0.58 (0.44-0.77) | 43.98 (33.05-61.45) | 0.57 (0.43-0.78) | -0.28 (-0.47 to -0.09) |
| Republic of Maldives | 0.99 (0.71-1.27) | 1.26 (0.92-1.58) | 1.51 (1.12-1.96) | 0.49 (0.37-0.63) | -3.38 (-3.53 to -3.23) |
| Republic of Mali | 42.23 (35.12-51.11) | 1.06 (0.9-1.27) | 84.32 (62-116.12) | 0.96 (0.72-1.31) | -0.02 (-0.16 to 0.13) |
| Republic of Malta | 8.47 (7.41-9.57) | 1.98 (1.73-2.24) | 7.08 (6.04-8.23) | 0.78 (0.66-0.9) | -3.12 (-3.21 to -3.04) |
| Republic of Mauritius | 14.82 (13.9-15.98) | 2.04 (1.92-2.21) | 27.22 (24.78-29.31) | 1.46 (1.33-1.58) | -0.83 (-1.62 to -0.03) |
| Republic of Moldova | 158.91 (149.43-169.04) | 3.48 (3.27-3.68) | 146.54 (129.72-166.92) | 2.44 (2.16-2.77) | -1.19 (-1.47 to -0.9) |
| Republic of Mozambique | 112.83 (82.83-150.49) | 1.86 (1.38-2.44) | 210.96 (153.7-277.06) | 1.8 (1.32-2.32) | 0.28 (0.14 to 0.42) |
| Republic of Namibia | 16.54 (13.31-20.37) | 2.48 (2.01-3.03) | 34.88 (25.43-47.07) | 2.42 (1.8-3.2) | -0.24 (-0.52 to 0.05) |
| Republic of Nauru | 0.04 (0.03-0.06) | 1.09 (0.74-1.44) | 0.04 (0.03-0.06) | 0.76 (0.52-1.04) | -1.28 (-1.5 to -1.05) |
| Republic of Nicaragua | 17.72 (14.34-21.5) | 1.23 (0.99-1.49) | 35.51 (27.65-46.39) | 0.75 (0.59-0.98) | -1.64 (-1.82 to -1.46) |
| Republic of Niue | 0.01 (0.01-0.02) | 0.53 (0.39-0.69) | 0.01 (0.01-0.01) | 0.49 (0.37-0.64) | -0.48 (-0.64 to -0.31) |
| Republic of Palau | 0.05 (0.04-0.07) | 0.58 (0.43-0.76) | 0.09 (0.07-0.12) | 0.45 (0.33-0.61) | -0.9 (-1 to -0.79) |
| Republic of Panama | 23.18 (21.58-24.63) | 1.63 (1.51-1.73) | 31.99 (24.99-38.9) | 0.72 (0.57-0.88) | -2.84 (-3.03 to -2.64) |
| Republic of Paraguay | 28.84 (22.48-37.11) | 1.31 (1.02-1.68) | 95.67 (66.24-130.92) | 1.64 (1.14-2.24) | 0.91 (0.81 to 1) |
| Republic of Peru | 133.81 (107.57-162.57) | 1.17 (0.94-1.42) | 186.78 (132.49-258.46) | 0.57 (0.4-0.78) | -2.77 (-3.14 to -2.4) |
| Republic of Poland | 1838.52 (1768.12-1911.02) | 4.2 (4.05-4.37) | 1771.71 (1593.69-1945.45) | 2.5 (2.25-2.75) | -1.9 (-2.05 to -1.74) |
| Republic of Rwanda | 76.64 (54.37-99.31) | 2.62 (1.88-3.41) | 93.13 (62.87-137.93) | 1.45 (0.99-2.11) | -2.81 (-3.15 to -2.48) |
| Republic of San Marino | 0.76 (0.59-0.95) | 2.2 (1.71-2.74) | 0.6 (0.37-0.87) | 0.82 (0.5-1.21) | -2.35 (-2.67 to -2.02) |
| Republic of Senegal | 32 (25.29-39.12) | 1.01 (0.8-1.23) | 86.04 (62.26-112.77) | 1.15 (0.83-1.49) | 0.78 (0.58 to 0.97) |
| Republic of Serbia | 477.45 (343.4-674.76) | 4.13 (2.98-5.76) | 449.06 (310.56-616.69) | 2.79 (1.93-3.86) | -1.43 (-1.63 to -1.23) |
| Republic of Seychelles | 2.92 (2.5-3.45) | 5.19 (4.43-6.12) | 4.59 (3.73-5.51) | 3.94 (3.22-4.73) | -0.23 (-0.53 to 0.06) |
| Republic of Sierra Leone | 19.58 (14.67-25.4) | 0.98 (0.74-1.27) | 39.51 (28.09-55.71) | 1.08 (0.78-1.5) | 0.78 (0.59 to 0.97) |
| Republic of Singapore | 32.71 (29.49-35.92) | 1.55 (1.4-1.7) | 34.71 (30.04-39.2) | 0.41 (0.36-0.47) | -4.9 (-5.31 to -4.5) |
| Republic of Slovenia | 66.37 (59.93-74.26) | 2.67 (2.42-2.99) | 51.13 (42.52-59.8) | 1.21 (1.01-1.42) | -2.78 (-2.98 to -2.58) |
| Republic of South Africa | 396.81 (322.26-540.78) | 1.87 (1.52-2.58) | 746.49 (654.95-841.64) | 1.56 (1.38-1.76) | -0.89 (-1.21 to -0.56) |
| Republic of South Sudan | 51.37 (35.62-69.3) | 1.98 (1.4-2.66) | 61.12 (39.36-90.29) | 1.53 (1-2.2) | -1.06 (-1.23 to -0.89) |
| Republic of Sudan | 218.43 (134.48-342.88) | 2.4 (1.48-3.87) | 347.31 (216.17-513.11) | 1.84 (1.17-2.71) | -0.95 (-0.99 to -0.91) |
| Republic of Suriname | 2.24 (1.84-2.66) | 0.89 (0.73-1.05) | 5.05 (3.58-7) | 0.78 (0.55-1.09) | -0.2 (-0.4 to 0) |
| Republic of Tajikistan | 44.77 (33.36-56.39) | 1.58 (1.18-1.99) | 52.99 (35.77-74.66) | 0.86 (0.6-1.2) | -2.05 (-2.3 to -1.8) |
| Republic of Trinidad and Tobago | 11.83 (11.03-12.64) | 1.43 (1.34-1.53) | 22.43 (17.07-28.77) | 1.14 (0.87-1.47) | -0.85 (-1 to -0.71) |
| Republic of Tunisia | 133.04 (102.45-169.4) | 2.72 (2.11-3.42) | 267.79 (188.95-376.13) | 2.02 (1.44-2.83) | -1.15 (-1.23 to -1.08) |
| Republic of Turkey | 1256.3 (907.32-1693.43) | 3.68 (2.66-5) | 1649.48 (1285.88-2109.45) | 1.77 (1.39-2.27) | -2.63 (-2.84 to -2.43) |
| Republic of Uganda | 143.88 (108.31-184.43) | 2.22 (1.69-2.82) | 262.85 (181.95-368.87) | 1.74 (1.25-2.42) | -1.46 (-1.72 to -1.2) |
| Republic of Uzbekistan | 249.66 (219.11-284.18) | 2.1 (1.86-2.38) | 174.73 (138.92-219.78) | 0.65 (0.52-0.8) | -3.52 (-4.18 to -2.86) |
| Republic of Vanuatu | 0.33 (0.21-0.46) | 0.6 (0.41-0.83) | 0.76 (0.53-1.05) | 0.49 (0.35-0.66) | -0.84 (-0.94 to -0.74) |
| Republic of Yemen | 131.49 (81.93-186.79) | 2.67 (1.67-3.8) | 325 (200.19-473.3) | 2.39 (1.49-3.44) | -0.43 (-0.49 to -0.38) |
| Republic of Zambia | 60.55 (45.22-76.52) | 2.1 (1.57-2.65) | 151.21 (74.83-345.17) | 2.03 (1.08-4.42) | -0.32 (-0.46 to -0.18) |
| Republic of Zimbabwe | 84.49 (64.68-105.59) | 2.06 (1.6-2.54) | 164.59 (124.52-221.38) | 2.22 (1.73-2.9) | 0.39 (0.14 to 0.65) |
| Republic of the Congo | 22.32 (14.26-29.27) | 2.09 (1.37-2.69) | 41.93 (30.8-55.84) | 1.58 (1.19-2.07) | -1.11 (-1.26 to -0.96) |
| Republic of the Gambia | 1.74 (1.33-2.27) | 0.49 (0.38-0.63) | 4.47 (3.32-5.79) | 0.46 (0.34-0.59) | -0.35 (-0.49 to -0.2) |
| Republic of the Marshall Islands | 0.12 (0.09-0.17) | 0.79 (0.56-1.08) | 0.23 (0.15-0.33) | 0.78 (0.51-1.11) | 0.06 (-0.07 to 0.2) |
| Republic of the Niger | 23.65 (17.27-31.24) | 0.87 (0.64-1.14) | 73.41 (48.96-103.96) | 0.96 (0.66-1.33) | 0.71 (0.56 to 0.86) |
| Republic of the Philippines | 269.29 (226.03-329.5) | 0.95 (0.79-1.16) | 677.77 (546.06-816.99) | 0.83 (0.68-1) | -0.39 (-0.47 to -0.31) |
| Republic of the Union of Myanmar | 374.44 (237.47-514.29) | 1.64 (1.08-2.21) | 429.61 (303.73-607.3) | 0.91 (0.65-1.28) | -2.11 (-2.22 to -2.01) |
| Romania | 911.51 (803-1019.92) | 3.19 (2.81-3.57) | 1049.16 (894-1222.24) | 3.05 (2.59-3.56) | -0.35 (-0.53 to -0.17) |
| Russian Federation | 6222.56 (6045.66-6380.7) | 3.35 (3.25-3.43) | 4013.73 (3572.38-4418.24) | 1.67 (1.49-1.84) | -2.93 (-3.18 to -2.68) |
| Saint Kitts and Nevis | 0.59 (0.54-0.65) | 1.6 (1.45-1.74) | 1.11 (0.9-1.34) | 1.56 (1.28-1.85) | 0.31 (0.06 to 0.55) |
| Saint Lucia | 2.07 (1.94-2.22) | 2.43 (2.28-2.6) | 4.99 (4.05-6.07) | 2.06 (1.67-2.5) | -0.68 (-0.9 to -0.45) |
| Saint Vincent and the Grenadines | 1.69 (1.53-1.86) | 2.37 (2.14-2.6) | 3.85 (3.35-4.42) | 2.67 (2.33-3.06) | 0.39 (0.21 to 0.58) |
| Slovak Republic | 230.28 (174.93-298.79) | 3.91 (2.99-5.05) | 189.22 (137.13-250.09) | 2.02 (1.46-2.67) | -2.17 (-2.25 to -2.1) |
| Socialist Republic of Viet Nam | 514.33 (405.04-669.44) | 1.29 (1.02-1.66) | 1354.66 (994.42-1811.08) | 1.34 (0.99-1.74) | 0.13 (0.06 to 0.2) |
| Solomon Islands | 1 (0.6-1.45) | 0.84 (0.54-1.18) | 2.22 (1.58-3.05) | 0.7 (0.5-0.95) | -0.61 (-0.67 to -0.55) |
| State of Eritrea | 26.63 (18.41-36.94) | 2.05 (1.44-2.79) | 45.92 (31.53-63.18) | 1.53 (1.09-2.07) | -1.17 (-1.26 to -1.08) |
| State of Israel | 57.47 (51.85-64.05) | 1.22 (1.1-1.35) | 83.42 (73.16-94.84) | 0.68 (0.6-0.78) | -2.31 (-2.52 to -2.1) |
| State of Kuwait | 9.12 (8.06-10.16) | 1.49 (1.32-1.66) | 10.38 (8.47-12.92) | 0.39 (0.32-0.48) | -3.5 (-4.1 to -2.91) |
| State of Libya | 59.69 (43.54-82.03) | 3.2 (2.34-4.37) | 157.8 (113.4-215.13) | 2.99 (2.17-4.04) | 0.01 (-0.14 to 0.15) |
| State of Qatar | 2.56 (1.92-3.38) | 2.81 (2.07-3.75) | 10.53 (7.29-14.61) | 1.31 (0.91-1.84) | -2.64 (-3.3 to -1.98) |
| Sultanate of Oman | 5.49 (3.86-7.61) | 0.81 (0.58-1.11) | 8.51 (6.39-11.37) | 0.44 (0.34-0.58) | -1.74 (-1.91 to -1.56) |
| Swiss Confederation | 139.57 (128.99-150.93) | 1.4 (1.29-1.51) | 96.18 (84.38-107.18) | 0.53 (0.47-0.59) | -2.86 (-3.06 to -2.66) |
| Syrian Arab Republic | 75.62 (57.89-99.11) | 1.5 (1.15-1.98) | 141.94 (103.15-200.85) | 1.16 (0.86-1.59) | -1.13 (-1.3 to -0.96) |
| Taiwan (Province of China) | 186.11 (174.55-200.55) | 1.17 (1.09-1.26) | 250.34 (224.31-276.11) | 0.59 (0.53-0.65) | -2.62 (-2.85 to -2.38) |
| Togolese Republic | 12.11 (9.08-15.59) | 1.01 (0.77-1.31) | 47.21 (33.44-63.94) | 1.23 (0.88-1.67) | 0.89 (0.77 to 1.02) |
| Tokelau | 0.01 (0.01-0.01) | 0.64 (0.46-0.92) | 0.01 (0-0.01) | 0.47 (0.33-0.66) | -1.2 (-1.33 to -1.08) |
| Turkmenistan | 49.12 (45.57-52.74) | 2.45 (2.27-2.63) | 43.86 (33.68-57.64) | 1.03 (0.79-1.34) | -3.2 (-3.46 to -2.94) |
| Tuvalu | 0.04 (0.03-0.06) | 0.68 (0.52-0.86) | 0.06 (0.04-0.07) | 0.58 (0.45-0.74) | -0.52 (-0.62 to -0.41) |
| Ukraine | 2452.9 (2213.98-2715.14) | 3.43 (3.08-3.82) | 1333.47 (871.52-1879.05) | 1.79 (1.16-2.52) | -2.74 (-2.96 to -2.52) |
| Union of the Comoros | 3.65 (2.44-4.88) | 1.8 (1.23-2.39) | 6.44 (4.68-9) | 1.3 (0.95-1.8) | -1.36 (-1.54 to -1.17) |
| United Arab Emirates | 7.65 (4.73-11) | 1.72 (1.09-2.39) | 34.58 (24.47-46.73) | 1.08 (0.8-1.37) | -0.31 (-0.78 to 0.16) |
| United Kingdom of Great Britain and Northern Ireland | 1047.42 (1015.81-1070.61) | 1.17 (1.14-1.19) | 937.78 (877.78-976.36) | 0.71 (0.67-0.74) | -1.75 (-1.89 to -1.61) |
| United Mexican States | 804.09 (779.97-825.78) | 2.07 (2-2.13) | 971.99 (830.03-1122.81) | 0.79 (0.68-0.91) | -3.43 (-3.57 to -3.28) |
| United Republic of Tanzania | 207.19 (147.88-286.51) | 1.87 (1.34-2.53) | 321.3 (222.93-489.72) | 1.23 (0.87-1.81) | -1.65 (-1.76 to -1.55) |
| United States Virgin Islands | 1.42 (1.14-1.83) | 1.68 (1.35-2.15) | 1.84 (1.3-2.5) | 1.02 (0.73-1.37) | -1.55 (-1.77 to -1.33) |
| United States of America | 4198.67 (4024.92-4304.45) | 1.35 (1.29-1.38) | 4620.32 (4339.97-4835.98) | 0.78 (0.74-0.82) | -2.06 (-2.13 to -1.98) |

| **TableS4: DALYs and age standardized DALYs of larynx cancer in 1990 and 2021 and its trends** | | | | | |
| --- | --- | --- | --- | --- | --- |
| Location | Number 1990 | ASR 1990 | Number 2021 | ASR 2021 | EAPC_95%CI |
| American Samoa | 6.21 (4.75-7.82) | 26.37 (19.86-33.5) | 6.12 (4.83-7.77) | 12.57 (10-15.91) | -3.01 (-3.32 to -2.71) |
| Antigua and Barbuda | 22.53 (20.52-24.4) | 44.91 (40.91-48.58) | 42.11 (37.6-47.62) | 37.46 (33.55-42.31) | -0.53 (-0.82 to -0.24) |
| Arab Republic of Egypt | 9145.63 (7744.93-11378.06) | 29.76 (25.15-37.62) | 18860.33 (14351.88-24382.01) | 26.93 (20.83-34.42) | -0.02 (-0.23 to 0.19) |
| Argentine Republic | 31009.84 (28472.53-33962.03) | 94.91 (87.11-103.87) | 21985.98 (19853.64-24311.08) | 40.28 (36.34-44.57) | -2.84 (-3.02 to -2.65) |
| Australia | 6245.77 (5641.4-6935.83) | 32.55 (29.35-36.14) | 5114.11 (4514.15-5727.86) | 12.05 (10.65-13.56) | -3.25 (-3.35 to -3.16) |
| Barbados | 95.85 (87.8-104.73) | 36.04 (33.15-39.18) | 181.97 (141.94-231.06) | 35.58 (27.68-45.31) | 0 (-0.17 to 0.17) |
| Belize | 29.98 (27.82-32.23) | 32.01 (29.68-34.43) | 119.5 (101.85-138.87) | 37.28 (31.92-43.41) | 0.67 (0.12 to 1.22) |
| Bermuda | 37.62 (34.19-41.59) | 59.14 (53.82-65.31) | 43.05 (35.01-53.58) | 33.63 (27.19-41.97) | -1.54 (-1.74 to -1.34) |
| Bolivarian Republic of Venezuela | 6632.83 (6316.28-6958.8) | 66.3 (62.8-69.66) | 14321.56 (10736.3-18591.44) | 46.26 (34.75-59.96) | -1.78 (-2 to -1.57) |
| Bosnia and Herzegovina | 5219.01 (4457.91-6096.09) | 112.29 (96.14-130.71) | 4252.62 (3026.84-5509.99) | 71.25 (50.45-92.44) | -1.63 (-1.77 to -1.49) |
| Brunei Darussalam | 48.51 (37.8-60.22) | 47.15 (36.77-59.4) | 64.27 (49.87-84.09) | 17.89 (13.98-22.99) | -3.14 (-3.32 to -2.96) |
| Burkina Faso | 1159.8 (856.89-1525.32) | 25.03 (18.53-32.65) | 2877.78 (1984.74-3973.61) | 29.16 (20.2-39.87) | 0.86 (0.71 to 1.02) |
| Canada | 12646.78 (11628.46-13700.88) | 39.92 (36.62-43.24) | 9899.35 (8827.69-11033.62) | 14.45 (12.9-16.16) | -3.17 (-3.28 to -3.06) |
| Central African Republic | 781.64 (453.19-1091.7) | 59.15 (35.87-81.56) | 1254.71 (719.99-1875.9) | 46.5 (28.43-67.22) | -0.84 (-0.9 to -0.79) |
| Commonwealth of Dominica | 31.17 (26.24-37.2) | 53.57 (45.17-63.88) | 51.6 (38.74-67.02) | 59.07 (44.46-76.51) | 0.46 (0.38 to 0.53) |
| Commonwealth of the Bahamas | 116.3 (104.97-128) | 71.04 (64.15-78.3) | 276.05 (218.79-346.67) | 62.37 (49.65-77.98) | -0.2 (-0.34 to -0.06) |
| Cook Islands | 1.58 (1.22-1.96) | 12.61 (9.81-15.6) | 2.01 (1.54-2.77) | 7.8 (5.98-10.75) | -1.67 (-2.03 to -1.3) |
| Czech Republic | 10142.97 (8841.91-11644.66) | 77.46 (67.37-88.74) | 6239.88 (4950.16-7750.61) | 32.96 (26.05-41.13) | -2.62 (-2.73 to -2.51) |
| Democratic People's Republic of Korea | 4868.19 (3421.93-6608.43) | 26.8 (19.1-36.06) | 7900.92 (5395.79-10694.65) | 22.83 (15.75-30.45) | -0.53 (-0.59 to -0.47) |
| Democratic Republic of Sao Tome and Principe | 10.87 (8.32-13.66) | 16.16 (12.42-20.29) | 21 (16.65-27.07) | 18.22 (14.46-23.62) | 0.53 (0.43 to 0.62) |
| Democratic Republic of Timor-Leste | 84.06 (56.43-122.6) | 26.54 (18.33-37.84) | 204.05 (142.02-292.78) | 23 (15.98-32.85) | -0.35 (-0.6 to -0.1) |
| Democratic Republic of the Congo | 6791.92 (4673.84-9264.37) | 39.38 (27.77-52.82) | 14436.13 (9899.2-19845.4) | 34.23 (23.7-45.8) | -0.43 (-0.58 to -0.28) |
| Democratic Socialist Republic of Sri Lanka | 2621.93 (2108.07-3318.6) | 22.85 (18.48-28.95) | 6482.58 (3782.95-9926.71) | 23.29 (13.7-35.5) | 1.1 (0.67 to 1.53) |
| Dominican Republic | 1577.59 (1260.46-2002.39) | 40.62 (32.35-51.59) | 4024.84 (2859.14-5457.51) | 39.42 (27.94-53.4) | 0.24 (0.13 to 0.36) |
| Eastern Republic of Uruguay | 5014.81 (4621.67-5457.58) | 132.95 (122.51-144.48) | 3195.68 (2897.88-3574.5) | 64.39 (58.36-72.19) | -2.4 (-2.53 to -2.27) |
| Federal Democratic Republic of Ethiopia | 9820.23 (5292.86-14193.32) | 43.39 (24.01-62.62) | 11192.58 (7919.74-15263.46) | 23.31 (16.51-31.76) | -2.33 (-2.5 to -2.17) |
| Federal Democratic Republic of Nepal | 8277.44 (5590.5-11655.15) | 79.08 (54.02-110.21) | 13783.15 (9969.53-19375.43) | 55.92 (40.68-78.51) | -1.09 (-1.35 to -0.82) |
| Federal Republic of Germany | 55317.05 (50865.8-60258.96) | 48.35 (44.36-52.5) | 36452.1 (32696.5-40366.57) | 21.54 (19.38-23.8) | -2.73 (-2.82 to -2.65) |
| Federal Republic of Nigeria | 17660.25 (12025.26-24162.69) | 36.96 (25.43-50.22) | 28034.57 (18696.14-39065.27) | 27.43 (18.89-37.47) | -0.99 (-1.14 to -0.84) |
| Federal Republic of Somalia | 1804.33 (1144.25-2693.04) | 60.17 (38.98-87.39) | 3429.8 (2097.7-5126.36) | 44.96 (27.96-66.07) | -1.03 (-1.09 to -0.98) |
| Federated States of Micronesia | 10.55 (7.81-14.36) | 20.67 (15.47-27.95) | 13.5 (9.63-18.72) | 16.72 (12.11-23) | -0.82 (-0.88 to -0.76) |
| Federative Republic of Brazil | 76381.3 (73344.09-79314.73) | 78.39 (75.26-81.46) | 152699.49 (143460.67-161454.61) | 59.02 (55.45-62.39) | -0.94 (-1.05 to -0.84) |
| French Republic | 84328.29 (77440.07-92469.73) | 116.39 (107.34-127.45) | 41292.64 (36525.37-46320.46) | 35.58 (31.53-39.79) | -3.68 (-4.05 to -3.32) |
| Gabonese Republic | 317.11 (231.75-422.46) | 53.08 (38.99-70.33) | 483.97 (338.8-648.32) | 41.24 (29.28-54.99) | -0.87 (-0.93 to -0.82) |
| Georgia | 7678.9 (7051.62-8338.56) | 118 (108.32-128.2) | 4806.63 (4164.86-5502.04) | 84.43 (73.11-96.63) | -0.63 (-0.91 to -0.35) |
| Grand Duchy of Luxembourg | 379.1 (349.17-410.05) | 73.11 (67.39-78.76) | 247.15 (220.42-275.67) | 24.37 (21.74-27.18) | -3.49 (-3.65 to -3.33) |
| Greenland | 29.19 (23.2-36.29) | 71.12 (57.35-86.59) | 23.78 (18.75-30.96) | 30.62 (23.97-39.71) | -2.35 (-2.56 to -2.13) |
| Grenada | 26.33 (23.29-29.74) | 40.2 (35.3-45.6) | 36.16 (30.57-42.64) | 29.27 (24.86-34.46) | -0.49 (-0.98 to 0.01) |
| Guam | 10.47 (8.94-12.02) | 12.91 (10.83-14.81) | 13.21 (11.27-15.45) | 6.5 (5.57-7.57) | -2.3 (-2.6 to -2.01) |
| Hashemite Kingdom of Jordan | 594.36 (453.9-758.94) | 39.14 (29.7-50.35) | 1434.96 (1021.83-2014.58) | 16.9 (11.99-23.55) | -3.36 (-3.69 to -3.03) |
| Hellenic Republic | 10121.71 (9551.2-10689.72) | 67.62 (63.9-71.32) | 8546.38 (7836.07-9267.88) | 42.53 (39.05-46.14) | -1.42 (-1.5 to -1.34) |
| Hungary | 19038.12 (16966.21-21110.65) | 136.85 (121.68-151.96) | 13268.4 (10935.54-15920.28) | 79.54 (65.41-96.01) | -2.24 (-2.43 to -2.05) |
| Independent State of Papua New Guinea | 246.75 (157.18-357.95) | 13.07 (8.61-18.69) | 610.68 (420.12-902.49) | 11.53 (7.97-16.78) | -0.45 (-0.51 to -0.4) |
| Independent State of Samoa | 6.79 (5.14-8.94) | 7.49 (5.73-9.74) | 10.1 (7.73-13.76) | 6.52 (5.03-8.82) | -0.52 (-0.61 to -0.42) |
| Ireland | 1842.17 (1688.02-2029.48) | 47.44 (43.29-52.17) | 1285.59 (1130.44-1456.74) | 17.15 (15.09-19.54) | -2.95 (-3.1 to -2.8) |
| Islamic Republic of Afghanistan | 6293.67 (3274.48-10118.83) | 85.58 (45.62-135.35) | 8502.42 (4484.39-13451.95) | 70.97 (38.82-108.03) | -0.74 (-0.85 to -0.62) |
| Islamic Republic of Iran | 19773.94 (16506.85-22427.76) | 68.26 (56.86-77.64) | 36206.04 (32163.94-40642.78) | 44.15 (39.28-49.52) | -1.25 (-1.33 to -1.17) |
| Islamic Republic of Mauritania | 248.05 (193.96-316.29) | 24.01 (18.8-30.31) | 522.89 (357.57-748.31) | 23.24 (16.11-33.01) | 0.07 (-0.14 to 0.29) |
| Islamic Republic of Pakistan | 79632.55 (64276.08-97883.94) | 132.92 (107.23-163.79) | 169489.27 (124982.52-227793.33) | 121.47 (88.73-161.68) | -0.61 (-0.86 to -0.36) |
| Jamaica | 502.82 (448.47-560.68) | 29.29 (26.09-32.69) | 966.03 (696.36-1293.5) | 31.4 (22.62-41.93) | 0.25 (-0.21 to 0.72) |
| Japan | 23295.27 (22361.92-24202.42) | 13.51 (12.95-14.04) | 19627.42 (17845.79-20995.78) | 5.51 (5.13-5.87) | -3.05 (-3.23 to -2.88) |
| Kingdom of Bahrain | 134.14 (108.44-171.17) | 70.98 (57.68-89.71) | 307.61 (217.89-445.05) | 30.51 (22.02-43.15) | -3.51 (-3.77 to -3.24) |
| Kingdom of Belgium | 10774.87 (9952.44-11608.82) | 76.97 (71.24-83.03) | 5268.17 (4736.7-5817.62) | 25.88 (23.47-28.47) | -3.65 (-3.76 to -3.53) |
| Kingdom of Bhutan | 188.25 (120.55-273.19) | 67.45 (43.78-97.39) | 299.58 (204.64-417.81) | 47.62 (32.68-66.34) | -1.16 (-1.27 to -1.06) |
| Kingdom of Cambodia | 2284.26 (1675.04-3067.03) | 46.81 (34.75-62.47) | 4964.99 (3446.6-7525.79) | 37.26 (25.97-56.2) | -0.86 (-1.01 to -0.71) |
| Kingdom of Denmark | 3852.4 (3515.6-4201.26) | 53.5 (48.81-58.31) | 2385.61 (2168.09-2630.43) | 22.26 (20.21-24.45) | -3.02 (-3.15 to -2.9) |
| Kingdom of Eswatini | 254.62 (163.85-358.74) | 78.12 (49.86-108.09) | 484.48 (282.09-715.05) | 73.54 (43.25-107.77) | 0.06 (-0.41 to 0.52) |
| Kingdom of Lesotho | 491.76 (373.15-635.84) | 55.01 (41.84-71.08) | 1078.99 (715.3-1500.23) | 89.44 (59.67-123.47) | 2.04 (1.67 to 2.42) |
| Kingdom of Morocco | 7694.23 (5899.19-9703.89) | 51.7 (39.63-64.8) | 16197.24 (11089.06-21484.08) | 44.41 (30.83-58.43) | -0.37 (-0.53 to -0.22) |
| Kingdom of Norway | 1084.07 (1015.93-1148.15) | 17.55 (16.51-18.6) | 819.61 (742.22-896.03) | 8.47 (7.73-9.24) | -2.56 (-2.79 to -2.33) |
| Kingdom of Saudi Arabia | 1521.99 (1103.12-2026.15) | 23.33 (17.24-30.53) | 3999.41 (2972.53-5511.11) | 15.64 (12.13-20.73) | -1.54 (-1.66 to -1.42) |
| Kingdom of Spain | 60275.75 (52963.3-68108.66) | 118.91 (104.73-134.34) | 33307.67 (29015.31-38419.36) | 38.77 (33.58-44.95) | -3.89 (-4.03 to -3.75) |
| Kingdom of Sweden | 1628.47 (1482.07-1779.52) | 11.63 (10.59-12.75) | 1270.38 (1092.87-1468.43) | 6.21 (5.3-7.2) | -1.65 (-1.89 to -1.41) |
| Kingdom of Thailand | 19801.75 (15999.58-23818.12) | 51.35 (41.52-61.54) | 35607.36 (25970.99-46962.35) | 33 (24.19-43.18) | -1.81 (-1.94 to -1.67) |
| Kingdom of Tonga | 7.23 (5.26-10.06) | 12.96 (9.46-18.08) | 9.34 (6.63-13.06) | 11.55 (8.19-16.16) | -0.43 (-0.64 to -0.23) |
| Kingdom of the Netherlands | 6941.27 (6432.03-7484.66) | 36.84 (34.16-39.8) | 5109.84 (4648.95-5598.21) | 15.49 (14.09-17.01) | -2.94 (-3.07 to -2.81) |
| Kyrgyz Republic | 1912.37 (1595.09-2270.24) | 59.83 (49.89-71.41) | 1149.8 (851.6-1469.4) | 20.78 (15.43-26.43) | -3.47 (-3.66 to -3.28) |
| Lao People's Democratic Republic | 1048.31 (722.57-1470.9) | 46.51 (32.32-64.77) | 1421.86 (949.91-2113.05) | 28.53 (19.44-41.98) | -1.63 (-1.73 to -1.52) |
| Lebanese Republic | 1772.87 (1185.09-2457.09) | 76.26 (51.72-103.82) | 2593.54 (2093.69-3291.16) | 44.2 (35.52-56.25) | -1.46 (-1.62 to -1.3) |
| Malaysia | 3825.47 (2955.02-4638.77) | 39.54 (30.27-48.36) | 9080.35 (7202.85-10874.49) | 30.68 (24.25-36.8) | -1 (-1.24 to -0.76) |
| Mongolia | 472.23 (334.31-621.99) | 41.58 (29.7-54.62) | 625.25 (441.49-873.43) | 22.36 (16.27-30.75) | -2.35 (-2.61 to -2.09) |
| Montenegro | 989.39 (781.52-1243.95) | 149.64 (118.22-188.06) | 1233.22 (944.93-1645.78) | 127.61 (97.86-170.11) | -0.61 (-0.88 to -0.33) |
| New Zealand | 869.32 (787.4-956.35) | 22.51 (20.46-24.71) | 729.63 (646.92-828.54) | 8.81 (7.83-9.97) | -3.03 (-3.28 to -2.77) |
| North Macedonia | 2336.11 (1921.78-2917.32) | 115.86 (95.01-144.76) | 2957.57 (2213.69-3912.01) | 86.15 (64.65-113.76) | -1.09 (-1.35 to -0.82) |
| Northern Mariana Islands | 5.26 (3.59-7.34) | 26.37 (19.07-34.24) | 8.02 (6.53-9.59) | 15.14 (12.33-17.99) | -2.39 (-2.71 to -2.06) |
| Palestine | 300.76 (223.02-398.71) | 33.72 (25.04-44.5) | 596.1 (471.91-741.15) | 21.68 (17.32-26.91) | -1.68 (-1.93 to -1.44) |
| People's Democratic Republic of Algeria | 5797.67 (4395.65-7741.09) | 46.02 (35.36-60.91) | 10624.88 (7708.42-14018.48) | 28.5 (21.05-37.79) | -1.56 (-1.7 to -1.42) |
| People's Republic of Bangladesh | 52907.36 (40937.88-66116.2) | 105.03 (81.68-131.7) | 81431.35 (57309.81-112543.12) | 56.28 (39.91-77.04) | -2.05 (-2.15 to -1.94) |
| People's Republic of China | 362503.08 (295795.93-428646.1) | 40.37 (33.13-47.6) | 493847.71 (382572.07-626010.24) | 22.73 (17.67-28.65) | -1.91 (-2 to -1.81) |
| Plurinational State of Bolivia | 1333.61 (964.26-1747.05) | 39.56 (28.43-51.83) | 2331.76 (1594.76-3339.51) | 24.76 (16.99-35.11) | -1.54 (-1.66 to -1.42) |
| Portuguese Republic | 12758.32 (11618.13-14039.9) | 95.91 (87.33-105.78) | 8565.75 (7614.54-9678.58) | 42.42 (37.83-47.86) | -2.65 (-2.89 to -2.42) |
| Principality of Andorra | 22.12 (15.01-34.28) | 37.57 (25.58-58.16) | 26.99 (16.33-40.98) | 17.7 (10.73-26.84) | -2.25 (-2.45 to -2.05) |
| Principality of Monaco | 86.53 (61.69-116.47) | 151.86 (107.39-205.16) | 71.59 (54.63-96.36) | 90.59 (68.72-125.08) | -1.74 (-1.83 to -1.65) |
| Puerto Rico | 2048.54 (1816.99-2280.81) | 56.8 (50.39-63.16) | 1348.66 (1076-1662.91) | 22.13 (17.46-27.39) | -2.87 (-3.16 to -2.58) |
| Republic of Albania | 1873.5 (1500.05-2304.84) | 84.41 (67.52-104.11) | 2280.96 (1595.31-3289.27) | 54.1 (38.65-77.26) | -1.14 (-1.35 to -0.94) |
| Republic of Angola | 2305.69 (1538.43-3269.84) | 51.67 (34.96-72.56) | 5351.59 (3946.11-7094.49) | 39.35 (29.69-51.05) | -0.85 (-0.98 to -0.72) |
| Republic of Armenia | 3543.77 (3363.11-3691.46) | 116.23 (110.2-121.07) | 1995.48 (1782.83-2242.5) | 46.08 (41.17-51.83) | -3.06 (-3.32 to -2.8) |
| Republic of Austria | 5636.44 (5138.85-6200.47) | 53.67 (48.59-59.45) | 3374.23 (2980.2-3752.24) | 20.69 (18.32-23) | -3.03 (-3.19 to -2.86) |
| Republic of Azerbaijan | 4937.44 (4302.86-5710.82) | 87.95 (76.03-101.65) | 5765.58 (4247.61-8305.8) | 48.69 (35.87-69.88) | -2.05 (-2.19 to -1.91) |
| Republic of Belarus | 14094.44 (12301.7-16244.45) | 107.93 (94.32-123.91) | 10259.85 (7711.5-13157.23) | 66.38 (49.64-84.75) | -2.85 (-3.27 to -2.43) |
| Republic of Benin | 502.9 (396.42-618.85) | 24.62 (19.4-30.36) | 1396.51 (1003.2-1877.89) | 25.22 (18.3-33.52) | 0.33 (0.22 to 0.44) |
| Republic of Botswana | 433.74 (313.86-565.79) | 69.18 (50.38-89.45) | 769.07 (524.67-1235.49) | 45.85 (32.14-72.01) | -1.76 (-2.12 to -1.4) |
| Republic of Bulgaria | 11220.88 (9566.22-12971.37) | 91.61 (78.35-106.5) | 11171.6 (8897.71-13757.04) | 89.58 (71.08-110.54) | 0.08 (-0.18 to 0.33) |
| Republic of Burundi | 1595.71 (1013.46-2241.95) | 63.16 (40.41-88.82) | 2162.61 (1490.73-3155.92) | 37.83 (26.29-54.5) | -2.05 (-2.29 to -1.8) |
| Republic of Cabo Verde | 82.97 (64.99-104.64) | 36.7 (29.06-46.34) | 160.15 (110.33-212.17) | 34.19 (23.56-44.44) | -0.47 (-0.93 to -0.01) |
| Republic of Cameroon | 1304.44 (1012.12-1722.16) | 26.71 (20.83-34.71) | 4420.41 (2843.17-6462.2) | 31.66 (20.77-45.67) | 0.79 (0.62 to 0.95) |
| Republic of Chad | 593.99 (455.92-761.77) | 20.6 (15.83-26.34) | 2091.93 (1450.57-2821.16) | 33.63 (23.5-45.2) | 1.87 (1.68 to 2.07) |
| Republic of Chile | 4044.23 (3662.12-4461.5) | 38.85 (35.22-42.96) | 3798.78 (3414.64-4234.14) | 14.95 (13.45-16.66) | -2.94 (-3.05 to -2.83) |
| Republic of Colombia | 11061.05 (10136.42-11857.48) | 59.91 (54.87-64.33) | 10807.01 (8666.67-13384.62) | 19.59 (15.76-24.24) | -4.22 (-4.41 to -4.04) |
| Republic of Costa Rica | 736.12 (662.45-808.64) | 41.55 (37.42-45.67) | 916.85 (788.35-1068.43) | 16.59 (14.26-19.34) | -3.13 (-3.34 to -2.92) |
| Republic of Croatia | 8283.41 (7482.62-9120) | 127.95 (115.47-141.2) | 4412.45 (3706.09-5132.66) | 55.67 (46.73-64.51) | -2.76 (-2.93 to -2.58) |
| Republic of Cuba | 10699.01 (9684.12-11861.59) | 104.64 (94.65-116.01) | 22741.92 (18945.1-27640.45) | 117.58 (97.77-142.57) | 0.56 (0.41 to 0.71) |
| Republic of Cyprus | 279.46 (223.84-345.52) | 35.64 (28.62-44.07) | 376.16 (295.76-487.33) | 18.7 (14.69-24.18) | -1.91 (-1.98 to -1.84) |
| Republic of C么te d'Ivoire | 1602.89 (1244.95-2090.97) | 34.34 (26.95-44.03) | 3893.5 (2652.53-6130.65) | 29.74 (20.6-45.91) | -0.73 (-0.93 to -0.54) |
| Republic of Djibouti | 92.4 (62.51-142.27) | 54.56 (37.66-82.46) | 357.1 (235.03-517.74) | 45.66 (30.9-64.09) | -0.72 (-0.84 to -0.59) |
| Republic of Ecuador | 1384.73 (1283.86-1499.44) | 24.94 (23.05-27.08) | 2077.38 (1590.34-2688.86) | 12.55 (9.62-16.23) | -2.25 (-2.52 to -1.99) |
| Republic of El Salvador | 716.23 (615.06-832.42) | 23.58 (20.22-27.49) | 1008.47 (776.13-1297.04) | 16.59 (12.76-21.4) | -1.5 (-1.72 to -1.28) |
| Republic of Equatorial Guinea | 113.32 (66.41-166.12) | 52.08 (31.45-75.23) | 179.33 (104.75-271.38) | 31.17 (18.43-45.99) | -1.85 (-2.22 to -1.47) |
| Republic of Estonia | 1526.97 (1347.44-1728.48) | 75.35 (66.71-85.12) | 758.96 (618.36-929.44) | 33.37 (26.91-41.01) | -3.36 (-3.69 to -3.03) |
| Republic of Fiji | 68.21 (53.2-87.77) | 17.71 (13.78-22.68) | 130.08 (94.4-174.07) | 16.14 (11.7-21.12) | -0.18 (-0.32 to -0.03) |
| Republic of Finland | 1527.3 (1394.73-1677.5) | 22.72 (20.7-25.13) | 1174.02 (1052.63-1308.09) | 11.05 (9.93-12.3) | -2.37 (-2.61 to -2.13) |
| Republic of Ghana | 1422.5 (1075.08-1898.17) | 20.79 (16.02-27.37) | 5046.47 (3569.79-6667.9) | 27.53 (19.56-36.04) | 1.55 (1.29 to 1.8) |
| Republic of Guatemala | 1375.61 (1313.91-1448.86) | 38.17 (36.42-40.23) | 1312.53 (1114.78-1536.21) | 11.61 (9.87-13.61) | -4.02 (-4.23 to -3.81) |
| Republic of Guinea | 732.18 (551.97-944.11) | 21.38 (16.13-27.58) | 1848.52 (1326.14-2453.46) | 31.12 (22.54-40.88) | 1.74 (1.55 to 1.93) |
| Republic of Guinea-Bissau | 160.57 (100.16-228.63) | 37.32 (23.55-52.98) | 331.38 (222.89-456.02) | 40.06 (26.73-54.84) | 0.66 (0.52 to 0.81) |
| Republic of Guyana | 126.46 (108.65-147.46) | 31.53 (27.06-36.8) | 207.43 (152.62-276) | 29.22 (21.71-38.59) | 0.34 (0.11 to 0.57) |
| Republic of Haiti | 2598.77 (1743.22-3461.76) | 74.01 (50.03-97.86) | 4543.7 (2950.53-6476.97) | 57.84 (37.34-81.36) | -0.63 (-0.74 to -0.53) |
| Republic of Honduras | 619.88 (503.81-774.43) | 28.82 (23.64-35.87) | 2184.3 (1670.13-2859.22) | 33.52 (25.27-43.43) | 0.7 (0.59 to 0.81) |
| Republic of Iceland | 57.88 (52.08-64.13) | 21.52 (19.34-23.88) | 51.28 (44.47-58.4) | 9.63 (8.38-10.97) | -2.78 (-2.97 to -2.58) |
| Republic of India | 434764.82 (356863-520651.2) | 82.09 (66.95-98.23) | 801556.91 (690614.52-926887.08) | 62.95 (54.37-72.82) | -0.98 (-1.12 to -0.84) |
| Republic of Indonesia | 30390.25 (23072.19-36886.23) | 28.01 (21.1-34.11) | 65905.47 (47528.07-87715.66) | 25.21 (18.01-33.31) | -0.33 (-0.37 to -0.29) |
| Republic of Iraq | 5611.08 (4368.32-7190.29) | 66.88 (51.49-85.6) | 13253.06 (9422.08-17522.93) | 50.56 (35.99-67.05) | -1.25 (-1.35 to -1.15) |
| Republic of Italy | 66609.11 (63836.1-69658.67) | 78.4 (75.14-82) | 34775.86 (32033.94-37434.11) | 26.96 (25.19-29.05) | -3.42 (-3.53 to -3.31) |
| Republic of Kazakhstan | 13411.64 (12369.18-14442.87) | 96.4 (88.91-103.84) | 6665.64 (5727.83-7626.36) | 33.24 (28.57-38.05) | -4.14 (-4.45 to -3.82) |
| Republic of Kenya | 2807.02 (2046.81-3819.71) | 30.94 (22.44-42.19) | 9276.78 (6882.44-12152.82) | 34.89 (25.69-45.22) | 0.5 (0.25 to 0.74) |
| Republic of Kiribati | 2.11 (1.63-2.78) | 5.33 (4.16-6.98) | 4.4 (3.06-6.38) | 5.47 (3.91-7.84) | 0.06 (-0.05 to 0.16) |
| Republic of Korea | 18153.56 (12777.14-23075.32) | 57.12 (40.47-72.48) | 11113.33 (8265.25-14290.79) | 11.7 (8.7-15.01) | -5.95 (-6.28 to -5.62) |
| Republic of Latvia | 3270.69 (2926.46-3691.18) | 92.71 (82.68-105.17) | 1639.15 (1356.17-1978.33) | 49.73 (40.99-60.21) | -2.37 (-2.59 to -2.16) |
| Republic of Liberia | 275.22 (196.25-402.23) | 23.28 (16.66-34.1) | 603.33 (399.92-880.75) | 25.86 (17.33-36.85) | 0.53 (0.36 to 0.69) |
| Republic of Lithuania | 4584.05 (3994.93-5268.38) | 102.48 (89.45-117.7) | 2966.7 (2332.61-3650.54) | 60.78 (47.67-74.35) | -1.94 (-2.2 to -1.68) |
| Republic of Madagascar | 2474.29 (1796.68-3083.71) | 43.9 (32.3-55) | 3979.01 (2798.23-5431.32) | 28.66 (20.24-38.43) | -1.42 (-1.54 to -1.3) |
| Republic of Malawi | 685.5 (530.76-915.08) | 15.8 (12.26-21.13) | 1379.09 (1019.71-1977.22) | 15.87 (11.85-22.32) | -0.24 (-0.45 to -0.03) |
| Republic of Maldives | 27.83 (18.51-36.52) | 29.57 (21.07-38.03) | 39.37 (28.48-52.04) | 10.92 (8.04-14.24) | -3.6 (-3.78 to -3.41) |
| Republic of Mali | 1248.2 (1024.75-1534.46) | 28.28 (23.49-34.4) | 2491.41 (1815.85-3532.28) | 25.15 (18.49-34.83) | -0.1 (-0.26 to 0.05) |
| Republic of Malta | 221.18 (192.16-250.94) | 51.64 (44.8-58.73) | 173.21 (145.76-205.86) | 21.42 (18.01-25.46) | -2.88 (-2.95 to -2.8) |
| Republic of Mauritius | 418.43 (392.25-453.08) | 53.8 (50.4-58.18) | 715.85 (647.56-771.91) | 37.82 (34.29-40.6) | -0.81 (-1.6 to -0.02) |
| Republic of Moldova | 4869.8 (4591.35-5175.04) | 104.34 (98.44-110.65) | 4280.01 (3791.12-4856.58) | 72.89 (64.66-82.56) | -1.22 (-1.49 to -0.94) |
| Republic of Mozambique | 3402.87 (2410.73-4546.04) | 50.31 (36.68-67.47) | 6673.26 (4823.3-8871.64) | 50.27 (36.61-66.06) | 0.41 (0.25 to 0.56) |
| Republic of Namibia | 496.03 (397.34-615.17) | 68.66 (55.01-85.29) | 1072.09 (762.4-1466.07) | 67.74 (48.71-91.69) | -0.23 (-0.54 to 0.08) |
| Republic of Nauru | 1.24 (0.77-1.7) | 25.67 (16.59-34.53) | 1.19 (0.74-1.7) | 18.9 (12.27-26.6) | -1.13 (-1.38 to -0.88) |
| Republic of Nicaragua | 460.25 (374.79-561.59) | 28.99 (23.49-35.29) | 904.45 (696-1182.11) | 18 (13.88-23.52) | -1.61 (-1.76 to -1.45) |
| Republic of Niue | 0.28 (0.2-0.37) | 13.17 (9.46-17.31) | 0.25 (0.19-0.34) | 11.62 (8.56-15.53) | -0.67 (-0.82 to -0.52) |
| Republic of Palau | 1.44 (1.01-1.91) | 14.1 (10.07-18.67) | 2.65 (1.9-3.46) | 11.08 (7.94-14.63) | -0.87 (-0.97 to -0.76) |
| Republic of Panama | 550.42 (514.55-584.29) | 36.83 (34.3-39.08) | 720.83 (556.42-884.96) | 16.36 (12.62-20.09) | -2.86 (-3.07 to -2.65) |
| Republic of Paraguay | 803.31 (619.42-1040.85) | 34.83 (26.88-44.97) | 2626.33 (1785.72-3647.17) | 43.21 (29.54-59.86) | 0.82 (0.72 to 0.92) |
| Republic of Peru | 3405.37 (2727.09-4117.72) | 27.54 (22.11-33.4) | 4346.4 (3085.26-6045.41) | 12.89 (9.11-17.92) | -2.92 (-3.31 to -2.53) |
| Republic of Poland | 54790.43 (52763.84-56799.53) | 126.53 (121.93-131.06) | 45062.17 (40442.44-49615.15) | 67.32 (60.35-74.18) | -2.25 (-2.42 to -2.08) |
| Republic of Rwanda | 2350.62 (1653.63-3041.83) | 73.47 (52.15-94.96) | 2797.48 (1869.78-4179.87) | 38.65 (26.17-57.17) | -3.04 (-3.39 to -2.68) |
| Republic of San Marino | 19.15 (14.68-24.05) | 58.64 (44.79-73.71) | 14.1 (8.46-20.81) | 21.94 (12.84-32.87) | -2.43 (-2.72 to -2.14) |
| Republic of Senegal | 899.76 (705.19-1097.99) | 26.27 (20.73-32.01) | 2358.96 (1737.57-3128.12) | 28.56 (20.82-37.65) | 0.61 (0.41 to 0.8) |
| Republic of Serbia | 13957.94 (9983.94-19984.06) | 114.44 (81.84-162.55) | 11633.14 (7953.18-16181.36) | 77.06 (52.51-107.73) | -1.52 (-1.76 to -1.28) |
| Republic of Seychelles | 77.13 (65.66-90.8) | 137.92 (116.9-162.74) | 125.65 (101.92-152.94) | 100.72 (81.77-121.39) | -0.36 (-0.63 to -0.09) |
| Republic of Sierra Leone | 530.3 (393.34-700.32) | 25.34 (18.9-33.35) | 1117.16 (779.87-1598.86) | 27.58 (19.45-39.26) | 0.75 (0.56 to 0.95) |
| Republic of Singapore | 869.35 (785.06-953.61) | 38.1 (34.3-41.9) | 786.06 (685.19-893.1) | 9.07 (7.91-10.24) | -5.15 (-5.48 to -4.81) |
| Republic of Slovenia | 1929.39 (1746.18-2151.5) | 78.25 (70.59-87.14) | 1290.61 (1072.5-1537.73) | 33.1 (27.51-39.67) | -3.01 (-3.22 to -2.8) |
| Republic of South Africa | 12379.85 (10123.58-16565.25) | 54.53 (44.31-73.9) | 22169.93 (19388.38-25175.73) | 43.71 (38.32-49.53) | -1.03 (-1.35 to -0.71) |
| Republic of South Sudan | 1476.46 (1010.85-2003.27) | 54.02 (37.23-73.37) | 1902.33 (1221.53-2829.48) | 41.26 (26.62-60.72) | -1.12 (-1.31 to -0.92) |
| Republic of Sudan | 6230.55 (3836.92-9496.03) | 62.49 (38.4-97.36) | 10050.8 (5977.07-15138.82) | 45.94 (28.28-68.29) | -1.09 (-1.13 to -1.05) |
| Republic of Suriname | 61.91 (50.97-73.62) | 22.95 (18.86-27.3) | 138.7 (98.19-192.4) | 20.72 (14.7-28.94) | -0.16 (-0.35 to 0.04) |
| Republic of Tajikistan | 1380.01 (1018.79-1750.16) | 46.06 (34.15-58.65) | 1688 (1125.19-2411.99) | 24.04 (16.28-33.99) | -2.26 (-2.46 to -2.07) |
| Republic of Trinidad and Tobago | 310.32 (288.24-332.93) | 36.42 (33.81-39.08) | 590.09 (447.66-767.01) | 30.13 (22.91-39.14) | -0.74 (-0.9 to -0.59) |
| Republic of Tunisia | 3556.5 (2686.47-4590.68) | 67.34 (51.58-86.12) | 7061.48 (4877.3-10025.42) | 51 (35.44-72.13) | -1.09 (-1.16 to -1.02) |
| Republic of Turkey | 35531.8 (25543.45-47971.18) | 94.95 (68.71-127.5) | 41862.87 (32244.41-54036.53) | 43.3 (33.49-55.67) | -2.84 (-3.03 to -2.65) |
| Republic of Uganda | 4232.92 (3113.77-5486.89) | 59.89 (44.58-76.97) | 8113.73 (5419.99-11715.85) | 46.99 (32.35-66.35) | -1.53 (-1.81 to -1.24) |
| Republic of Uzbekistan | 7827.19 (6774.79-9021.91) | 63.01 (54.78-72.37) | 5340.65 (4151.22-6849.27) | 17.79 (14.03-22.45) | -3.83 (-4.47 to -3.19) |
| Republic of Vanuatu | 9.18 (5.77-13.52) | 14.07 (9.25-19.95) | 21.15 (14.51-30.05) | 11.48 (8.06-15.9) | -0.85 (-0.95 to -0.75) |
| Republic of Yemen | 3979.61 (2455.16-5695.49) | 72.21 (44.78-102.7) | 9455.2 (5737.79-13850.73) | 60.21 (37.12-87.82) | -0.69 (-0.76 to -0.63) |
| Republic of Zambia | 1836.63 (1360.64-2344.94) | 56.97 (42.48-72.05) | 4904.77 (2289.21-11548.61) | 57.41 (28.38-130.81) | -0.18 (-0.32 to -0.05) |
| Republic of Zimbabwe | 2466.56 (1858.11-3119.89) | 55.3 (42.13-69.78) | 5316.93 (3889.51-7345.88) | 63.81 (47.84-85.88) | 0.59 (0.28 to 0.9) |
| Republic of the Congo | 660.48 (412.56-882) | 56.2 (35.49-74.2) | 1264.15 (907.32-1711.27) | 40.66 (29.95-54.07) | -1.27 (-1.43 to -1.1) |
| Republic of the Gambia | 52.22 (39.2-69.12) | 13.41 (10.17-17.55) | 132.15 (98.08-173.87) | 12.26 (9.06-15.91) | -0.44 (-0.61 to -0.27) |
| Republic of the Marshall Islands | 3.37 (2.35-4.7) | 19.32 (13.62-26.7) | 6.82 (4.44-9.84) | 18.2 (11.85-26.12) | -0.13 (-0.26 to 0.01) |
| Republic of the Niger | 689.46 (501.73-910.18) | 22.57 (16.56-29.8) | 2027.32 (1354.56-2894.52) | 23 (15.52-32.65) | 0.46 (0.3 to 0.61) |
| Republic of the Philippines | 7959.35 (6671.65-9616.38) | 24.31 (20.3-29.64) | 19062.05 (15250.17-23109.81) | 21.43 (17.18-25.84) | -0.48 (-0.58 to -0.38) |
| Republic of the Union of Myanmar | 10685.22 (6529.41-14871.49) | 42.76 (27.02-58.98) | 11481.08 (7982.45-16352.65) | 22.31 (15.71-31.62) | -2.31 (-2.42 to -2.2) |
| Romania | 28390.86 (25014.46-31762.81) | 99.26 (87.57-111.09) | 29021.83 (24628.65-33834.55) | 89.82 (75.76-104.84) | -0.56 (-0.73 to -0.38) |
| Russian Federation | 195828.04 (190187.42-200853.03) | 105.85 (102.84-108.72) | 113003.12 (100331.44-124395.43) | 48.57 (43.17-53.45) | -3.23 (-3.48 to -2.97) |
| Saint Kitts and Nevis | 14.42 (13.09-15.78) | 41.87 (37.58-45.95) | 30.66 (24.77-37.53) | 39.4 (32-47.74) | 0.2 (-0.06 to 0.47) |
| Saint Lucia | 53.19 (49.72-56.9) | 61.15 (57.19-65.53) | 127.39 (102.31-156.63) | 51.78 (41.65-63.4) | -0.63 (-0.82 to -0.44) |
| Saint Vincent and the Grenadines | 42.9 (38.76-47.36) | 60.57 (54.6-66.92) | 100.15 (86.78-115.87) | 68.31 (59.24-79) | 0.41 (0.22 to 0.6) |
| Slovak Republic | 6971.4 (5216.12-9176.25) | 120.37 (90.28-158.14) | 5230.61 (3656.6-7077.89) | 58.24 (40.33-79.34) | -2.42 (-2.5 to -2.34) |
| Socialist Republic of Viet Nam | 14260.32 (10968.6-18767.06) | 34.43 (26.62-45.17) | 38866.55 (27910.16-52931.67) | 35.88 (26.06-48.19) | 0.19 (0.14 to 0.25) |
| Solomon Islands | 28.85 (16.39-41.96) | 20.08 (12.07-28.73) | 65.62 (46.61-89.82) | 17.19 (12.16-23.67) | -0.5 (-0.57 to -0.43) |
| State of Eritrea | 913.67 (628.47-1278.2) | 60.74 (42.14-83.29) | 1517.11 (1029.45-2118.67) | 43.29 (29.84-59.42) | -1.33 (-1.42 to -1.23) |
| State of Israel | 1446.48 (1312.18-1602.53) | 31.27 (28.26-34.64) | 1995.47 (1757.95-2274.02) | 17.34 (15.37-19.81) | -2.29 (-2.49 to -2.09) |
| State of Kuwait | 292.66 (258.42-327.53) | 39.83 (35.23-44.45) | 302.57 (246.72-375.53) | 9.08 (7.31-11.35) | -3.9 (-4.48 to -3.31) |
| State of Libya | 1701.75 (1235.8-2360.25) | 83.72 (60.89-115.58) | 4714.73 (3347.38-6468.02) | 78.25 (56.08-105.94) | -0.02 (-0.16 to 0.11) |
| State of Qatar | 82.29 (61.67-110.41) | 63.99 (47.84-84.58) | 360.69 (249.75-503.25) | 29.6 (20.52-41.58) | -2.54 (-3.15 to -1.92) |
| Sultanate of Oman | 165.49 (114.52-229.75) | 21.71 (15.1-30.17) | 264.95 (192.23-358.15) | 11.03 (8.29-14.74) | -1.97 (-2.15 to -1.79) |
| Swiss Confederation | 3635.46 (3344.06-3948.07) | 38.72 (35.63-42.04) | 2215.2 (1960.08-2471.82) | 13.6 (12.07-15.18) | -3.16 (-3.35 to -2.96) |
| Syrian Arab Republic | 2162.2 (1651.26-2786.66) | 37.93 (28.83-49.08) | 3888.22 (2780.65-5525.68) | 27.5 (20.05-38.52) | -1.35 (-1.54 to -1.17) |
| Taiwan (Province of China) | 5246.1 (4891.99-5647.23) | 30.72 (28.69-33.03) | 6458.84 (5759.9-7176.32) | 15.62 (13.94-17.36) | -2.52 (-2.73 to -2.31) |
| Togolese Republic | 352.13 (260.3-455.31) | 26.26 (19.57-33.9) | 1418.25 (993.83-1929.08) | 32.37 (22.99-43.78) | 0.93 (0.81 to 1.06) |
| Tokelau | 0.2 (0.14-0.29) | 14.72 (10.39-21.25) | 0.16 (0.11-0.22) | 10.6 (7.5-15.11) | -1.22 (-1.32 to -1.12) |
| Turkmenistan | 1541.28 (1431.8-1651.33) | 71.89 (66.77-76.98) | 1384.86 (1059.41-1827.22) | 29.87 (22.96-39.32) | -3.23 (-3.46 to -2.99) |
| Tuvalu | 1.2 (0.9-1.54) | 16.95 (12.88-21.75) | 1.52 (1.15-1.99) | 14.13 (10.72-18.5) | -0.6 (-0.68 to -0.53) |
| Ukraine | 78026.06 (69922.87-86812.02) | 111.42 (99.51-124.87) | 39337.3 (25431.72-56403.79) | 55.06 (35.34-78.63) | -2.99 (-3.23 to -2.75) |
| Union of the Comoros | 110.31 (71.8-147.81) | 49.54 (33-66.26) | 188.45 (135.41-265.51) | 34.71 (25.12-48.73) | -1.49 (-1.71 to -1.28) |
| United Arab Emirates | 262.88 (163.83-379.46) | 43.83 (27.07-62.26) | 1212.82 (849.75-1650.69) | 23.29 (17.05-29.82) | -1.16 (-1.59 to -0.72) |
| United Kingdom of Great Britain and Northern Ireland | 25476.9 (24796.86-26156.41) | 30.48 (29.71-31.29) | 21253.23 (20261.81-22249.97) | 17.87 (17.12-18.67) | -1.84 (-1.96 to -1.72) |
| United Mexican States | 19910.04 (19386.66-20434.8) | 46.15 (44.87-47.4) | 23395.46 (19837.61-27257.66) | 18.17 (15.44-21.13) | -3.4 (-3.55 to -3.24) |
| United Republic of Tanzania | 6165.85 (4359.76-8625.05) | 51.23 (36.36-71.51) | 9702.56 (6578.92-15347.55) | 33.26 (22.88-51.28) | -1.71 (-1.81 to -1.6) |
| United States Virgin Islands | 40.3 (32.16-51.88) | 43.72 (34.83-56.28) | 44.03 (30.85-59.72) | 26.52 (18.88-35.63) | -1.47 (-1.66 to -1.28) |
| United States of America | 109879.12 (106300.71-113197.58) | 37.06 (35.91-38.15) | 114288.53 (108857.18-119668.04) | 20.4 (19.5-21.33) | -2.23 (-2.3 to -2.15) |
