# Supplementary material for: Global, regional, and national larynx cancer burden and health inequality analysis from 1990 to 2021 with a prediction from 2022 to 2040
Source: Front Oncol. 2025 Jul 18;15:1617613. doi: 10.3389/fonc.2025.1617613 (PMC12351386; doi:10.3389/fonc.2025.1617613)
Supplement: Supplementary file 2 [file DataSheet1.docx]

**Supplementary information**

***Supplementary Figures***


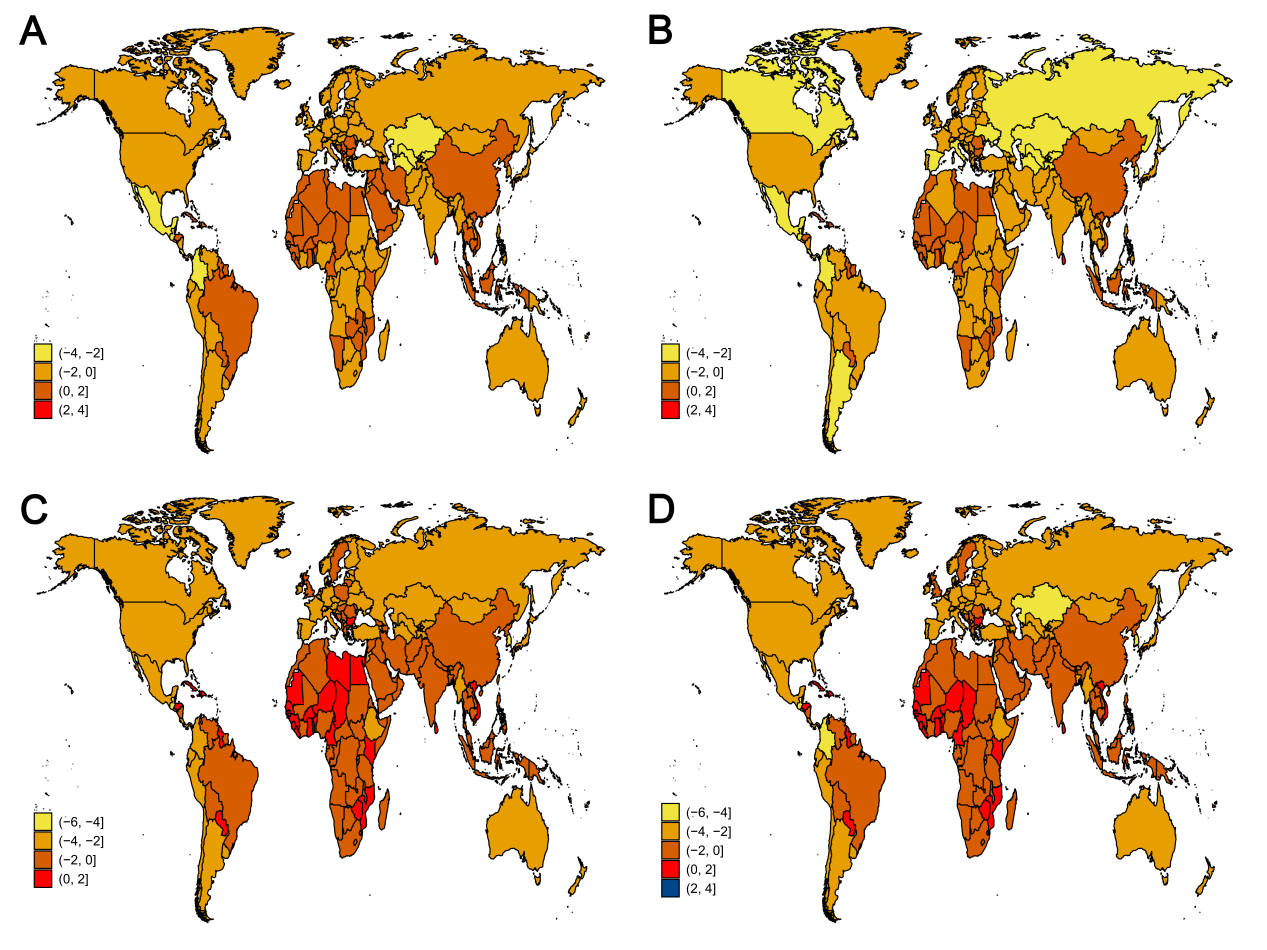


**Fig. S1.**The global disease burden of larynx cancer for both sexes in 204 countries and territories. (A) EAPC for prevalence. (B) EAPC for incidence. (C) EAPC for deaths. (D) EAPC for DALYs.


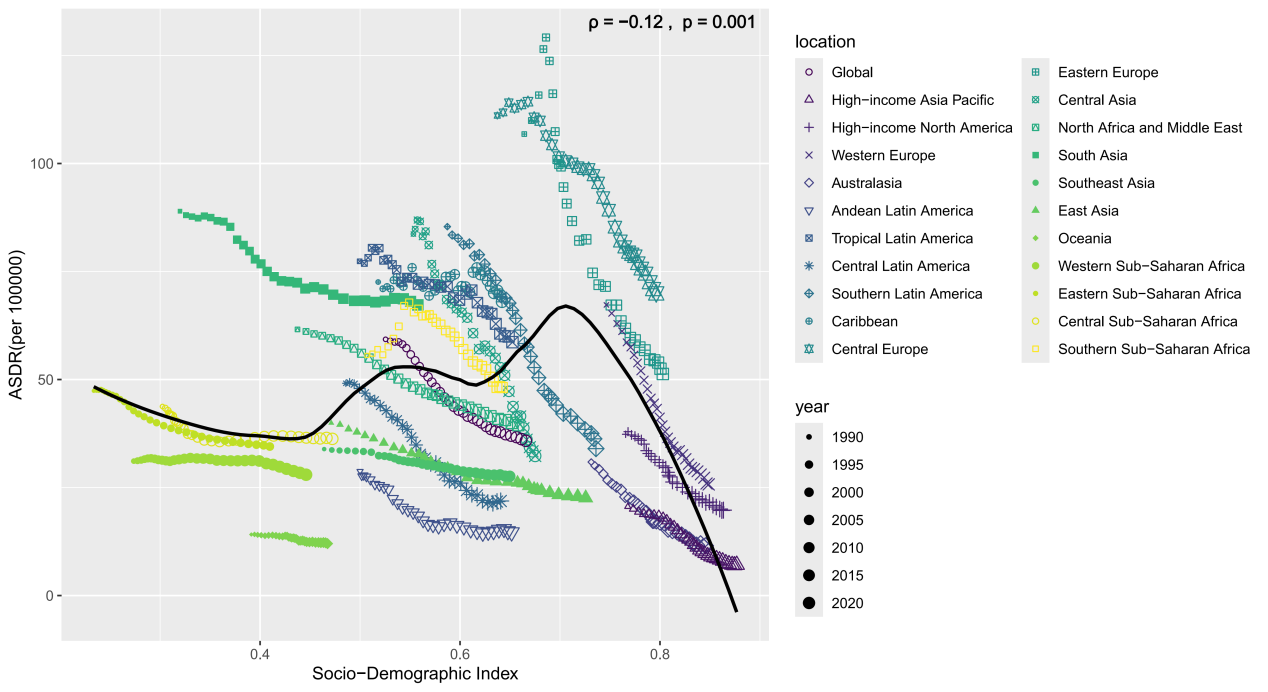


**Fig. S2.** Association between age-standardized larynx cancer DALYs rate and socio-demographic index.


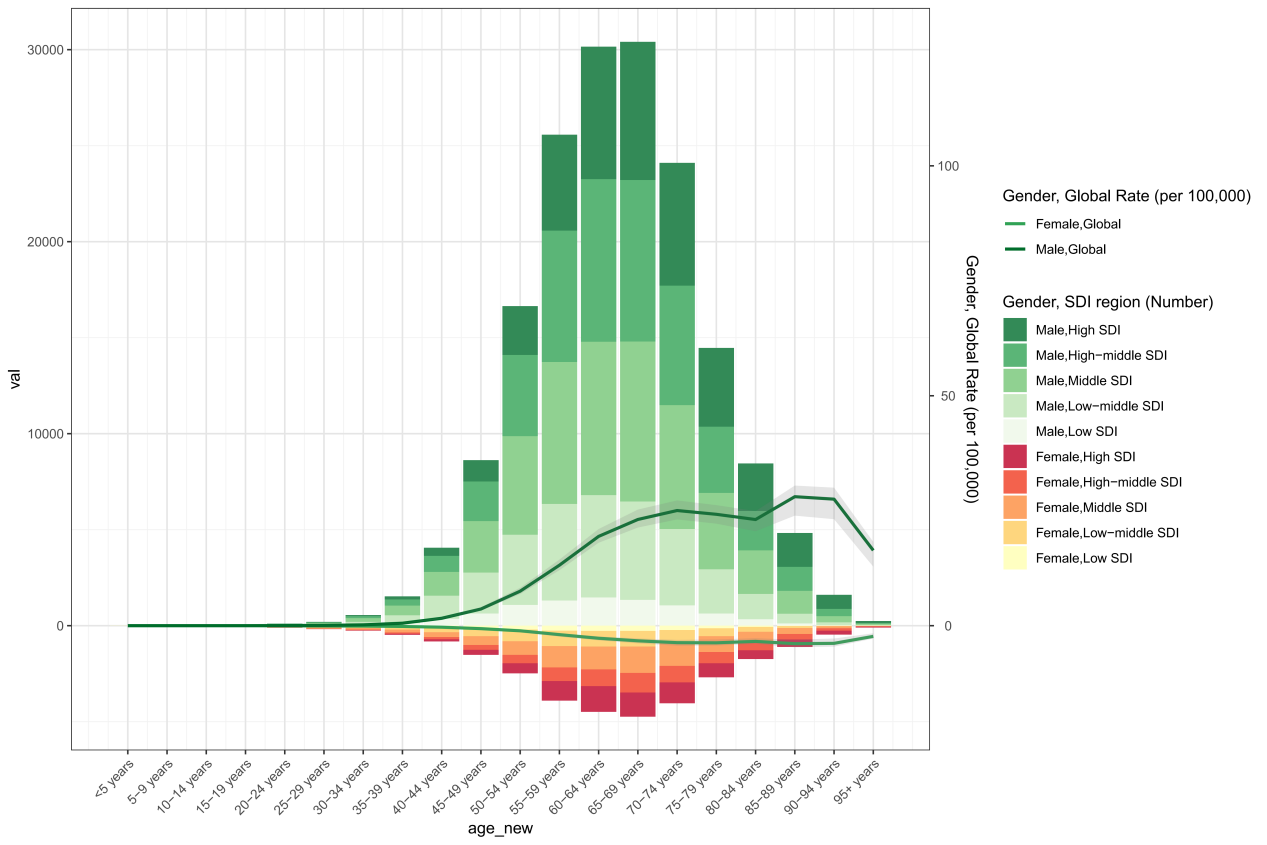


**Fig. S3.** The age-specific numbers and ASIRs of larynx cancer by SDI regions in 1990.


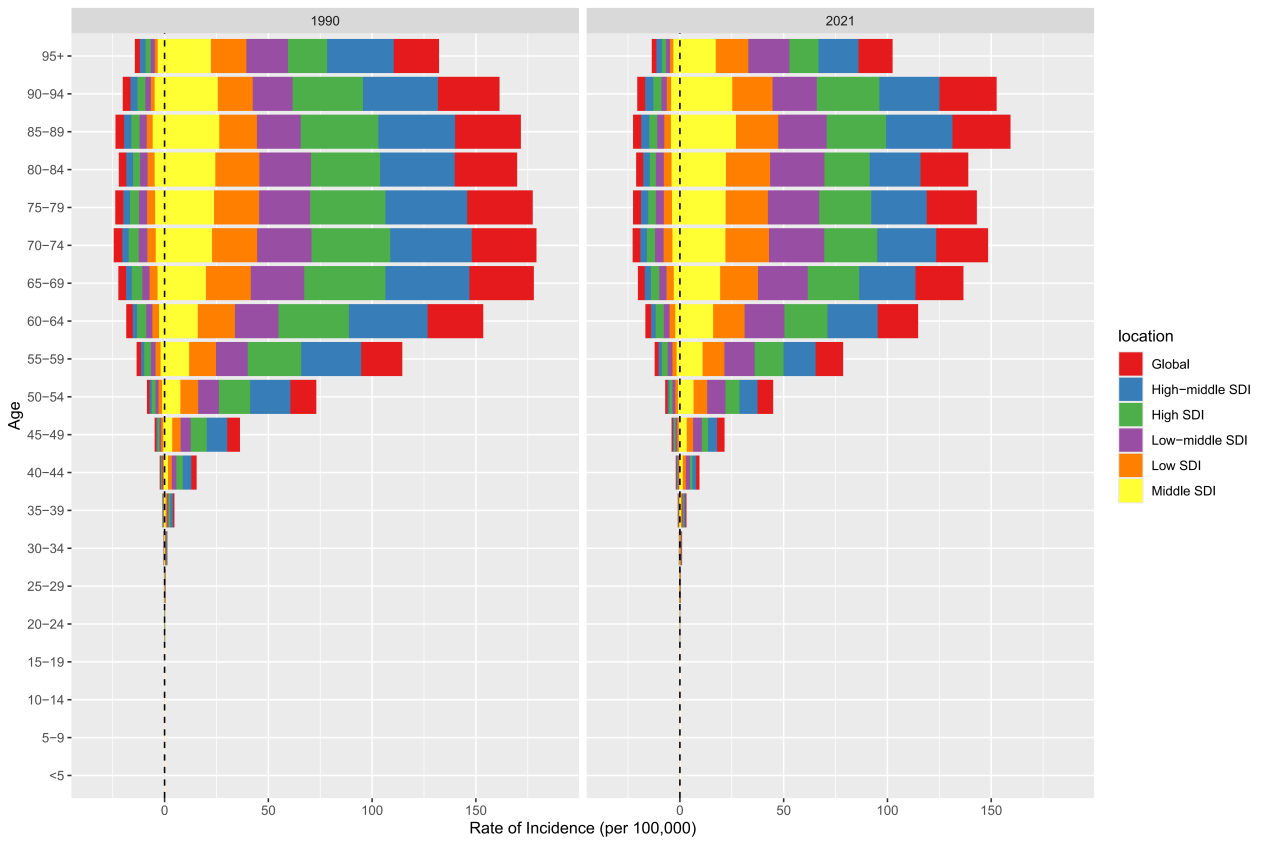


**Fig. S4.** Age-standardized incidence rates of larynx cancer by sex, age group, and socio-demographic index, 1990 and 2021.


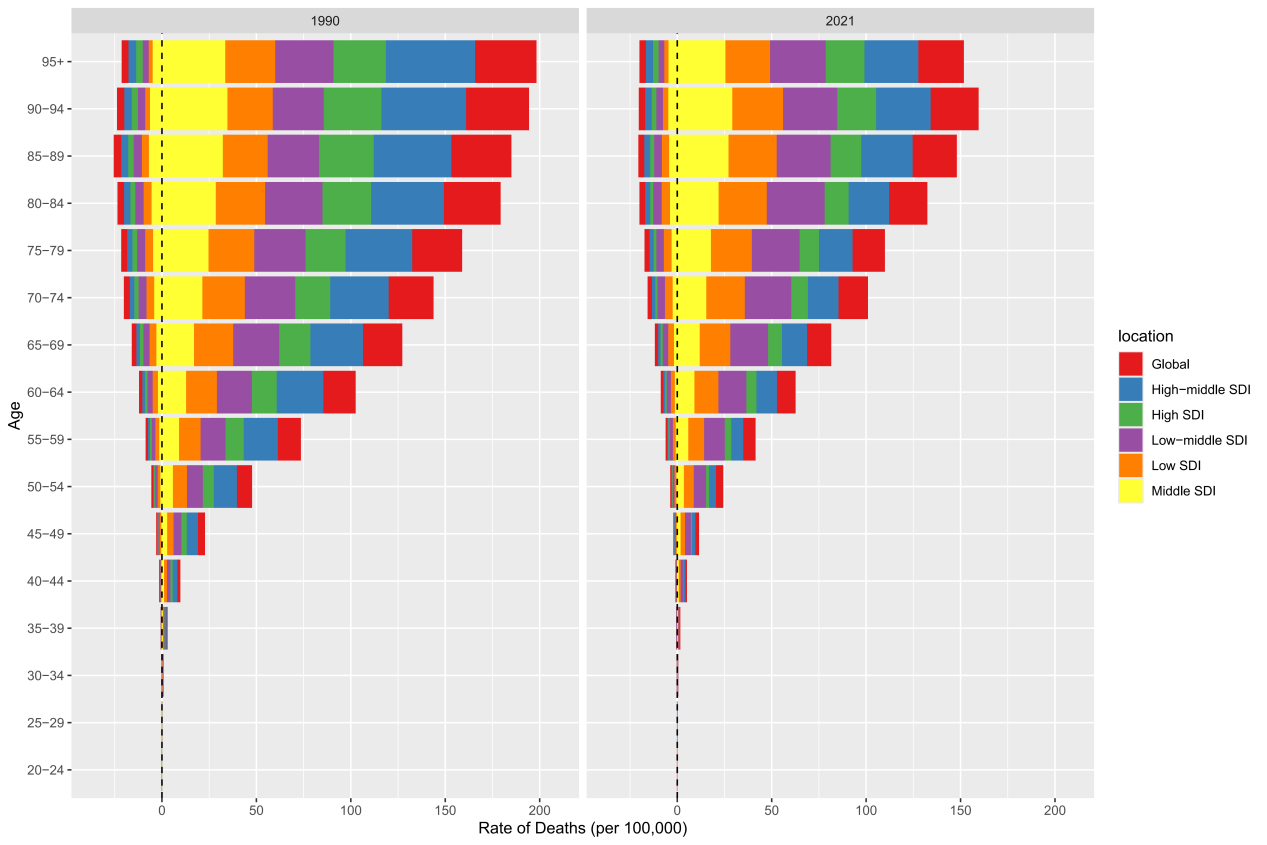


**Fig. S5**. Age-standardized deaths rates of larynx cancer by sex, age group, and socio-demographic index, 1990 and 2021.


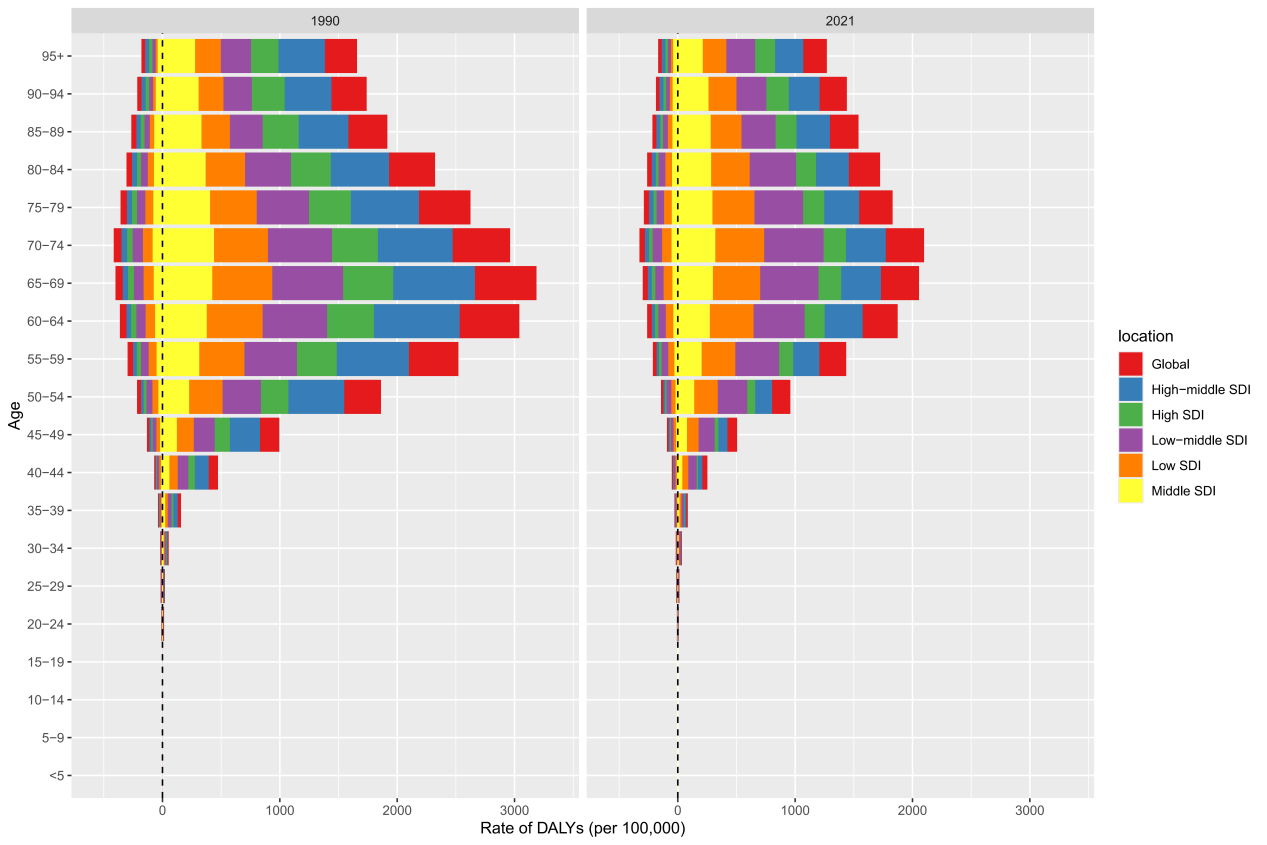


**Fig. S6**. Age-standardized DALYs rates of larynx cancer by sex, age group, and socio-demographic index, 1990 and 2021.


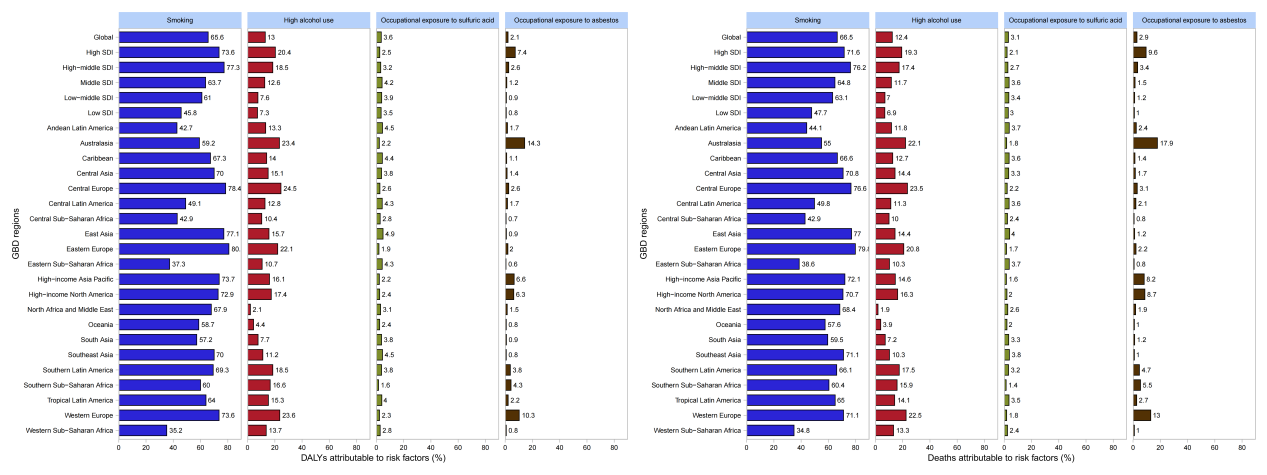


**Fig. S7**. Percentage of age-standardized DALYs rates and ASDR of larynx cancer attributable to smoking, high alcohol use ,Occupational exposure to sulfuric acid and Occupational exposure to asbestos.


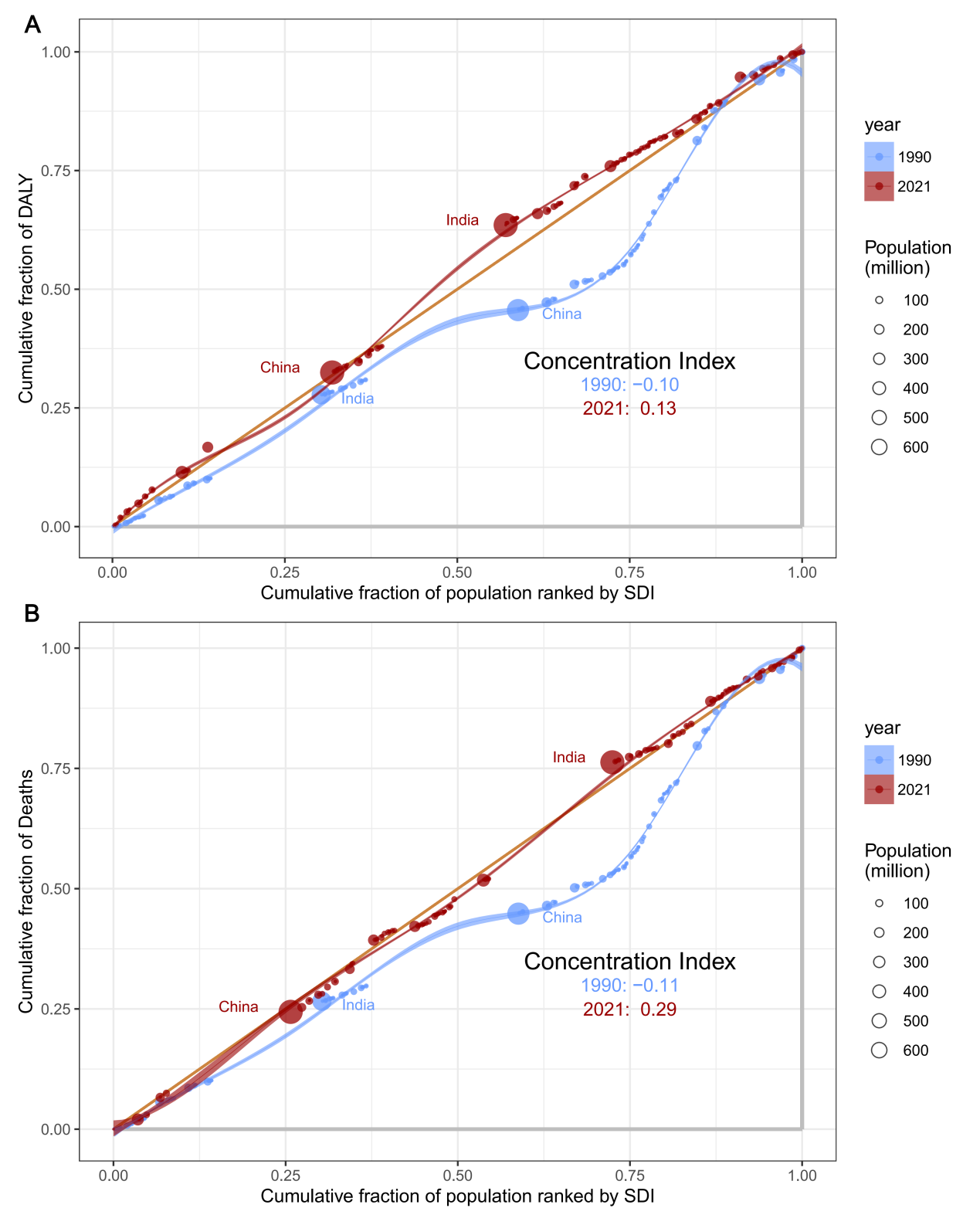


**Fig. S8**.Concentration index analysis. (A) Relative income-related healthy inequality in larynx cancer burden, presented using concentration curves, 1990 vs. 2021. (B) Trendline demonstrates the trend in concentration index from 1990 to 2021


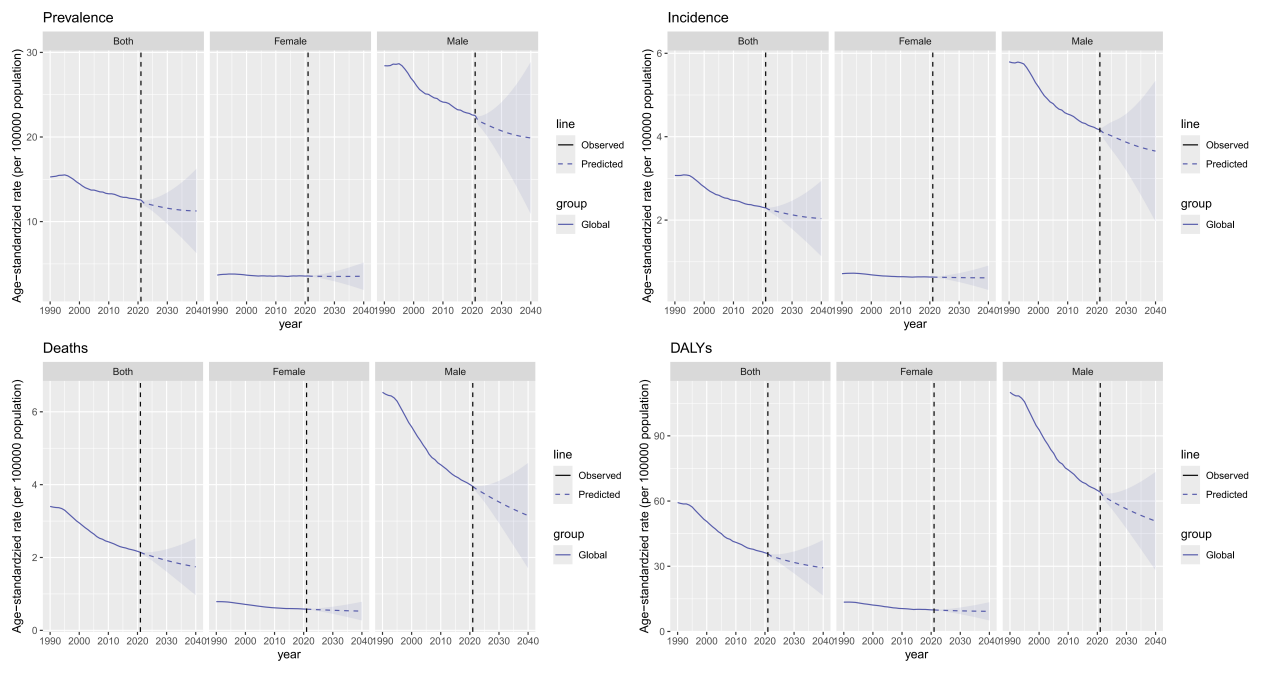


**Fig. S9.** Future forecasts of global burden of larynx cancer.
